# Evaluating the Effectiveness and Technique Composition of Combined Motor Learning and Behavior Change Strategies for Movement Behavior in Chronic Musculoskeletal Pain: A Systematic Review

**DOI:** 10.1016/j.arrct.2026.100618

**Published:** 2026-03-24

**Authors:** Ingrid van Duijvenbode, Matthijs Tuijt, Cindy Veenhof, Bart Visser, Daniel Bossen, Margreet Wortman

**Affiliations:** a Centre of Expertise Urban Vitality, Faculty of Health, Sport and Physical Activity, Amsterdam University of Applied Sciences, Amsterdam; b Department of Human Movement Sciences, Faculty of Behavioral and Movement Sciences, Amsterdam Movement Sciences Research Institute, Vrije Universiteit Amsterdam, Amsterdam; c Department of Human Movement, Research group on Human Movement, Health and Wellbeing, Windesheim University of Applied Sciences, Zwolle; d Research Centre for Healthy and Sustainable Living, Innovation of Movement Care Research Group, Innovation of Human Movement Care, University of Applied Sciences Utrecht, Utrecht; e Department of Rehabilitation, Physical Therapy Science and Sport, Brain Center, University Medical Center Utrecht, Utrecht University, Utrecht, Netherlands

**Keywords:** Activities of daily living, Behavior change, Motor activity, Motor learning, Musculoskeletal pain, Rehabilitation, Self-management

## Abstract

**Objectives:**

This review assessed the effectiveness of combined motor learning (ML) and behavior change (BC) strategies on movement behavior in daily life for patients with chronic musculoskeletal pain (CMP) and analyzed the applied techniques.

**Data sources:**

Literature was searched in PubMed, CINAHL, and Embase databases.

**Study selection:**

The following eligibility criteria were formulated: randomized controlled trials and clinical controlled trials, adults with CMP, movement interventions consisting of ML and BC strategies, and movement behavior or activities in daily life as outcome measures. We used ASReview software for screening.

**Data extraction:**

Two reviewers extracted data and independently assessed the risk of bias of the studies included. A best evidence synthesis was conducted for effectiveness and technique analysis with frameworks for the applied techniques.

**Data synthesis:**

Nineteen papers were selected. Moderate evidence was found in favor of the combined strategies on the quality, and insufficient evidence on the quantity of movement behavior. For activities of daily life, inconsistent evidence was found. The ML techniques reflected a more explicit learning approach. A small portion of BC techniques were applied, such as goal setting and shaping knowledge.

**Conclusions:**

Evidence of the effectiveness of combined strategies is moderate for the quality of movement, insufficient for the quantity of movement, and inconsistent for activities of daily life. The studies used a limited set of ML and BC techniques. Interventions for CMP should apply a more diverse set of techniques and use movement behavior as an outcome measure.

Chronic musculoskeletal pain (CMP) is considered a major problem; it is ranked in the global top 10 for burden of diseases and affects 13.5% to 47% of the general population.^1^^,^^2^ CMP is defined as primary and secondary pain with a duration of more than 3 months.^3^ Because of production loss, work absenteeism, and health care use, CMP leads to excessive costs for society.^1^^,^^4^ Moreover, the patients suffer from the effects of CMP and experience difficulties in their daily activities, social roles, and professional participation.^1^

To solve these problems, patients with CMP receive movement interventions addressing multiple aspects of the International Classification of Functioning, Disability and Health.^5^ This requires a combined approach incorporating various treatment strategies, as recommended by a systematic review of clinical practice guidelines for low back pain.^6^ This review advocates a multimodal intervention for patients with chronic pain, integrating exercise and education based on cognitive behavioral approaches. Ultimately, these multimodal interventions should improve the patients’ movement behavior, which is defined as the observable performance of postures, movement, and activities in daily life in the natural environment of an individual.^7^ All kinds of activities performed by a patient in daily life in any context are included, encompassing self-care, household, occupational, and leisure activities (eg, getting dressed, doing household chores, lifting at work, or cycling for leisure). Ideally, health professionals involved in movement care (physical therapists, exercise therapists, and occupational therapists) should guide patients toward adapting these activities of daily life within their own context. This fits well with the current policy on “the right care in the right place” to offer care in the living environment of patients.^8^ Considering the patients’ context, patients should learn to adapt their movement behavior to that context to function better at the activity or participation level. To describe this behavior, it can be divided into quality of movement and quantity of movement. Quality of movement encompasses fluency, symmetry, or the absence of compensatory movements.^9^^,^^10^ Quantity of movement is described by its volume, intensity, duration, bouts, or counts of movements. Typically, patients with CMP display a reduced quantity of movement compared to healthy individuals.^11^ Also, the quality of movement in these patients is diminished in terms of gait speed, coordination, and variability.^12^, ^13^, ^14^

In a combined approach, a health professional uses a variety of strategies to alter movement behavior*.* The motor learning (ML) strategy typically targets the quality of movement. ML aims at relearning old behavior or mastering new forms of activities of daily life. Kleynen^15^ defined ML as “a set of processes associated with practice or experience leading to relatively permanent changes in capability for producing skilled movement.” Here the health professional trains the patient’s adaptive ability by means of instruction, feedback, and organization of practice.^15^, ^16^, ^17^, ^18^ A second strategy of this approach is behavior change (BC). BC ensures retention and long-term application of the improved movement quality. To patients, BC is a long and difficult process influenced by emotions, cognitions, health beliefs, and social or physical environment.^19^^,^^20^ Health professionals can use a variety of BC techniques (BCTs),^19^ as recommended in clinical practice guidelines for primary care.^21^, ^22^, ^23^, ^24^, ^25^ Not only can BCTs be deployed for movement quality, but they could also aid patients with CMP to address their quantity of movement behavior. Health professionals can use goal setting and self-monitoring to help the patient with planning increased activity throughout the day. There is moderate evidence that BCTs help to increase adherence to physical activity.^26^ However, the chronic character of the complaints of patients with CMP may hinder them from successfully improving their quality or quantity of movement behavior. Therefore, patients with CMP need to adopt self-management skills to cope with the chronicity of their condition. Thus, self-management support (SMS) provided by the health professional is considered essential to the BC strategy in chronic pain interventions.^27^ Patients learn to deal with the limited prospect of pain reduction, their altered social roles, and emotions related to their chronic condition. Typical techniques include maintaining a diary, awareness building, and instructions for home practice.^28^

Ideally, treatment of patients with CMP should include both ML and BC strategies to improve movement behavior in the context. Although health professionals involved in movement care commonly apply these strategies, it remains unclear whether combining them effectively improves the movement behavior of patients with CMP. Furthermore, it remains unclear which techniques are employed by professionals. Therefore, we aimed to assess the effectiveness of movement interventions combining ML and BC strategies to improve the movement behavior of patients with CMP in their daily lives. Additionally, we aim to analyze the specific techniques used by health professionals within the ML and BC strategies.

## Methods

This systematic review was conducted according to the Preferred Reporting Items for Systematic Reviews and Meta-analyses 2020 guidelines.^29^ We published the review protocol in the PROSPERO register: CRD42023389577.

### Eligibility criteria

We built search blocks for inclusion criteria on population, the intervention strategies, outcome measures, and the type of study. For a detailed description, see supplemental appendices S1-S3 (available online only at http://www.archives-pmr.org/).

#### Population

We included trials examining adults aged ≥18 years, suffering from chronic pain of musculoskeletal origin. We used the chronic pain definition of the International Association for the Study of Pain.^3^ Musculoskeletal pain could exist in the trunk and/or extremities. We included compression and overuse syndromes, as well as chronic conditions such as fibromyalgia, osteoporosis, arthritis, and osteoarthritis. Also, spinal deformities such as Scheuermann’s disease and idiopathic scoliosis were included. We excluded trials on participants with previous surgery, infection, malignancy, or pregnancy.

#### Intervention

**Fig. 1:**
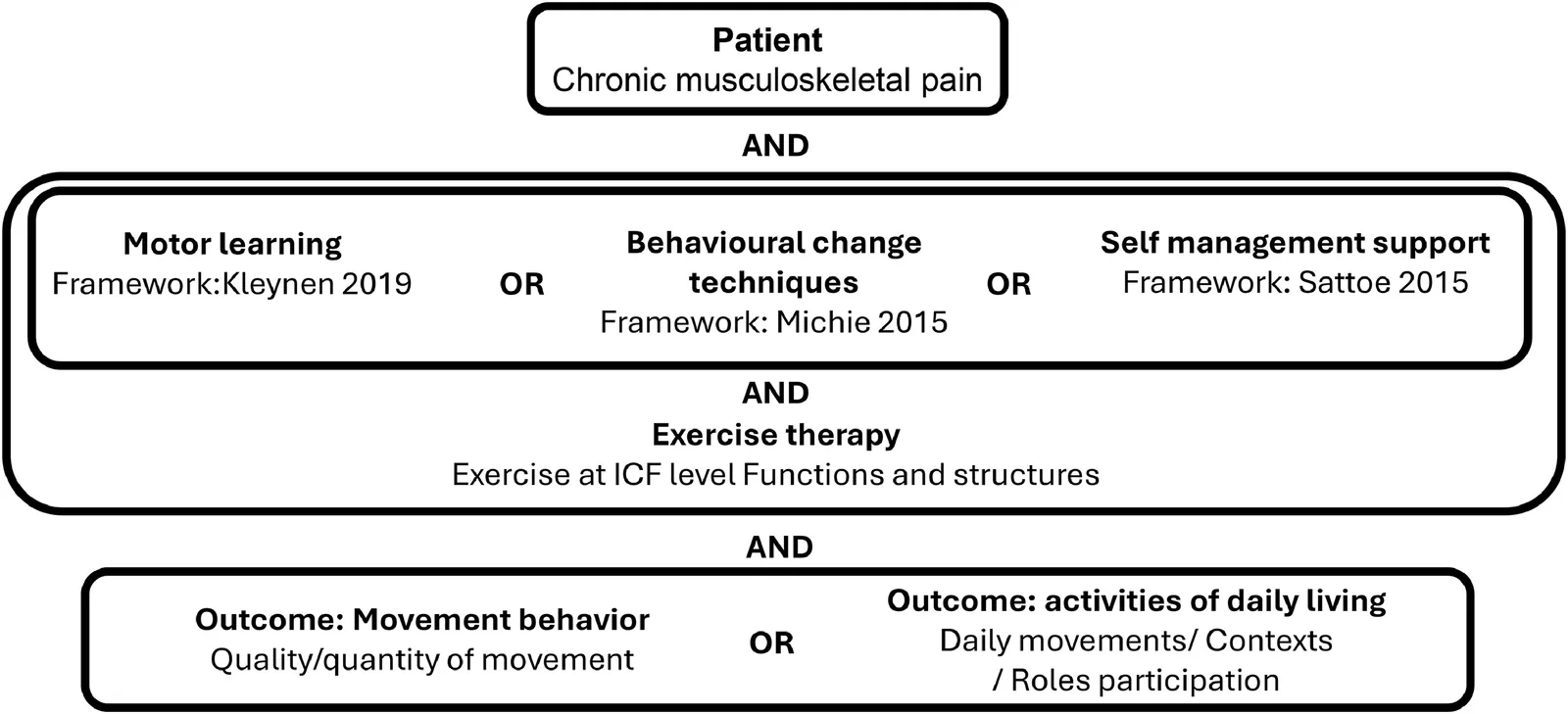
Search strategy: combination of search blocks in the initial search.

Intervention studies provided by health care professionals in primary, secondary, and tertiary care were included. As we aimed to identify interventions that used both BC and ML strategies for patients with CMP, we developed separate search blocks for ML and BC. In addition, we developed a search block for exercise therapy to incorporate exercise-based interventions (fig 1), aiming at the International Classification of Functioning, Disability and Health function level. As such, prerequisites for healthy movement behavior (eg, strength and flexibility) can also be addressed in a combined approach.

#### ML strategy

To describe the ML techniques used by professionals, we used the ML framework of Kleynen.^15^ This framework describes instruction, feedback, and organization. Examples of techniques were observational learning, movement imagery, and dual task learning. Modes of delivery for instruction were implicit or explicit, internal or external focus of attention, and auditory cueing. Feedback could be provided verbally, visually (mirror-use), proprioceptively and could focus on knowledge of results or performance. Also, feedback could be provided during or after execution of the exercise or the daily activity and was also allowed to be self-solicited. Furthermore, trials reporting on organization of environment (complex, open), task (part, whole), or practice (random or blocked) were included.

#### BC strategy

We used the definition for BCTs from the expanded framework for BCTs of Michie et al.^19^ Accordingly, our search strategy incorporated terms corresponding to the overall BCT taxonomy categories and their underlying specific techniques: “goals and planning,” “feedback and monitoring,” “social support,” “shaping knowledge,” “natural consequences,” “repetition and substitution,” “rewards,” “antecedents,” “identity,” and “self-belief.” For a broad search, we added common concepts such as coping, adherence, patient compliance, and relapse. As BC should also encompass SMS, we searched for trials on different modes of delivery of SMS: knowledge education, telemedicine, skill training regarding lifestyle or coping, and motivational interviewing. This was based on the framework of Sattoe et al.^28^ We also added treatment models underpinning both SMS and BC, such as the self-regulation model of health and illness, health belief model, and positive health. As terminology for BC and SMS techniques is often used interchangeably, we combined BC and SMS in one search block.

#### Exercise therapy

For exercise, we searched for terms for strength or flexibility training, be it face to face or remotely delivered by an exercise therapist, physical therapist, or occupational therapist.

#### Control group

Any type of control group was included.

#### Outcomes

The primary outcome for this study was movement behavior, which can be described with its quantity or quality. Quality of movement behavior was addressed with terms such as coordination, balance, timing, symmetry, or fluency.^10^ For quantity, we searched for trials reporting on duration (bouts), intensity (heart rate, metabolic), kinetics, or kinematics (speed, range of motion). As a secondary outcome measure, we included activities in daily life. In our literature search, we looked for daily activities together with the physical context in which they were performed (eg, lifting at work, sitting at home or school, and walking during leisure time).

#### Type of study

We included randomized clinical trials (RCTs) and controlled clinical trials (CCTs) published by peer-reviewed, English-language journals.

### Search strategy

We performed a search in PubMed, CINAHL, and Embase on January 31, 2023. All studies published before this date were eligible for inclusion. We identified RCTs and CCTs on combined interventions of CMP with Medical Subject Headings, and title/abstract search. The syntax for all 3 databases can be found in supplemental appendices S1-S3. Search results from the 3 databases were combined in EndNote 20^a^ and deduplicated with Bramer’s method.^30^ The search strategy is outlined in figure 1 where we combined ML, BC, or SMS with population, exercise therapy, and movement behavior (quality or quantity) or activities of daily life (type and context).

### Study selection

As the search resulted in too many hits to screen manually, we performed an AI-supported relevance ranking to identify trials for full-text screening. ASReview^31^^,^^32^ was trained with prior knowledge*.* Ten trials meeting all inclusion criteria across a range of diagnoses, intervention types, and outcome measures were introduced to the AI algorithm. In addition, 10 trials were included that did not fully meet the inclusion criteria because of differences in study design, nonchronic diagnoses, different outcome measures, or the absence of combined strategies. These 20 studies were selected by M.T. and I.v.D. in a consensus meeting and checked by a third researcher (D.B.*)*. After calibration of the 2 reviewers (I.v.D. and M.T.) in 3 rounds (kappa=0.48; kappa=0.51; kappa=1.00) on 150 randomly selected articles from the initial search, they alternately selected relevant trials within ASReview, based on anonymized title and abstract. Screening ended when one of the stopping criteria (10% of total hits or 100 consecutive irrelevant hits) was met.^33^ To ensure that both ML and BC (including SMS) were described, we conducted an independent second selection round after using ASReview. During this second round, and following a consensus meeting (I.v.D. and M.T.), we included only papers that reported both strategies in their interventions. We used EndNote 20 to manage references and original publications.

### Data collection

Two reviewers extracted data with a standardized form. This included details for participant description (diagnosis, age, %women, duration of chronic pain, sample size), design, control group, outcome measure, results, and direction of effect.

### Risk of bias assessment

Two reviewers (I.v.D. and M.T.) independently evaluated the methodological quality of both RCTs and CCTs using the revised Cochrane risk of bias (RoB) tool 2.^34^^,c^ They assessed bias arising from 5 domains (selection, performance, detection, attrition, and reporting). We assigned an overall RoB judgment, based on all domains (“Low risk,” “Some concerns,” or “High risk”).

### Synthesis

A best evidence synthesis was conducted to summarize the effectiveness of the combined strategies for movement behavior and activities of daily life. This synthesis included the consistency of results at endpoint, the methodological quality, and the number of studies. The methodology was based on van Tulder et al,^35^ which was adapted by Proper et al,^36^ and Kloek et al.^37^, ^38^, ^39^ For all outcome measures, the level of evidence was determined as strong, moderate, inconsistent, or insufficient. Results were defined as consistent when ≥75% of the studies reported results in the same direction.

### Technique analysis

For both strategies, we conducted a thorough analysis of the specific techniques used by health care professionals in the interventions, which aimed to change patients’ movement behavior in daily life. Two reviewers (I.v.D. and M.T.) independently and closely read the full texts plus appendices when available and identified, as comprehensively as possible, the specific techniques described in each intervention. Although some techniques are performed by patients, we classified them as therapist-provided techniques because they are initiated, instructed, and supervised by the therapist. Identified techniques were finalized in a consensus meeting*.* We described the techniques used within the intervention according to the frameworks for ML^15^ and BC.^19^^,^^28^ Furthermore, we added the level of tailoring, the number of sessions, duration, and the mode of delivery. We reported the underpinning model of the intervention when mentioned.

## Results

### Study selection

**Fig. 2:**
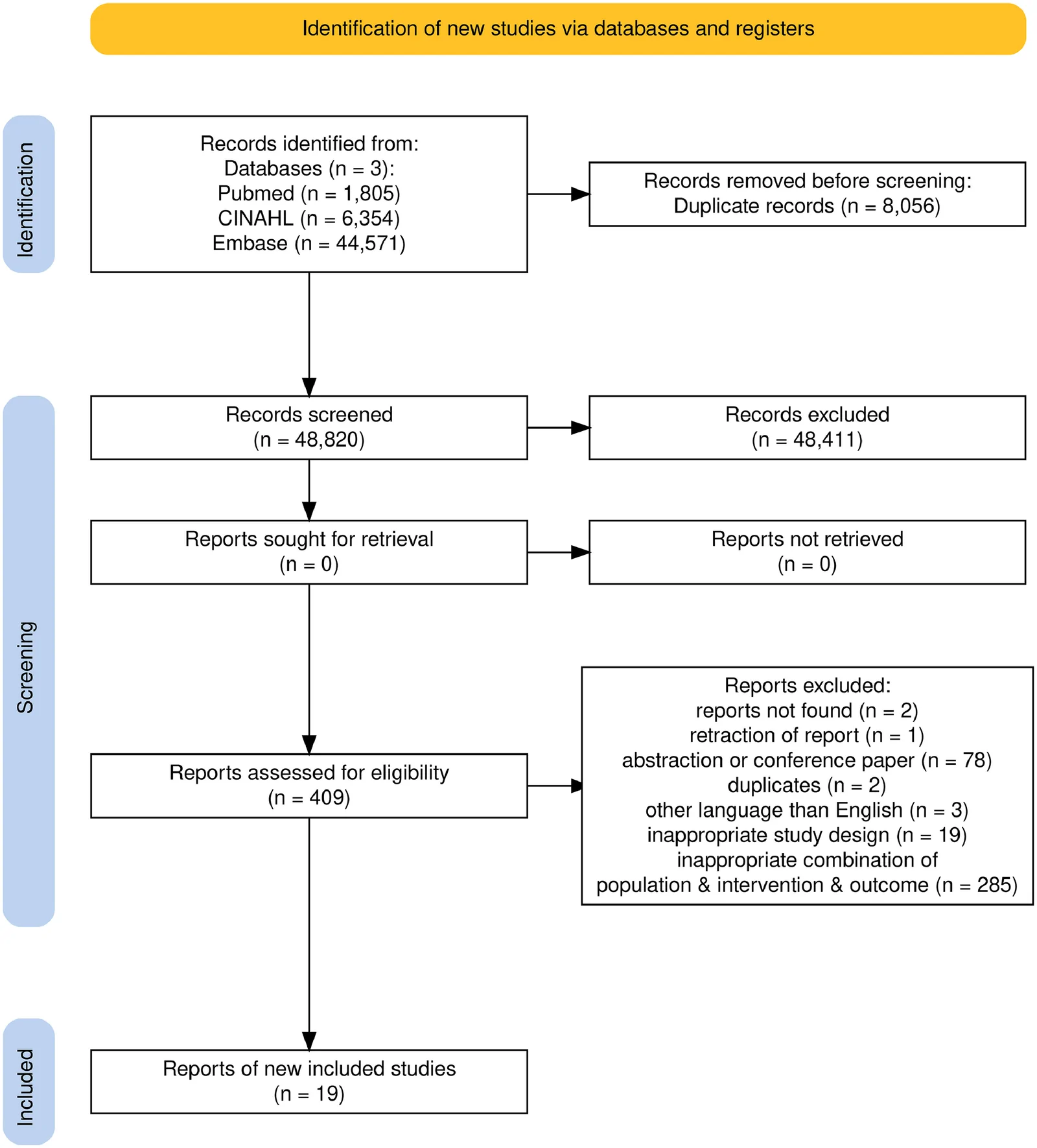
Flowchart of study selection process.

The search of PubMed, CINAHL, and Embase (January 2023) resulted in 56,876 papers. The flowchart (fig 2) shows the search and selection procedure. After removing duplicates, 48,820 papers were screened using ASReview. After screening 5.7% of the identified papers, we met the stopping rule of 100 consecutive irrelevant hits. As this was sooner than expected, we screened 50 papers more, which were also all irrelevant. This resulted in 409 potentially relevant papers.

In the second round of screening, studies reporting on both ML and BC were included. We allowed an OR between the BC and SMS, as these were often used interchangeably in the literature. This resulted in 19 papers published between 1996 and 2022, reporting on 16 RCTs or CCTs. Reasons for exclusion were paper not found (n=2), paper retracted (n=1), abstract or conference paper (n=78), duplicate (n=2), other language than English (n=3), inappropriate study design (n=19), and the combination of population, intervention, and outcome measures not met (n=285).

### Study description

The 16 studies were primarily conducted in Europe, with 11 studies taking place in Scandinavia. The interventions were heterogeneous, ranging from occupational therapy*,* Mensendieck therapy to cognitive behavioral approaches. The setting of the therapy was mostly primary care, while in 5 studies, patients received multidisciplinary care in a rehabilitation center. Also, the content of the care given to the control group varied substantially. Incorporated trials were RCTs for all but 2. Three of the RCTs were block randomized. Patients mostly suffered from low back pain and fibromyalgia. The average age of all participants was 46 years. Participants experienced pain and/or disabilities for 65 months on average, and 70% of participants were women. For a detailed description, see table 1.

**Table 1:** Characteristics of selected studies: study, participants, outcome measures, and results.

		Participants		Outcome		Results	Direction of Effect I >, =, < C
Study	General Description of Trial	Diagnosis	Sample Size	Movement Behavior	Activities of Daily Life / Context		
First author, Country	Intervention: Control: Design:	Diagnosis Age, mean (SD), %females Duration, mean (SD)	Number of participants Intervention: n (endpoint), n dropout (%), n lost to follow up (%) Control: n(endpoint), n dropout (%), n lost to follow up (%)	Quality of movement: coordination, balance, timing, symmetry, fluency. Instrument (questionnaire, performance test, observational instrument, etc.): Quantity of movement: bouts, intensity, ROM etc. Instrument:	Outcome measure Instrument (questionnaire, performance test, observational instrument, etc.): Activity (movement, posture) Context (work, sport, leisure, home)	Instrument, timepoints Difference in change, CI, *P* value	
Haugstad et al^40^ Norway	Intervention: Body awareness and cognitive approach, Mensendieck therapy (leading the patient to enhanced body awareness through an active use of new cognitive patterns and using this new awareness in the activities of daily life) Control: Standard gynecologic advice Design: RCT	Diagnosis: Chronic pelvic pain unexplained by pelvic pathology Age: 34 y (SD 2), %females: 100 Duration: 6 y (SD 0.8)	Randomized: n=40 Intervention: n=19, dropout: n=2 (10%) Control: n=18, dropout: n=1 (5%)	Quality of movement Instrument: Standardized Mensendieck test (SMT) performance scores of optimal functions for posture(standing, sitting), movement, gait, sitting posture, and respiration.		SMT scores at 3 mo: average ± SEM (%change after 3 mo, %change after 12 mo) Control: within all *P*=NS at 3 mo and at 12 mo: Posture 4.15 ± 0.14 (+0.2% to −2%) Movement 3.52 ± 0.20 (−4.6% to −2%) Gait 3.45 ± 0.22 (−1.7% to −3,4%) Sitting posture 3.74 ± 0.21 (+1.4%, +2,4%) Respiration 3.35 ± 0.22 (−5.1%, −3,7%) Intervention: within all *P*<.01 at 3 mo: Posture 4.37 ± 0.38 (+19.3%-+27,3%) Movement 4.13 ± 0.38 (+26.1%-+42,2%) Gait 4.13 ± 0.39 (+24.8%-+37,6%) Sitting posture 4.67 ± 0.36 (+27.9%-+37,2%) Respiration 4.72 ± 0.37 (+58.4%-+79,5%) 2006, 2008 Statistics only within not between	**SMT:** **I>C**
Asenlof et al ^41^ Sweden	Intervention: Cognitive behavioral medicine intervention consisting of mixed exercise therapy, motor learning and behavioral change techniques. Control: General physical exercise therapy aimed at enhancing strength, endurance, balance, stability, range of motion, and aerobic capacity Design: RCT	Diagnosis: Persistent musculoskeletal pain Intervention: Age: 41.6 y (SD 12.4), %females: 82 Duration: 2.6 y Control: Age: 43.4 y (SD 10.9), %females: 73 Duration: 2.0 y	Randomized n=122 Intervention: n=43, dropout n=22 (34%) Control: n=38, dropout n=19 (33%)		Outcome measure: Pain-related disability Instrument: Pain Disability Index (PDI) (7 categories NRS: family and home responsibilities, recreation, social activity, occupation and education, sexual behavior, self-care, life-support activity)	PDI, group: Baseline, Post treatment, 3-mo follow up; score (SD) PDI, Intervention: 23.4 (11.6), 7.1(7.9), 7.7(8.4) PDI, Control: 27.1 (14.1),18.8 (14,7), 16.4(13.2) Within group (Time): CI(differences) 10.2-16.3 *P*=.001 Between group (Condition): CI(differences) −12.4 to −3.6, *P*=.001 Univariate analysis: Significant time x condition interaction F=4.88, *P*=.01	**PDI:** **I>C**
Dragesund et al^42^ Norway	Intervention: Cognitive Patient Education combined with active individualized physiotherapy Control: Norwegian Psychomotor Physiotherapy (hands-on body awareness physical therapy focusing on mind-body connection, breathing patterns, stress/muscle tone interaction) Design: Block randomized RCT	Diagnosis: Long lasting widespread musculoskeletal pain and/or pain located to the neck and shoulders, fibromyalgia tender points Intervention: Age: 46 y (12), %females 92 Duration: not quantified, long lasting Control: Age: 43 y (11), %females 95 Duration: not quantified, longlisting	Randomized: n=128 Intervention: n=38, dropout=18, lost to follow up: 8 Control: n=48, dropout=9, lost to follow up: 7		Outcome measure: Disability Instrument: Norwegian Function Assessment Scale (NFAS): 39 items in 7 disability domains: walking/ standing, holding/picking up something, lifting/carrying, sitting, coping/ managing, cooperation/communication, and senses. Outcome measure: Disability Instrument: Neck Disability Index (NDI), measures disability because of neck pain, 10 items Outcome measure: Pain and Disability Instrument: Shoulder Pain and Disability (SPADI) measures shoulder pain and function (5 pain items, 8 function item)	NFAS: t0 vs t1 Between: estimated mean difference −0.13(CI, 0.27-0.02) *P*=.10 NFAS: t0 vs t2 Between: estimated mean difference −0.08(CI, 0.26-0.08) *P*=.58 Intervention: Effect size within: Cohen’s *d*=0.23 Control: Effect size within: Cohen’s *d*=0.42 Intention to treat analysis: Time x Group interaction *P*=.10 t1, t,2: NDI, SPADI not reported, only used as severity check for inclusion	**NFAS:** **I=C**
Amris et al^43^, ^44^ Denmark	Intervention: Occupational therapy intervention program plus standardized group-based interdisciplinary rehabilitation program Control: Physiotherapy intervention program plus standardized group-based interdisciplinary rehabilitation program Design: RCT, active-controlled nonblinded	Diagnosis: chronic widespread pain according to the American College of Rheumatology (ACR) (fibromyalgia) Intervention: Age: 45 y (SD 11), %females: 100 Duration 9.5 y (IQR 5-15) Control: Age 44 y (SD 10), %females: 100 Duration: 10 y (IQR 5-15)	Randomized: n=191 (all arms of larger study) Intervention (arm within larger study): n=25; dropout: n=18 (42%); lost to follow up: unclear Control (arm within larger study): n=30; dropout: n=12 (29%); lost to follow up: unclear	Quality of movement: ADL ability. Instrument: Assessment of Motor and Process Skills (AMPS); ADL motor ability (amount of effort, fatigue and/or clumsiness). AMPS also addresses ADL process ability (degree of disorganization, inappropriate use of time, space or objects and adapting actions). The quality of 16 ADL motor skills (move self and objects) and 20 ADL process skills (organize and adapt actions) is scored on a 4-point ordinal scale in terms of ease, efficiency, safety, and independence.	Outcome measure: functioning Instrument: subscales of SF-36: physical function; scale is expressed with values 0-100, where low scores indicate poor health Instrument: a general questionnaire	Group: AMPS mean changes for ADL ability at 88 wk from baseline, 95% CI Motor ability Intervention: −0.006, −0.244 to 0.233 Control: −0.045, −0.291 to 0.202 Between group difference: *P*=.903 Group: SF-36 physical function mean changes at 88 wk from baseline, 95% CI Intervention: 7.68, −2.08 to 17.44 Control: 4.92 to −5.29 to 15.14 Between group difference: *P*=.484	**AMPS** **motor ability:** **I=C** **SF-36 physical function:** **I=C**
Olsen et al^45^ Norway	Intervention: Basic Body Awareness Therapy preceded by patient education Control: Patient education Design: RCT	Diagnosis: Primary hip osteoarthritis (American College of Rheumatology Clinical Criteria) Intervention: Age: 64.8 y (range 44-83), %females: 78 Duration: not reported Control: Age: 61 y (range 23-78), %females: 80 Duration: not reported	Randomized: n=101 Intervention: n=41, drop out + lost to follow up: n=10 (20%) Control: n=45, drop out + lost to follow up: n=5 (10%)	Quality of movement: Instrument: Body Awareness Rating Scale-Movement Quality and Experience (BARS‐MQE) Quantity of movement: Self-reported activity score UCLA	Function in daily life (Hip Osteoarthritis Outcome Score (HOOS), A-subscale activities of daily life, SP-subscale sport and leisure)	Instrument, Group: Mean changes (SD), *P* value paired *t* test Quality of movement **BARS-MQE**: Intervention: 5.06 (5.96) **<.001**; Control: 0.48 (3.92) 0.424 ANCOVA: *d*=0.849, *P* <.001 Quantity of movement **UCLA**: Intervention: 0.07 (1.68) 0.782; Control 0.22 (2.00) 0.460 ANCOVA: *d*=0.08, *P*=.33 Activities of daily life **HOOS-A**, Intervention: 1.45 (13.27) 0.487; Control: 2.39 (12.57) 0.210 ANCOVA: *d*=−0.07, *P*=.783 **HOOS-SP**,Intervention:0.90 (17.77) 0.748; Control: −0.69 (16.77) 0.784 ANCOVA: *d*=0.090, *P*=.788	**BARS-MQE:** **I>C** **UCLA:** **I=C** **HOOS-A:** **I=C** **HOOS-SP** **I=C**
In et al^46^ Republic of Korea	Intervention: Multidimensional Treatment Control: Unimodal Treatment Design: RCT	Diagnosis: Nonspecific low back pain for 3 mo or longer Intervention: Age: 41.1 y (SD 11.5), %females: 33 Duration: 11mo (4,2) Control: Age: 40.6 y (SD 11.3), %females: 40 Duration: 13mo (6,0)	Randomized: n=60 Intervention: n=30, drop out + lost to follow up: n=0 Control: n=30, drop out + lost to follow up: n=0	Quality of movement: Thoracolumbar kyphosis (TK) and lumbar lordosis (LL) were measured during sitting and recorded using a motion capture system	Functional disability arising from LBP (Oswestry Disability Index (ODI)	Group within: t0 score (SD) vs t1 score (SD), *P* value paired *t* test Quality of movement **TK**: Intervention within: 9.68±10.51 vs 5.54±6.89, *P*<.001 Control within :9.14±8.76 vs 8.31±8.17, *P*<.001 Between: t −3.011, *P*<.005 Significant interaction between intervention and time (F (1, 58)**=**9.434, *P*=.003) **LL**: Intervention within: 5.01±9.85 vs 0.05±7.02, *P*<.001, Control within: 4.85±9.51 vs 3.04±9.47, *P*<.001 Between: t**=**−2.883, *P*<.005 Significant interaction between intervention and time (F (1,58)**=**8.270, *P*=.006 Activities of daily life **ODI**: Intervention within: 38.5±3.8, 28.1±4.5, *P*<.001 Control within: 37.8±4.1, 31.8±3.4, *P*<.001 Between: t**=**−5.304, *P*=.001 Significant interaction between intervention and time (F (1, 58) =17.191, *P*<.001)	**TK:** **I>C** **LL:** **I>C** **ODI:** **I>C**
Soukup et al^47^ Norway	Intervention: Mensendieck group program Control: Information on Mensendieck program but no participation	Diagnosis: Recurrent low back pain Intervention: Age: median 41 y, %females: 52 Duration: not stated Control group: Age: median 40 y, %females: 49 Duration: not stated	Randomized: n=77 Intervention: n=34, dropout: n=5 (13%), lost to follow up: n=3 (9%) Control group: n=35, dropout: n=3 (8%), lost to follow up: n=0 (0%)		General functional status (Dartmouth COOP Functional Status Assessment Charts)	Intervention: COOP, 16.3(±5.2), 14.5(±5.6), 12.9(3.7) at 0, 12, 36 mo Control: COOP, 16.1(±4.9), 15.3(±5.6), 14.5(±4.4) at 0, 12, 36 mo ANOVA *P*=.09	**COOP:** **I=C**
Bendix et al^48^ Denmark	Intervention: Multidisciplinary intensive treatment Control: No treatment Design: RCT, prospective study	Diagnosis: Low back pain >6 mo Intervention: Age: median 41 y, %females: 78 Duration: not stated Control group: Age: median 40 y, %females: 69 Duration: not stated	Randomized: n=106 Intervention: n=45, dropout: n=9 (16%), lost to follow up: n=1 (2%) Control group: n=49, dropout: n=0 (0%), lost to follow up: n=2 (5%)		Functional ability (back interference with activities of daily life questionnaire)	Intervention: Function score 12.1 (IQR 7.2-16.8) Control: Function score 16.8 (IQR 13.1-20.1) Significantly lower interference in treated group at follow up when compared with control group *P*<.001	**Functional** **ability:** **I>C**
Areso-Boveda et al^49^ Spain	Intervention: Pain neuroscience education and exercise Control: Treatment as usual Design: nonrandomized clinical trial	Diagnosis: Fibromyalgia Intervention: Age: 57.4 y (SD 9.1), %females: 100 Duration: 8.4 y (SD 8.5) Control: Age: 64.1 y (SD 9.0), %females: 100 Duration: 10.1 y (SD 8.7)	Randomized: n=64 Intervention: n=35; dropout: n=1 (1.9%), lost to follow up: n=10 (22%) Control: n=18; dropout: n=0, lost to follow up: n=1 (5%)		Outcome measure: impact of pain in the patient’s daily life Instrument: subscale impact of life of Brief Pain Inventory - short form (a-BPI-sf): a general questionnaire to assess influence of pain in different aspects of life during the previous wk (total score: 0-70). Outcome measure: functional capacity (activities) Instrument: Health Assessment Questionnaire: HAQ: a general questionnaire that evaluates the difficulty in performing different activities of daily life (score 0-3)	T1: 1 y after intervention **a-BPI-sf** T1: difference: −13.6, 95% CI: −22.9 to −4.2, *P*<.05 **HAQ** T1: difference: −0.5, 95% CI: −0.9 to −0.1), *P*<.05	**a-BPI-sf:** **I>C** **HAQ:** **I>C**
Kjeken et al^50^ Norway	Intervention: Multidisciplinary inpatient rehabilitation program Control: Treatment as usual Design: RCT	Diagnosis: Ankylosing spondylitis Intervention: Age: 49.4 y (SD 10.3), %females: 21.7 Duration: 14.9 y (SD 9.6) Control: Age: 48.6 y (SD 9.8), %females: 46.9 Duration: 16.1 y (SD 12.0)	Randomized: n=100 Intervention: n=29, dropout 4 mo: n=14 (27%), lost to follow up: n=22 (43%) Control: n=34, dropout 4 mo: n=14 (29%), lost to follow up: n=15 (31%)		Outcome measure: disabilities Instrument: Bath Ankylosing Spondylitis Functional Index (BASFI): a disease specific questionnaire about disabilities of daily activities and paid work (mean score of 10 questions assessed on VAS scales (0**=**good functioning) Outcome measure: functioning Instrument: subscales of SF-36: physical functioning, role limitations because of physical problems, social functioning; each scale is expressed with values 0-100, where low scores indicate poor health Instrument: a general questionnaire Outcome measure: activity limitations and participation restrictions Instrument: Canadian Occupational Performance Measure (COPM) to describe and measure patients perception of activity performance and satisfaction (at baseline, not used at 4 mo and 1 y)	T1: 4 mo: T2: 1 y. **BASF**I T1: mean difference: −6.0, 95% CI: −13.1 to 1.1, *P*=.10 BASFI T2: mean difference: −0.7, 95% CI: −8.2 to 6.8, *P*=.86 **SF-36 physical function** T1: mean difference: 2.2, 95% CI: −4.6 to 9.0, *P* not reported SF-36 physical function T2: mean difference: 2.0, 95% CI: −5.3 to 9.3, *P* not reported **SF-36 social functioning** T1: mean difference: 7.8, 95% CI: −1.8 to 17.4, *P*=.11 SF-36 social functioning T2: mean difference: 6.0, 95% CI: −4.8 to 16.3, *P*=.25 **SF-36 role physica**l T1: mean difference: 11.9, 95% CI: 1.8, to 21.9), *P*=.02, significant SF-36 role physical T2: mean difference: 3.0, 95% CI: −7.8 to 13.8), *P*=.59 In all: improvement was greater in intervention	**BASFI:** **I=C** **SF-36 physical function:** **I=C** **SF-36 social functioning:** **I=C** **SF-36 role physical 4** mo**:** **I>C** **SF-36 role physical 12** mo**:** **I=C**
Monticone et al^51^ Italy	Intervention: Motor and cognitive rehabilitation Control: General physiotherapy Design: RCT	Diagnosis: Idiopathic scoliosis Intervention: Age: 51.6 y (SD 8.1), %females: 74 Duration: 37.9mo (SD 20.5) Control: Age: 51.7 y (SD 8.5), %females: 71 Duration: 35.4mo (SD 19.9)	Randomized: n=130 Intervention: n=57, dropout: n=4 (6.2%), lost to follow up: n=8 (12.3%) Control: n**=**55, dropout: n=5 (7.7%), lost to follow up: n=10 (15%)		Outcome measure: disabilities Instrument: (Italian) Oswestry Disability Index (ODI), a self-administered 10-item questionnaire to evaluate the intensity of pain and its disabling effect on daily activities (0 (no disability) - 100 (maximum disability)), a disease specific questionnaire for low back pain	T1: 20 wk (post-training), T2: follow up at 12 mo ODI T1: difference: −12.0, 95% CI: −14.3 to −9.8), *P*<.001 ODI T2: difference: −14.8, 95% CI: −17.1 to −12.6), *P*<.001	**ODI:** **I>C**
Dufour et al^52^ Denmark	Intervention: Multidisciplinary biopsychosocial rehabilitation combined exercise, education and pain management Control: Back muscle strengthening exercises Design: RCT	Diagnosis: Chronic low back pain Intervention: Age: 41.2 y (SD 10.0), %females: 56.6 Duration: 1.5 y (SD 2.3) Control: Age: 40.6 y (SD 9.1); %females: 55.9 Duration: 1.5 y (SD 2.2)	Randomized: n=286; Intervention: n=83, dropout: n=24 (17%), lost to follow up 6 mo: n=25 (18%), 12 mo: n=40 (28%), 24 mo: n=49 (35%) Control: n=91; dropout: n=12 (8.3%), lost to follow up 6 mo: n=20 (14%), 12 mo: n=35 (24%), 24 mo: n= 51 (35%)		Outcome measure: disabilities Instrument: Roland-Morris Disability Questionnaire (RMDQ), a lower back specific questionnaire Outcome measure: disabilities Instrument: Subscales physical functioning, role limitations because of physical problems, social functioning of short form general health survey scale (SF-36). A general questionnaire	T1: 3 mo (end treatment), T2: 6 mo, T3: 12 mo, T4: 24 mo. **RMDQ** T1 improvement: Intervention: 3.0 (5.3), control: 1.5 (4.4); *P*=.003 RMDQ T3 improvement: Intervention: 3.3 (5.7), control: 1.2 (5.1) Intervention achieved a significantly greater improvement (*P*=.003) than that attained by control . This difference was maintained throughout the follow up. **SF-36 physical functioning** T1 improvement: Intervention: 12.2 (21.2), control: 6.0 (17.7); SF-36 physical functioning T3 improvement: Intervention: 12.1 (24.0), control: 2.0 (19.0); Patients in both groups improved significantly. The improvement was significantly greater (0.000) in intervention than in control at T1 and follow up. **SF-36 role limitation physica**l T1 improvement: Intervention: 16.7 (39.6), control: 13.5 (35.3); SF-36 role limitation physical T3 improvement: Intervention: 25.2 (40.8), control: 16.9 (38.4); Patients in both groups improved significantly. The improvement was not significantly greater (*P*=.221) in intervention than in control at T1 and follow up. **SF-36 social functioning** T1 improvement: Intervention: 7.7 (25.4), control: 7.3 (20.1); SF-36 social functioning T3 improvement: Intervention: 8.6 (28.3), control: 4.2 (25.0); Patients in both groups improved significantly. The improvement was not significantly (*P*=.578) greater in intervention than in control at T1 and follow up	**RMDQ:** **I>C** **SF-36 physical functioning:** **I>C** **SF-36 role limitation physical:** **I=C** **SF-36 social functioning:** **I=C**
George et al^53^ United States of America (Florida)	Intervention: Multidisciplinary rehabilitation with graded exposure. Graded exposure: progressing specific feared activities with a hierarchical exposure paradigm. Control: Multidisciplinary rehabilitation with graded exercise. Graded exercise: progressing activities with a quota-based system. Design: CCT	Diagnosis: Chronic low back pain Intervention: Age: 44.8y (SD 9.1), %females: 44 Duration: not reported Control: Age: 47.1 y (SD 11.9), %females: 60 Duration: not reported	Randomized: n=33 Intervention: n**=**not clearly stated; dropout: not clearly stated Control: n**=**not clearly stated; dropout: not clearly stated		Outcome measure: disabilities Instrument: Modified Oswestry Disability Questionnaire (ODQ), a disease specific self-report questionnaire to evaluate how much low back pain is limiting activities of daily living (like sitting, standing, walking, and lifting) (range: 0-100; higher number indicating greater disability)	T1: 4 wk Graded exposure: pre: 43.3 ± 12.5; post: 38.0 ± 14.1; *P*<.01 Graded exercise: pre: 56.0 ± 13.9, post: 47.0 ± 17.1; *P*<.01 Both groups showed significant reduction in ODQ following treatment There is no difference in groups (*P*=.44)	**ODQ:** **I=C**
Taimela et al^54^ Finland	Intervention: Multimodal treatment with proprioceptive exercises, relaxation and behavioral support Control1: Training for home exercises Control2: Recommendation to exercises Design: RCT	Diagnosis: Chronic neck pain Intervention: Age: woman: 44.0 y (SD 8.4), men: 38.8 y (SD 7.6), %females: 68 Control1: Age: woman: 44.8 y (SD 9.0), men: 36.0 y (SD 8.0), %female: 76 Control 2: Age: woman: 47.1 y (SD 16.8), men: 43.2 y (SD 11,0), %female: 69 Duration overall: 7.6 y (SD 6.0)	Randomized: n=76; Intervention: n=21, dropout: n=3 (12%), lost to follow up: n=4 (16%) Control1: n=18, dropout: n=5 (20%), lost to follow up: n=7 (28%) Control2: n=22, dropout: n=3 (12%), lost to follow up: n=4 (15%)		Outcome measure: disability Instrument: Questionnaire, a series of 13 questions on activities in daily living, each assessed with a 100-mm VAS scale (average) Outcome measure: working ability Instrument: questions of a questionnaire asking for working ability and absenteeism (1 (I strongly disagree - 5, I strongly agree)	T1: 3 mo, T2: 12 mo **Disability** T1: scores not reported Disability T2: scores not reported No statistically discernible differences were noted among the groups in the reduction of disability (F =0.27, *P*=.73) **Working ability** T1: score not reported; An improvement in favor of the ACTIVE treatment was seen at 3 mo (ANOVA H**=**1 1 .0, *P*=.004) Working ability T2: score not reported; An improvement in favor of the ACTIVE treatment was seen at 12-mon follow up (H**=**9.3, *P*=.01)	**Disability:** **I=C** **Working ability:** **I>C**
Vibe Fersum et al,^55^ Vibe Fersum et al^56^ (follow up) Norway	Intervention: Cognitive functional therapy Control: Manual therapy and exercise Design: RCT	Chronic low back pain Intervention: Age: 41.0 y (SD 10.3), %females: 52.9 Duration (n): 3 -12 mo: 6; 1-5 y: 14; >5 y: 31 Control: Age: 42.9 y (SD 12.5), %females: 48.8 Duration (n): 3 -12 mo: 6; 1-5 y: 13; >5 y: 23	Randomized: n=112 Intervention: n=51, dropout/lost to follow up: n=10 (16%) Control: n=43, dropout: n=8 (16%)		Outcome measure: disabilities Instrument: Oswestry Disability Index (ODI), a disease specific questionnaire for low back pain	T1: 3 mo, T2: 12 mo ODI T1: mean difference: −9.7, 95% CI: −12.7 to −6.7), *P*<.001 ODI T2: mean difference: −8.2, 95% CI: −12.6 to −3.8), *P*<.001	**ODI:** **I>C**
Hammond and Freeman ^57^ United Kingdom	Intervention: educational-behavioral joint protection program Control: standard arthritis education Design: RCT, block randomization	Diagnosis: rheumatoid arthritis Intervention: Age: 49.5 y (SD 11.4), %females: 81.5 Duration: 17.5 mo (SD 14.8) Control: Age: 51.6 y (SD 9.7), %females: 71 Duration: 21.4 mo (SD 18.7)	Randomized: n=139 Intervention: n=63, dropout: n=7 (9.7%), follow up: n=2 (2.7%) Control: n=60, dropout: n=5 (8.1%), follow up: n=2 (3.0%)	Quality of movement: Joint Protection Behavior Assessment; observational measure to evaluate joint protection methods during arm-activities	Outcome measure: functional assessment Instrument: The AIMS2 (Arthritis Impact Measurement Scales), a disease specific questionnaire for arthritis	T1: 6 mo, T2: 12 mo **JPBA** T1 Intervention: T0: 16.16 (12.48), T1: 35.54 (20.05); significant improvement (*P*<.01) Control: T0: (14.97 (12.91), T1: 18.00; No differences between the groups at 6 mo. JPBA T2 Intervention: T0: 16.16 (12.48), T2: 30.72 (18.75); significant improvement (*P*<.01) Control: T0: (14.97 (12.91), T2: 17.88; (15.09), There is a significant difference in groups (*P*=.001) at 12 mo. **AIMS2** ADL T1 Intervention: T0: 1.62 (2.01), T1: 1.43 (1.80) Control: T0: 2.13 (2.40), T1: 1.90 (2.18); No differences between the groups at 6 mo. AIMS2 ADL T2 Intervention: T0: 1.62 (2.01), T2: 1.33 (1.82) Control: T0: 2.13 (2.40), T2: 2.13 (2.46) There is a significant difference in groups (*P*=.04) at 12 mo	**JPBA 6** mo **I=C** **JPBA 12** mo **I>C** **AIMS-ADL 6** mo **I=C** **AIMS-ADL 12** mo **I>C**

*Abbreviations:* CCT, controlled clinical trial; I<C, intervention significantly worse than control; I=C, intervention not significantly better than control; I>C, intervention significantly better than control; IQR, interquartile range; n, number; NS, not significant; RCT, randomized clinical trial.

### RoB assessment

We assessed 21 reported outcome measures from the 16 studies (table 2). The overall RoB was high for 16 outcome measures, whereas 5 raised some concern. Randomization was generally well performed and reported: 16 RoBs revealed low risk of bias. Because of inaccuracies in statistical analysis or description, 16 RoBs had some concern or high risk for deviations from intended interventions. High dropout rates contributed to bias of missing outcome data, with 10 RoBs having a high risk of bias or some concern, and 11 RoBs low risk of bias. Based on knowledge of the interventions and unblinded assessors on questionnaires, 16 RoBs were rated as high risk or some concern for bias in measurement of the outcome. Bias in the selection of reported outcome measures was assessed as low risk in 6 RoBs, and as some concern in 15, these often lacked descriptions of a prespecified analysis plan.

**Table 2:** Risk of bias assessment for outcome measures of included studies.

Abbreviations: , Some concerns; , high risk; , low risk; D1, randomization process; D2, deviations from the intended interventions; D3, missing outcome data; D4, measurement of outcome; D5, selection of the reported result.

### Synthesis

#### Movement behavior

A total of 5 studies employed quality of movement as an outcome measure, of which 3 were assessed as having a high risk of bias and 2 some concern (table 3). Four of the 5 studies showed significant differences between the intervention and control groups. These control groups included information, exercises, and joint protection skills^57^; a seminar focused on physical activity^45^; standard gynecologic advice^40^; and unsupervised exercises.^46^ In conclusion, moderate evidence was found in favor of the combined strategies on the quality of movement behavior.

**Table 3:** Best evidence synthesis: outcome measures, studies, direction of effect, risk of bias, and level of evidence.

General Outcome Measure	Construct	Study, First Author (Measurement Instrument)	Direction of Effect	Risk of Bias
Quality of Movement Behavior				
	Performance score of optimal function	Haugstad (SMT)	I>C	Some
	ADL Motor ability	Amris (AMPS motor ability)	I=C	High
	Movement quality patient experience	Olsen (BARS-MQE)	I>C	High
	Sitting lumbar lordosis and thoracic kyphosis (ROM)	In (motion capture)	I>C	Some
	Observation Joint Protection Behavior	Hammond (JPBA)	I>C	High
Quantity of movement behavior				
	Self-reported activity score	Olsen (UCLA)	I=C	High
Activities of daily life				
	Disability			
	Pain-related disability	Asenlof (PDI)	I>C	High
	Disability	Dragesund (NFAS)	I=C	High
	Functioning: physical	Amris (SF-36 physical function)	I=C	High
	Function in activities of daily life	Olsen (HOOS-A)	I=C	High
	Function in sport	Olsen (HOOS-SP)	I=C	High
	Functional disability	In (ODI)	I>C	Some
	General functional status	Soukup (COOP)	I=C	High
	Functional ability	Bendix (Function Score)	I>C	High
	Pain impact on daily life	Areso-Boveda (a-BDP-sf)	I>C	High
	Functional capacity for activities	Areso-Boveda (HAQ)	I>C	High
	Disabilities of daily activities and work	Kjeken (BASFI)	I=C	Some
	Functioning: physical	Kjeken (SF-36 physical)	I=C	Some
	Functioning: social	Kjeken (SF-36 social)	I=C	Some
	Disabilities of daily activities	Monticone (ODI)	I>C	Some
	Disabilities	Dufour (RMDQ)	I>C	High
	Functioning: physical	Dufour (SF-36 physical)	I>C	High
	Functioning: social	Dufour (SF-36 social)	I=C	High
	Functioning: role limitation	Dufour (SF-36 role limitation)	I=C	High
	Disabilities of daily activities	George (ODI)	I=C	High
	Disability	Taimela (VAS-daily activities)	I=C	High
	Working ability	Taimela (Working ability)	I>C	High
	Disability	Vibe Fersum (ODI)	I>C	High
	Functional assessment	Hammond (AIMS2)	I>C	High
Levels of evidence				
Strong evidence	Consistent findings in multiple (≥3) high-quality RCTs			
Moderate evidence	Consistent findings in at least one high-quality study and at least one low-quality study, or consistent findings in multiple low-quality studies			
Inconsistent evidence	Inconsistent findings in multiple studies			
Insufficient evidence	Only 1 or 2 studies available			

Abbreviations: a-BPI-sf, Brief Pain Inventory short form, subscale impact of life; AIMS2, Arthritis Impact Measurement Scale 2; AMPS, Assessment of Motor and Process Skills; BARS-MQE, Body Awareness Rating Scale-Movement Quality and Experience; BASFI, Bath Ankylosing Spondylitis Functional Index; COOP, Darthmouth COOP Functional Health Assessment; HAQ, Health Assessment Questionnaire; HOOS-A, Hip Osteoarthritis Outcome Score, subscale Activities of daily life; HOOS-SP, Hip Osteoarthritis Outcome Score, subscale SPort and leisure; I=C, no significant differences between intervention and control group; I>C, intervention group significantly better than control group; JBLA, Joint Protection Behavior Assessment; NFAS, Norwegian Function Assessment Scale; ODI, Oswestry Disability Index; PDI, Pain Disability Index; RMDQ, Roland-Morris Disability Questionnaire; SF-36 physical, Short Form general health survey scale, subscales physical functioning; SF-36 role limitation, Short Form general health survey scale, subscale role limitations because of physical problems; SF-36 social, Short Form general health survey scale, subscale social functioning; SMT, Standardized Mensendieck Test; UCLA, University of California Los Angeles shoulder rating scale; VAS, Visual Analog Scale.

We found insufficient evidence for the effectiveness of the combined strategies on the quantity of movement behavior, as only one study with a high risk of bias was selected (table 3).

#### Activities of daily life

Fifteen studies reported on activities of daily life assessed with 23 different outcome measures (table 3). Five outcome measures were classified as of some concern, and 18 as high risk of bias. Half of these outcome measures showed significant differences in favor of the combined strategies, and the other half did not. In conclusion, inconsistent evidence was found for the effectiveness of the combined strategies on activities of daily life.

### Technique analysis

We analyzed the techniques used by the health professionals for ML, BC, and SMS based on the frameworks of Kleynen,^15^ Michie et al,^19^ and Sattoe et al^28^ (table 4). These techniques aimed to change patients’ movement behavior in daily life.

**Table 4:** Technique analysis for motor learning, behavior change, and self-management support.

	Intervention (Treatment Modalities)				Control Group
Study	Motor Learning	Behavioral Change Techniques	Self-Management Support	Exercise Therapy	
First author, Country	Sessions: number, duration Content: skills, learning strategies, type of therapist, setting Level of tailoring: Individual/Group size Underpinning model	Sessions: number, duration Content Mode of delivery Level of tailoring: Individual/Group size Underpinning model	Sessions: number, duration Content Mode of delivery Level of tailoring: Individual/Group size Underpinning model	Sessions: number, duration Content: eg, strength, flexibility, mode of delivery, type of therapist, setting Level of tailoring: Individual/Group size Underpinning model	Sessions: number, duration Content Mode of delivery Level of tailoring: Individual/Group size Underpinning model
Haugstad et al.^40^ Norway	Sessions: 10 sessions in total of 1 h over 90 d; combined ML, BCT, ET, and SMS sessions Content: Mensendieck therapy (body awareness and cognitive approach focusing on balanced posture and controlled movement, awareness of tension and relaxation, functional respiration. The Mensendieck physiotherapists teach the patient how movements are initiated mentally and transferred into body motion and how thoughts, emotions, posture, and movements are integrated and interwoven.) Level of tailoring: high. Individual therapy Underpinning model: Fitts and Posner (from cognitive awareness of movement toward gradual integration of behavioral patterns in activities of daily life.) Sensorimotor control: visual, tactile, proprioception	Content: create working alliance (therapist–patient agreement on goals of treatment, the therapist–patient agreement on interventions, the affective bond), teaching mind/body alliance, explain pain mechanisms, cognitive pain education, homework assignments, empathic listening, be own therapist, coping with challenges of daily living. Underpinning model: therapeutic working alliance		Content: exercise therapist Mensendieck performs manual release of tender and tense muscles (Sykegrep). Setting: gynecologic outpatient department of a tertiary care university hospital	Sessions: 2 Content: standard gynecologic advice at inclusion and 1 more time during the treatment period. If needed: contraceptives, analgetics, dietary or sexologic advice by gynecologist
Framework	Components of framework Kleynen: Strategy: Movement imagery (mental rehearsal) Element: Instruction, internal focus of attention	Components of framework Michie: Repetition and substitution: behavior substitution 8.2, habit formation 8.3, behavior rehearsal/practice 8.6 Goals and planning: goal setting (outcome) 1.3, goal setting (behavior) 1.1 Social support 3 Shaping knowledge: instruction on how to perform a behavior 4.1 Feedback and monitoring: self-monitoring outcome of behavior 2.4, self-monitoring behavior 2.3	Components of framework Sattoe: Mode: Individual Format: elements Educational sessions: homework assignments Motivational interviewing sessions: awareness building, problem solving, goal setting (Skills) training sessions: symptom treatment		
Asenlof et al^41^ Sweden	Sessions: 8-10 therapist-led sessions during 2-3 mo. Content: Mixed motor learning and behavioral change techniques. Principal part by patient through homework assignments between sessions. Two booster sessions after 1 and 3 mo. Session 3. Functional behavioral analysis, goal setting Session 4 and 5. Basic skill acquisition (voluntary activation of muscles, coordination of movement patterns) Session 6 and 7. Applied skill acquisition, in contrived and everyday life situations Level of tailoring: high. Individual functional behavioral analysis and therapy	Content: Session 1. Problem and goal identification (frequent and important everyday life activities) and prioritization Session 2. Self-monitoring, diary to monitor activity in everyday life Session 4-5. Cognitive skills: recognition of negative interpretation/cognition, self-efficacy reinforced by mastery experience and therapist feedback, organization of environment Session 8-12. Generalization (extend application of skills to new activities and situations, high risk situation for relapse), Maintenance and relapse prevention, Booster sessions (rehearsal, problem solving) Underpinning model: Prochaska et al^58^, Vlaeyen^59^			Sessions: 8 to 10 scheduled sessions, duration not stated Content: strengthening and muscular endurance tasks, coordination, balance, dynamic stability, flexibility and stretching, aerobic capacity exercises Components of framework (ACSM): homework, encouragement, exercise quotas, times per wk/day Mode of delivery: verbal Level of tailoring: according to patients’ physical condition. Individual therapy. Underpinning model: ACSM guidelines, “evidence-based principles.”
Framework	Components of framework Kleynen: Organization of practice environment: from simple to complex, from protective to open Organization of the task: Subdividing task followed by practicing whole task	Components of framework Michie: Goals and planning: Goal setting (behavior) 1.1 Regulation: Regulate negative emotions 11.2 Identity: self-affirmation 13.4 Feedback and monitoring: feedback on behavior 2.2, self-monitoring of behavior 2.3 Antecedents: restructuring environment 12.1/2 Repetition and substitution: behavioral rehearsal/practice 8.1, generalization of a target behavior 8.6	Components of framework Sattoe: Mode: individual Formats: elements Motivational interviewing sessions: problem solving, goal setting (Skills) training sessions: symptom treatment Educational sessions: homework assignments, daily diaries or notebooks Cognitive behavioral therapy sessions: problem solving training, homework assignments, instructions for home practice		
Dragesund et al ^42^ Norway		Sessions: 4 sessions in 4 wk of 30 min Content: The education program has 3 basic elements: Reduction of what the patients perceive as threatening inputs to the brain; Targeting the patients’ own understanding of the pain; Exposure to the threatening inputs Level of tailoring: not stated Individual/group: unclear Underpinning model: not mentioned (Moseley?)		Sessions: "once a week or every second wk for 3-6 mo" Content: Active, individualized physiotherapy according to pain problems, subsequent to Cognitive Patient Education, by physical therapist Setting: unclear Level of tailoring: "individualized" Group size: 4	Norwegian Psychomotor Therapy Sessions: "one treatment session a week or every second wk, lasting 45-60 min, for 3-6 mo" Content: readjust posture, harmonize muscle tension, harmonize breathing, harmonize movements, body awareness, provided by post graduate master level physiotherapist; setting: unclear Level of tailoring: not stated Individual Underpinning model: not stated
Framework		Components of framework Michie: Regulation: Regulate negative emotions 11.2 Shaping knowledge 4 Associations: Exposure 7.7	Components of framework Sattoe: Mode: individual Formats(elements): Motivational interviewing sessions (awareness building) (Skills) training sessions (symptom treatment) Educational sessions		Components of framework Kleynen: Element: instruction, feedback, internal focus of attention
Amris et al^44^ Denmark	Sessions: 16 two-hour sessions Content: adaptation as a mean to improve the ability to perform ADL. Adaptational strategies included changing routines, using assistive devices, modifying tasks, and physical and/or social environments. Short lectures, brainstorms, peer exchange, homework as reflective individual with coping skills, energy conservation principles. Occupational therapist in clinical unit fitted for performance of ADL (2-room flat used to observe and practice tasks in a simulated, but naturalistic home environment). Level of tailoring: high. Individual Underpinning model: not stated			Intervention and Control: prior to randomization Sessions: 5 h in 2 wk Content: The physiotherapy sessions introduced principles of graded exercise and activity (pacing principles) and included introduction to land- and water-based exercises	Intervention and Control: prior to randomization Sessions: 2 wk, scheduled 3-5 h program each day; in total 35 h. Content: standardized rehabilitation program by an interdisciplinary team consisting of a rheumatologist, a psychologist, a nurse, and occupational and physiotherapists. The occupational therapy sessions (6 h) introduced adaptational strategies for solving ADL problems. Control: Sessions: 10 two-hour educational sessions. Content: The physiotherapist supported the participants in the process of achieving the skills to identify training barriers and encouraged goal setting and strategies of pacing to increase home-based physical activity level and reduce pain exacerbations and activity intolerance,^60^^,^^61^ which is focused on increased self-selected lifestyle physical activity and restoration of physical capacity. Setting: tertiary outpatient clinic
Framework	Components of framework Kleynen: Organization of practice environment: Open/complex/limited (with environmental constraints) Organization of the task: Practicing the whole task	Components of framework Michie: Goals and planning: Goal setting (behavior) 1.1, Problem solving/coping planning 1.2, Review behavior goal(s) 1.5 Repetition and substitution: Behavior substitution 8.2, Habit formation 8.3, Behavioral rehearsal practice 8.3 Antecedents: restructuring the physical environment 12.1 Self-belief: Focus on past success 15.3 Shaping knowledge: Instruction on how to perform a behavior 4.1 Social support: social support (emotional) 3.3	Components of framework Sattoe: Mode: Individual Formats: elements Motivational interviewing sessions: awareness building, problem solving, goal setting (Skills) training sessions: symptom treatment, educational and skill training, instructions for home practice Educational sessions: homework assignments		Components of framework Michie: Repetition and substitution: Habit formation 8.3 Goals and planning: Goal setting (behavior) 1.1, Problem solving/coping planning 1.2, Review behavior goal(s) 1.5 Social support: social support (emotional) 3.3 Mode of delivery Level of tailoring: high Group: size not mentioned Underpinning model: not stated Components of framework Sattoe: Mode: Group Formats: elements Cognitive behavioral therapy sessions: goal setting, homework Skills training sessions Educational and/or support sessions: self-management plan, didactic presentations, discussions and problem solving, homework assignments, sharing experiences
Olsen et al^45^ Norway	Sessions: 12 open group sessions of 70 min. Content: Guided daily life movements: lying, sitting, standing, walking and relational movements. Movement quality practice is organized around 3 main movement elements: postural stability, free breathing, slow and mindful performance, and mental awareness of subtle movement performance. 20 min of reflective talk regarding movement experiences: insight into movement habits, increase awareness of more varied and dynamically adjustable ways of moving, and provide tools to integrate purposeful movement strategies into daily life setting. Share movement reflections, strategies to practice movements at home, and integrate them into daily life actions. Qualified BBAT primary care physiotherapist. Level of tailoring: not stated Group: 4 participants Underpinning model: "Tai chi" and Skjaerven				Sessions: 3,5h seminar Content: Dialog with patients, disease description, treatment options, optimal loading, weight regulation, physical activity/exercises. Remain physically active/self-initiated exercise, if needed physiotherapy in primary health care. Led by orthopedic surgeon and physiotherapist. Level of tailoring: unclear Group size: not stated
Framework	Components of framework Kleynen: Element: Instruction, internal focus of attention, focus on performance, explicit instructions	Components of framework Michie: Shaping knowledge Repetition and substitution: behavior substitution 8.2, behavioral rehearsal/practice 8.1, habit formation 8.3, generalization of target behavior 8.6	Components of framework Sattoe: Mode: Group Formats: elements Educational and/or support sessions: discussions and problem solving, sharing experiences, homework assignments		Components of framework Michie: Shaping knowledge Repetition and substitution: habit formation 8.3
In et al^46^ Republic of Korea	Intervention: Sessions: Two sessions of 4 h prior to core training. Content: patients were educated in detail about correct posture habits in daily life when sitting, standing, sleeping, carrying out self-hygiene (hair washing, face washing, and tooth brushing), and carrying things	Intervention: Two sessions of 4 h prior to core training. Education was conducted on (1) anatomy and kinesiology of the spine, including superficial and deep muscles of the trunk, (2) trunk muscle activity and muscle fatigue according to posture, (3) problems such as pain that can occur when the balance of trunk muscles is broken, (4) how to sit properly, and (5) a lifestyle or treatment method that can reduce pain Mode of delivery: not stated Level of tailoring: not stated Individual/Group size: not stated Underpinning model: not stated	Intervention: Two sessions of 4 h prior to core training. In addition, the MT group was educated on self-management methods (muscle relaxation using hot pack, massage ball, or foam roller) to reduce pain	Intervention: Sessions: 48 sessions core training of 40 min Content: training on pain management and lifestyle, including the supervision of the therapist for core stability exercise and exercise. Phase 1 (8 sessions): independent isometric contraction of transversus-abdominis (trA) and multifidus Phase 2 (12 sessions): cocontraction and functional tasks of the deep trunk muscles Phase 3 (12 sessions): functional task with load Phase 4 (16 sessions): functional task with an unstable surface Trunk extension exercises according to McKenzie sitting and standing Walk for 2-3 min when sitting for a long time. Level of tailoring: not stated. Individual Underpinning model: not stated	Control: Sessions: not stated Content: a 40-min core stability exercise program that included stretching and walking to be performed by patients unsupervised by physical therapist
Framework	Components of framework Kleynen: Element: Instruction: Focusing on the performance (explicit instruction), Internal focus of attention	Components of framework Michie: Shaping knowledge 4	Components of framework Sattoe: Mode: Group Formats: elements Educational and/or support sessions (didactic presentations, relaxation training)		
Soukup et al^47^ Norway	Intervention: Sessions:20 group sessions of 60 min during 13 wk Content: learning by doing daily life movements and activities, take responsibility for own health, body movements in accordance with anatomy, physiology, biomechanics (education). Body awareness during daily movement. Warmup, stretching lower limbs, ergonomic information/education, exercises trunk and pelvis, relaxation techniques. Mirrors, pictures, models. Body awareness. Application to specific, real-life situations. Working postures, lifting, carrying. Injury mechanisms education. Level of tailoring: High, subject specific advice, and sets/reps. Group of 6-10 participants Underpinning model: Mensendieck system			Variation in exercise types: isometric, dynamic, stretching, coordination, breathing, relaxation	Control: Information on Mensendieck program, but no participation in group
Framework	Components of framework Kleynen: Element: Organization of task: subdividing task and whole task Organization of practice environment: simple, protective Instruction: focusing on the performance, internal focus of attention (body awareness)	Components of framework Michie: Repetition and substitution Behavior substitution [8.2], behavior rehearsal/practice [8.1] Feedback and monitoring Feedback on behavior [2.2] Shaping knowledge 4 Instruction on how to perform a behavior [4.1]	Components of framework Sattoe: Mode: Group Formats: elements Educational and/or support sessions (didactic presentations, relaxation training), homework assignments Skills training or workshop: Practicing strategies for goal achievement Educational sessions: homework assignments		
Bendix et al^48^ Denmark	Intervention: Sessions: 1 session/d for 3 wk of 1.5 h Content: work simulation/work hardening consisted of simulated work situations, including lifting, pulling, pushing, sitting and standing workplaces, garden work, and kitchen work. Biofeedback was used in some of the sitting work situations. By occupational therapist Level of tailoring: not stated. Group of 7 participants Underpinning model: see Exercise therapy	Intervention: Sessions: 3 sessions/d for 2.5 h consisting of psychological group session, theorical class, and relaxation. Content: Behavioral approach; patients were urged to take greater responsibility for coping with pain, set realistic personal goals, change the negative sensation of pain into a more positive way of living, and give themselves credit for their achievements. It included daily group therapy and relaxation, and an average of one individual counseling session per wk. Group of 7 participants and individual		Intervention: Sessions: 3-wk, full-time, multidisciplinary program, 8 h/d; Physical therapy: 4 h/d consisting of aerobics, weight training, stretching, recreation. Follow up program of 6 h of psychological, physical, and ergonomic training, once a wk for 3 wk; Setting: Copenhagen Back Center. Level of tailoring: not stated Group of 7 participants Underpinning model: Functional restoration program (	Control: Treatment elsewhere when desired
Framework	Components of framework Kleynen: Element: Organization of practice environment: open, complex, busy, limited	Components of framework Michie: Goals and planning: goal setting 1.3/1.1, problem solving/coping planning 1.2 Self-belief: focus on past success 15.3 Regulation: regulate negative emotions 11.2	Components of framework Sattoe: Mode: Individual Formats: elements Motivational interviewing sessions: awareness building, problem solving, goal setting Skills training session(symptom treatment: relaxation Mode: Group Formats: elements Cognitive behavioral therapy sessions: goal setting, educational and skills training, fun activities, games		
Areso-Boveda et al^49^ Spain	Sessions: 6 wk, 1 session/wk, lasting 2 h and one review session (1 mo later). Exercise is part of the sessions; fifth session is just exercise. Content: Exercises were focused on body awareness and attention. Conscious breathing, proprioception, relaxation techniques and graded exposure were used. Therapist: Physiotherapist (with nurse or family doctor); Setting: Primary care: health care/physiotherapy center; Group size: 10 persons; Tailoring: at the start, not reported during intervention Underpinning model: not stated	Content: Pain neuroscience education; knowledge regarding neurophysiology of pain; such as nociception pain, proprioception, pain memory, hypervigilance, central sensitization, beliefs and memory learning. Therapist: nurse or family doctor with physiotherapist Group size: 10 persons, tailoring not reported Model: Based on PNE	Content: Neuropsychology of pain was supported with a PowerPoint presentation. A summary of the sessions and reading matter supporting what they had learned	Also exercises for mobility, coordination, strength and balance were done. Self-massage, games and music were used	Sessions: not reported Content: Usual treatment (mainly pharmacologic, tailored exercise) Individual
Framework	Components of framework Kleynen: Learning strategy: not stated Organization of environment and task: not stated. Feedback: nonverbal through proprioceptive information (tactile sensation) Instruction: focusing on the performance, internal focus of attention (conscious breathing), music	Components of framework Michie: Shaping knowledge 4 Natural consequences: information about health consequences 5.1 Repetition and substitution: graded tasks 8.7 Regulation: reduce negative emotions 11.2 Antecedents: body changes 12.6 13.2 identity: framing/reframing	Components of framework Sattoe: Mode: group Format: elements: Educational sessions: didactic presentations, written materials Training sessions: symptom treatment (relaxation techniques) Cognitive behavioral therapy sessions: educational training, written materials		
Kjeken et al^50^ Norway	Sessions: 3-wk program, number and duration not stated Content: occupational therapy intervention: could include teaching alternative working methods, use of assistive technology and discussing home and workplace accommodations (depending on the Canadian Occupational Performance Measure (COPM)) Occupational therapist; Setting: inpatient rehabilitation program; Individual program Underpinning model: not stated	Sessions: 3 wk program, number and duration not stated Content: occupational therapy intervention, could include: teaching of energy conservation and alternative working methods, discussing home and workplace accommodations; Also: information concerning fatigue management and sleep hygiene. Trying out high-quality mattresses and ergonomic pillows. Plan with patient-specific long- and short-term goals. Individualized Underpinning model: not stated		Sessions: Gym: duration: 30-45 min, frequency: 2-3 x per wk; Pool: duration: 25 min, 3-5 x per wk; Outdoors: duration: 45-60 min, 3 x per wk Content: muscle strength, stability, mobility, cardio-respiratory fitness (in gym, pool and outdoor physical activities). Therapist: physiotherapist Tailoring: doses, intensity and frequency was individually adopted, physiotherapist designed a weekly exercise program	Sessions: not stated Content: treatment as usual (which could include: consultations with a rheumatologist of physician, community-based physiotherapy and/or self-management in terms of physical activity and exercises). Individual
Framework	Components of framework Kleynen: Learning strategy, organization of environment and task, feedback: not stated Instruction: focusing on the performance	Components of framework Michie: Goals and planning: goal setting (outcome) 1.3 Shaping knowledge: instruction on how to perform a behavior 4.1 Antecedents: restructuring physical environment 12.1, adding objects to the environment 12.2	Components of framework Sattoe: Mode: individual Format: elements: Educational session: discussion and problem solving Motivational interviewing sessions: goal setting Training sessions: symptom treatment Cognitive behavioral therapy sessions: educational and skills training		
Monticone et al^51^ Italy	Session: one 60-min session per wk, during 20 wk Content: Self-correction scoliosis progressively performed during task-oriented exercises (eg, sit-to-stand, ascending/descending stairs) aimed at improving postural, proprioception, and neuromotor control of the spine and limbs. Additional task-oriented exercises (eg, turning, standing on unstable surfaces, and walking while changing speed and direction). Patients were helped to increase their level of activity by graded exposure to exercise and common activities of daily life. Patients were asked to continue exercises at home. Therapist: Physiotherapist (under supervision of physiatrist). Setting: not stated. Individual/group: not reported; Underpinning model: not reported	Sessions: twice a month for a 60-min, 20 wk. Content: cognitive behavioral strategies: patients were educated to view scoliosis as something that can be self-managed; Patients were helped to increase their level of activity by graded exposure to exercises and to common activities of daily life (main situations avoided were identified). And by communication aimed at sharing the goals to be reached. Spouses and significant others were asked to support patients’ compliance during the study and to inform of any difficulty if encountered. Therapist: psychologist Individual/group: not reported; Underpinning model: not reported		Content: Active self-correction scoliosis, exercises for strengthening spinal deep muscles while maintaining self-correction, segmentary stretching involving limbs and back muscles	Session: one 60-min session per wk, during 20 wk. Content: General physiotherapy: exercises spinal mobilization, passive mobilization, muscle segmentary stretching of upper/lower limb and back muscles, strengthening of abdominal and back muscles, and postural control spine and pelvis. therapist: physiotherapist Individual Underpinning model: not stated
Framework	Components of framework Kleynen: Learning strategies not stated. Organization of task: from simple to complex. Organization of environment: not stated. Feedback: not stated Instructions: focusing on the performance	Components of framework Michie: Goals and planning: goal setting (outcome) 1.3 Social support: 3 Shaping knowledge: instructions on how to perform a behavior 4.1 Repetition and substitution: graded tasks 8.7 Antecedents: body change 12.6 Identity: framing/reframing 13.2	Components of framework Sattoe: Formats: elements Educational session Motivational interviewing sessions: goal setting Training sessions: symptoms treatment Cognitive behavioral therapy: educational		
Dufour et al^52^ Denmark	Sessions: 12 wk; biweekly motor learning and behavior change techniques (in total 10 h) Content: Postural techniques provided by physiotherapist. Back care and lifting techniques by an occupational therapist Setting: not stated. In groups of 6 patients Underpinning model: Based on Bendix et al.^48^	Sessions: 12 wk; biweekly motor learning and behavior change techniques (in total 10 h) Content: Lessons of anatomy and pain management. Provided by physiotherapist. In groups of 6 patients		Sessions: 12 wk, 3 times a wk a 2 h session (in total 75 h). Content: aerobic training and training to strengthen the muscles in the back, gluteus region, and abdominal wall. In addition: play ball games, training in hot water, ball stick training. Therapist: physiotherapist, Setting: not stated. During the second and third part the sessions were performed at home or in a fitness center	Session: 1 h, twice a wk, 12 wk (in total 22 h). Content: muscle training exercises in the back and gluteus region: body and leg lifting in prone position, supplemented with exercises aimed at dynamic contraction of painful muscles. Trained therapist Individual program Underpinning model: Oefeldt
Framework	Components of framework Kleynen: Learning strategy not stated. Organization of environment: from protected to open (exercise was gradually moved to home or in fitness center) Organization of task, feedback and instruction: not stated	Components of framework Michie: Shaping knowledge 4 Antecedents: body changes 12.6	Components of framework Sattoe: Mode: Group Formats: elements: Educational session Training sessions: symptoms treatment Cognitive behavioral therapy: educational and skill training		
George et al^53^ United States of America (Florida)	Sessions: Daily average of 3 h exercise and activities, 3-5 wk Content: activities that mimicked job duties in material handling (including lifting, carrying, and fine motor skills) and activities of daily living tasks (including cleaning and cooking). Principles of graded exposure were used (also positive reinforcement). Therapist: Physical therapist, setting: inpatient rehabilitation Individual and group: group exercise sessions, individual-tailored Underpinning model: Fear-avoidance model of musculoskeletal pain	Sessions: daily 0,5-1 h relaxation, 0,5-2 h education Content: Education and treatment in pain-coping strategies, relaxation training, pain-related fear and catastrophizing, dysfunctional pain response patterns, anger management, Cognitive and behavior strategies to reduce emotional distress. Use of positive reinforcement. Encouraging limited use of opiate medication. Individual and group: group sessions are individual-tailored		Content: Flexibility training lower extremity and spine, stabilization training lumbar musculature, strength training extremities, cardiovascular training. Principles of graded exposure were used to dose exercise and physical activity	The same program as intervention. The principles of graded exercise were used to dose exercise and physical activity
Framework	Components of framework Kleynen: Learning strategy not stated. Organization of task: from simple to complex; organization of environment: not stated Feedback and instructions: not stated.	Components of framework Michie: Goals and planning: problem solving 1.2 Shaping knowledge: instruction on how to perform a behavior 4.1, re-attribution 4.3 Natural consequences: information about health consequences 5.1, information about emotional consequences 5.6 Repetition and substitution: graded task 8.7 Reward and threat Regulation: reduce negative emotions 11.2 Antecedents: body changes 12.6	Framework Sattoe Mode: individual/group: Format/elements: Educational sessions; Motivational interviewing sessions: awareness building, problem solving; Training sessions: symptom treatment (relaxation); Cognitive behavioral therapy sessions: educational and skill training.		
Taimela et al^54^ Finland	Sessions: 12 wk, 2 sessions/wk, ∼45 min for total treatment (exercise, motor learning, and behavior change) Content: Proprioceptive exercises and relaxation; Proprioceptive training with specific devices for fixation of the trunk, hip, knees, and feet. Therapist: physical therapists; Setting: not reported Individual of group: not reported, level of tailoring: not reported	Session: During treatment sessions were discussions. Duration not stated. Content: behavioral and cognitive support concerning the benign nature and good prognosis of nonspecific cervical pain. And Behavior support to reduce fear of pain. A lecture about neck pain and its consequences. A record was kept of the progression of loading and the course of pain during the treatment.		Content: Cervicothoracic stabilization training, relaxation training, eye fixation exercises, training postural control. Written information about home exercises.	Control1 Sessions: 2 training sessions in smaller groups and one lecture. Content: a lecture about neck pain and its consequences, practical training for their home exercises, written information about neck exercises and diary for maintaining a progress. Control2 Sessions: one lecture Content: Lecture about neck pain and its consequences. And written information about neck exercises to be applied at home and at the workplace.
Framework	Components of framework Kleynen: Learning strategy: not reported Organization environment and task: not reported Feedback: nonverbal through proprioceptive information. Instruction: not reported	Components of framework Michie: Shaping knowledge 4 Natural consequences 5 Regulation: reduce negative emotions 11.2 Antecedents: body changes 12.6	Components of framework Sattoe: Mode: individual/group: not stated. Formats: elements: Educational sessions: notebook Skill training sessions: symptom treatment (like relaxation techniques) Cognitive behavioral therapy sessions: educational and skills training (didactic presentation, discussions)		
Vibe Fersum et al,^55^ Vibe Fersum et al^56^ (follow up) Norway	Sessions: Content: Aim was enhanced body awareness and provide patients with alternative strategies to normalize their postural and movement behaviors. Activities of daily living were rehearsed for confident and mindful normalizing their movement behaviors for performing activities in daily living. Graded exposure to previously pain provocative tasks, in a relaxed and controlled manner; with feedback (visual with the use of mirrors, mental imagery, and awareness of body responses such as breath holding, muscle guarding, and changes in respiration rate). Augmented by dynamic practical demonstration of the postures or movement by the therapist, the use of mirrors to enhance body schema awareness, written instruction, and stick body diagrams.	Session: Content: Vicious cycle of pain was outlined in a diagram (based on findings examination and the Orebro MPQ). Explanation vicious cycle: negative beliefs pain, fear of movement, increased focus, low mood and poor pacing reinforced avoidance and protective behaviors. Muscle guarding, altered movement patterns and body postures fed a vicious cycle of pain sensitization and disability. Openly discussion about breaking the cycle and set their goals and planning for management. Individual, tailored		Content: Aim to restore normal functional movement capacity. Simple nonthreatening exercises were gradually progressed toward higher load and more complex functional exercises. A conditioning program for strength and endurance (when required for functional goals) Patients were asked to do to carry out some form of physical exercise (such as walking or biking) 3-5 times a wk.	Sessions: Generally 30 min a session (1 h for the initial consultation). Content: Manual therapy. In addition, most patients (82.5%) were given exercises or a home exercise program: general exercise or motor control exercise.
Framework	Components of framework Kleynen: Framework Kleynen: Learning strategy: observational learning, movement imagery Organization of the task: from simple to complex. Organization of the practice environment: not stated Feedback: verbal, nonverbal through mirror and proprioceptive information. Instruction: focusing on the performance, internal focus of attention.	Components of framework Michie: Goals and planning: problem solving 1.2, goal setting 1.3, action planning 1.4 Shaping knowledge: instruction on how to perform a behavior 4.1 Natural consequences: information about health consequences 5.1, information about emotional consequences 5.6 Comparison of behavior: demonstration of the behavior 6.1 Repetition and substitution: graded task 8.7 Regulation: reduce negative emotions 11.2 antecedents: body changes 12.6	Components of framework Sattoe: Mode: individual Formats: elements: Motivational interviewing sessions: awareness building, problem solving, goal setting (skill) training sessions: symptom treatment cognitive behavioral therapy sessions: educational and skills training		
Hammond and Freeman^57^ United Kingdom	Sessions: two-thirds of the program was spent on practicing hand-joint protection methods. Content: Patients were shown a range of options for task performance. They could select which methods worked best for them. Practice started with blocked repetition of single actions and progressed to sequences of activities. Verbal, visual, and kinesthetic instruction was given, supported by extrinsic and intrinsic feedback on performance. Mental rehearsal of actions was also included. Subjects practiced in pairs or threes. Underpinning model: not reported	Sessions: combination of all strategies: 4 sessions of 2 h, total: 8 h. Content: educational, behavioral and self-efficacy enhancing strategies to increase adherence joint protection. Information was provided on the disease process, outcome of RA and drug therapy. Contracting and goal setting were used to promote joint protection at home. Subjects were asked to self-monitor hand use patterns and pain / discomfort levels during daily activities. Participants were encouraged to write goals in their workbooks. Feedback was given on the process and problems every beginning of the sessions. Individuals’ practical problems were discussed and group members used problem solving methods to generate solutions. Type of therapist for all strategies: experienced rheumatology occupational therapist Setting: outpatient at rheumatology department of hospital Group between 4-8, with partners included. Level of tailoring: also focus on individual. Underpinning model: based on the health belief model and theories of social learning and self-management	Participants were provided with an information pack and workbook detailing the principles of joint protection, with photographs of a range of joint protection methods		Sessions: 4 sessions of 2 h, total: 8 h. Content: Information about RA, treatment and controlling pain. Demonstration and practice of exercise and relaxation. Also time was spent on joint protection: information of principles, demonstration some methods, discussion about problem activities and solutions, demonstration and practice protection methods applied to a daily activity. Setting: outpatient at rheumatology department of hospital. Therapists: nursing, medical, occupational therapy, physiotherapy staff Group between 6-12, with partners included. Level of tailoring: not reported. Underpinning model: joint protection part is typical program provided in the UK
Framework	Components of framework Kleynen: Learning strategy: observational learning, movement imagery Organization of the task: subdividing the task, blocked repetition. Organization of the practice environment: department Feedback: verbal on performance Instruction: verbal and visual	Components of framework Michie: Goals and planning: goal setting 1.1, problem solving 1.2, goal setting 1.3, behavioral contract 1.8 Feedback and monitoring: feedback on the behavior 2.2, self-monitoring 2.3 Social support: social support 3.1 Shaping knowledge: instruction on how to perform the behavior 4.1 Comparison of behavior: demonstration of the behavior 6.1 Repetition and substitution: behavioral practice/rehearsal 8.1, generalization of a target behavior 8.6	Components of framework Sattoe: Mode: group Educational and support sessions: didactic presentations, workbook, leaflets Cognitive behavioral therapy sessions: goal setting, problem solving, discussion Skills training: protection methods		

### Motor learning

#### Sessions

Patients explicitly worked toward different performance strategies for daily activities in all but 2 studies.^42^^,^^54^ This concerned the following activities of daily life: grooming, cooking, cleaning, sitting, standing, transfers, gait, stairs negotiating, lifting, and fine motor skills. Most studies focused on the home environment, but 3 worked toward the working context of the patient.^48^, ^50^, ^53^ The learning process was guided by occupational therapists,^43^, ^48^, ^50^, ^52^, ^57^ specialized exercise therapists in Basic Body Awareness^45^ or Mensendieck,^40^^,^^47^ or physical therapists. The number of therapy sessions varied from 4^42^ to 20,^47^^,^^51^ taking 45 minutes^54^ ≤4 hours,^46^ over periods ranging from 3^48^ to 20 weeks.^51^

#### ML techniques

The technique of learning was rarely stated in the introduction or method sections of the selected studies. A more explicit form of ML was dominant in the interventions, in which patients were consciously aware of the learning process. For example, Hammond and Freeman^57^ reported the use of observational learning and movement imagery. In 3 studies, the chosen learning technique could be deduced. Vibe Fersum et al.^56^ and Soukup et al.^47^ used observational learning and movement imagery. Haugstad et al.^40^ applied movement imagery as well. For instruction, internal focus of attention was used most,^40^, ^45^, ^46^, ^47^, ^49^^,^^54^^,^^56^ sometimes accompanied by simultaneous focus on performance.^45^^,^^46^^,^^47^, ^57^ Also, explicit instructions were incorporated.^45^^,^^46^, ^57^ One study used music.^49^ Feedback was largely not reported. Vibe Fersum et al.^56^ and Soukup et al.^47^ described nonverbal feedback by using mirrors. Further, Hammond and Freeman^57^ supported the learning process by extrinsic and intrinsic feedback. Organization was reported more explicitly than instruction and feedback. Often patients practiced parts of their daily activities in a safe environment accompanied by the therapist. The organization of this approach progressed from simple to complex and from protective to open,^41^, ^43^, ^47^, ^48^, ^49^, ^51^, ^52^, ^53^, ^56^, ^57^ preparing patients for autonomous performance within their home or work environment. The activities themselves were sometimes subdivided and then practiced as a whole^41^, ^47^, ^51^ or only practiced as a whole.^43^

### Behavior change

#### Sessions

BC and SMS were mostly integrated within one session,^40^, ^44^, ^45^, ^47^, ^49^, ^50^, ^52^, ^54^, ^57^ while others organized separate sessions.^42^, ^46^, ^48^, ^51^, ^53^, ^56^ Patients were guided by a psychologist,^51^ nurse or family doctor,^49^ occupational therapists,^43^, ^48^, ^50^, ^57^ physical therapist,^52^, ^53^, ^54^, ^56^ specialized exercise therapists in Basic Body Awareness,^45^ or Mensendieck.^40^^,^^47^ The number of therapy sessions varied from 2^45^ to 24,^54^ taking 30-45 minutes^56^ or ≤4 hours,^46^ over periods ranging from 3^50^ to 20 weeks.^51^

During the sessions, therapists educated patients on the mechanism of chronic pain.^40^, ^42^, ^46^, ^49^, ^50^, ^51^, ^52^, ^53^, ^54^, ^56^ Some studies also taught cognitive and behavioral skills to address negative beliefs, emotions, and pain behavior.^40^, ^41^, ^42^, ^44^, ^48^, ^51^, ^52^, ^53^, ^57^ Furthermore, some studies explicitly paid attention to the adaptations of the social and physical environment.^41^, ^43^, ^50^, ^57^ Most studies used goal formulation and planning.^41^, ^43^, ^48^, ^50^, ^51^, ^53^, ^56^, ^57^ Self-efficacy was explicitly addressed to gain confidence to change.^40^, ^41^, ^48^, ^53^, ^56^, ^57^

#### SMS sessions

Six studies^40^, ^41^, ^44^^,^^42^^,^^50^^,^^56^ used an individual approach, while 6 others used group sessions.^45^, ^46^, ^47^, ^49^, ^52^, ^57^ Two studies used a combination.^48^^,^^53^ Monticone et al^51^ and Taimela et al^54^ did not clearly describe the approach.

#### BC techniques

The specific technique “Instruction on how to perform the behavior” and the category Shaping knowledge were most common in our selected studies.^40^, ^42^, ^43^, ^45^, ^46^, ^47^, ^49^, ^50^, ^51^, ^52^, ^53^, ^54^, ^56^, ^57^ Other often used specific BCTs were goal setting and problem solving.^40^, ^41^, ^43^, ^48^, ^50^, ^51^, ^53^, ^56^, ^57^ The interventions often consisted of the category repetition and substitution.^40^, ^41^, ^43^, ^45^, ^47^, ^49^, ^51^, ^53^, ^56^, ^57^ The specific BCT body changes were also widely used,^49^, ^51^, ^52^, ^53^, ^54^, ^55^ as was reducing negative emotions.^41^, ^42^, ^48^, ^49^, ^53^, ^54^, ^56^ A smaller part of the selected studies^40^, ^41^, ^47^, ^57^ used the specific BCT feedback on behavior and the self-monitoring of behavior. In some interventions, the category social support^40^, ^43^, ^51^, ^57^ and the specific BCT “Information about health and emotional consequences,”^49^, ^53^, ^54^, ^56^ demonstration of the behavior,^56^, ^57^ and habit formation^40^, ^43^^,^^50^ were applied. Also, the specific BCTs “Restructuring the physical and the social environment,”^41^, ^43^, ^50^ framing/reframing,^49^, ^51^ and focus on past success^43^, ^48^ were sometimes used. For detailed description, see table 4.

#### SMS techniques

Almost all studies^40^, ^41^, ^42^, ^43^, ^45^, ^46^, ^47^, ^49^, ^50^, ^51^, ^52^, ^53^, ^54^, ^57^ used educational sessions. These could include discussions and problem solving, sharing experiences, homework assignments, didactic presentations, written materials, daily diaries, and notebooks. Training sessions were also commonly used^40^, ^41^, ^42^, ^43^, ^48^, ^49^, ^50^, ^51^, ^53^, ^56^, ^57^and contained symptom treatment, educational and skill training, and instructions for home practice. Another widely used technique was motivational interviewing.^40^, ^41^, ^42^, ^43^, ^45^, ^46^, ^48^, ^50^, ^51^, ^53^, ^56^ Finally, cognitive behavioral therapy sessions were implemented,^40^, ^41^, ^42^, ^43^, ^45^, ^46^, ^49^, ^50^, ^51^, ^53^, ^57^ such as educational and skills training.

#### Exercise therapy

In most studies, patients performed exercises addressing the prerequisites of healthy movement behavior. According to individually tailored training parameters, they exercised to increase motor fitness. Exercises aimed to increase muscular strength,^48^, ^49^, ^50^, ^52^, ^53^, ^56^ increase flexibility,^47^, ^48^, ^49^, ^53^ reduce muscle tension,^40^, ^47^, ^49^, ^54^ stabilize the spine,^46^, ^51^, ^53^, ^54^ increase aerobic or cardiac endurance,^43^, ^48^, ^49^, ^50^, ^52^, ^53^, ^56^ or increase balance.^49^ In rehabilitation centers, this was often trained in groups ≤7 patients and also in the form of land or water games.^48^

#### Control group

In most studies, the control group received potentially effective treatment, but this treatment did not combine ML with BC. In George et al’s study,^53^ however, the control group received the same program as the intervention group, but with a graded activity approach. In 3 studies,^41^, ^51^, ^52^ the control intervention consisted of weekly sessions of exercise therapy to improve physical functioning. In the study by Amris et al,^43^ patients received multiple education and exercise sessions to improve physical activity and capacity. The control group of Vibe Fersum et al^56^ had several sessions of manual therapy combined with general and motor control exercises. Two studies^50^^,^^49^ used usual treatment as control. The control group of Hammond and Freeman^57^ received standard arthritis education: information, exercises, and practice of joint protection. Also, studies with a less intensive control intervention were selected. Controls attended a seminar focused on physical activity,^45^ received unsupervised exercises,^46^ or received information on a Mensendieck treatment,^47^ standard gynecologic advice,^40^ or only treatment when desired.^48^ One control intervention consisted of a lecture and 2 training sessions for exercises, whereas patients in the other control group attended a seminar with written information on exercises.^54^

## Discussion

This review investigated the effectiveness of combined ML and BC strategies to improve the movement behavior of patients with CMP in their natural environment. The second aim was to provide an in-depth analysis of the techniques applied within these strategies.

### Evidence synthesis

The best evidence synthesis revealed moderate evidence in favor of the combined strategies over the control groups on the quality of movement behavior, whereas evidence on the quantity of movement behavior was insufficient. The effectiveness of the interventions on activities of daily life showed inconsistent evidence. This inconsistent evidence suggests these strategies may not uniformly enhance functional outcomes, highlighting the need for further research. These results should be interpreted with caution because of the limited number of studies, heterogeneity in control groups, diagnosis, and session doses. Moreover, small sample sizes and the lack of defined criteria for duration, frequency, sessions, or techniques may have influenced our findings.

Patients with CMP often show reduced quality and quantity of movement behavior,^11^, ^12^, ^13^, ^14^ making improvement of movement behavior a primary treatment goal in clinical practice. Measuring movement behavior is therefore essential. However, there are few appropriate tools available for this purpose.^62^ Consequently, only a few studies used movement behavior as an outcome measure. This may have affected the robustness of our findings and limited the ability to fully assess the interventions’ impact on this outcome measure. Future studies should prioritize movement behavior as a primary outcome to provide robust evidence on intervention effectiveness.

Despite the limited number of studies reporting on movement behavior, the evidence for the quality of movement behavior was of moderate strength. This is both an interesting and promising result, suggesting that patients with CMP can improve their everyday movement behavior, such as maintaining posture or enhancing symmetry during daily activities. These meaningful changes may lead to reduced difficulties in daily activities and improved overall daily functioning.

Notably, we identified more studies assessing the quality of movement behavior rather than quantity, despite the latter being widely reported in the literature, particularly in studies using wearables.^63^ This may be attributed to our stringent combination of search criteria, which required both ML and BC strategies. Given that ML strategies inherently focus on improving movement quality, research addressing the effect of ML strategies on the quantity of movement behavior appears to be less common. This may have influenced our findings. Future research could explore the effect of exercise therapy on the quantity of movement behavior in patients with CMP in greater depth. Adjusting our search strategy by omitting ML terms could facilitate such exploration.

### Technique analysis

The analysis revealed variation in the delivery, setting, dosage, and techniques for ML and BC. These techniques, particularly related to ML, were often described insufficiently. Therefore, it remains unclear which strategy components or combinations of ML and BC techniques are most effective. Future studies should provide more detailed information, helping to identify factors contributing to the effectiveness of the combined strategies.

The ML techniques in this review predominantly align with an explicit learning approach, in which patients are consciously aware of the learning process.^16^ Examples include observational learning, movement imagery, instruction with an internal focus of attention, and an emphasis on performance. As both explicit and implicit approaches are essential for the acquisition of motor skills,^15^^,^^16^ it could be beneficial for patients with CMP to also learn implicitly to better incorporate new movement behavior in their daily life.

Although we analyzed all techniques used in the interventions, a small subset of available BCTs and SMS techniques were applied. We identified only one-third of the techniques described by Michie et al.^19^ Most often reported were shaping knowledge and “instruction on how to perform,” goal setting, problem solving, behavioral practice, behavioral substitution, generalization, graded tasks, body changes, and “reducing negative emotions.” This finding is consistent with a previous scoping review^60^ and systematic reviews.^26^^,^^64^^,^^65^ However, at the level of single studies, an even smaller subset of BCTs and SMS was applied. The interventions used between 1 and 11 BCTs.

Although the combined strategies were oriented toward alternatives to patients’ movement behavior in their daily life, it remains unclear whether these interventions were sufficiently context-oriented to facilitate the application of new behavior to the patients’ own daily lives. It is suggested that practicing new movement behavior in a simulated home or work environment may be helpful for this purpose.^43^ Another approach is to learn adaptations for the patient’s own physical and social environment. 41, 43, 50, 57 Practicing within the actual context of the patient appears to be the most effective method to reduce transfer problems. However, this was not reported in any of the studies included.

### Study limitations

To the best of our knowledge, this is the first study to provide an in-depth analysis of the techniques for ML and BC used by health professionals involved in movement care of patients with CMP. We used 3 different frameworks for this analysis, enabling a detailed description of the techniques (table 4). Furthermore, the blocks of these frameworks resulted in a comprehensive set of search terms (supplemental appendices S1-S3) for the intervention strategies. Thus, a second key strength of our systematic review lies in the comprehensive and inclusive search strategy. This is supported by the large number of articles retrieved in the search (48,820). Additionally, we used a powerful AI-assisted screening tool with broad stopping criteria to ensure thoroughness. The tool was trained using prior knowledge to support comprehensive screening.^33^ By providing prior knowledge covering a wide range of diagnoses, intervention types, and outcome measures, we avoided analyzing only a subset of the dataset (trap formation^66^) while still meeting our inclusion criteria. In addition, we trained the algorithm with another 10 diverse studies that did not fully meet the criteria. Before using the screening tool, 3 calibration rounds were conducted to ensure consistent interpretation of the inclusion criteria. Future reviews could use alternative sets of studies as prior knowledge to assess the robustness of the results*.* Despite this extensive search, we may have missed a limited number of papers because of not searching in registries for ongoing studies.^67^ In addition, because of our search was conducted by the end of January 2023, we may have missed relevant, recent studies.

Because of the limited number of included studies and the substantial heterogeneity in diagnoses, control groups, as well as the dosage and content of strategies, we refrained from a meta-analysis and performed an evidence synthesis instead. As a result, the conclusions should be interpreted cautiously. Moreover, many studies were rated as having a high risk of bias because of the limited information provided in the papers. Furthermore, a lack of blinding of caregivers and patients, although often unfeasible in studies involving ML and BC, may have played a role. However, post hoc analysis without these blinding aspects did not show a lower risk of bias.

Although we made efforts to identify the specific ML techniques and BCTs, some studies provided insufficient detail on the interventions. This made it difficult to determine exactly which specific techniques were applied, and other reviewers might have classified certain techniques differently. Additionally, the researchers were not formally trained in the ML framework of Kleynen^15^ or the BCT taxonomy of Michie et al.^19^ However, they are deeply familiar with ML techniques and BCTs through their teaching at their institutions, education, and clinical practice in specialized exercise therapy.

### Implications for research and practice

The first recommendation is that frameworks can be used for comprehensive planning, implementing, and documenting intervention strategies. In our review, we used the frameworks of Kleynen,^15^ Michie et al,^19^ and Sattoe et al.^28^ These frameworks offer the opportunity to describe the applied strategies of the interventions in detail, improving reproducibility.

Second, there is a need for measuring movement behavior both qualitatively and quantitatively. For future trials, we recommend applying these outcome measures to provide more clinically relevant information about the improvement of patients’ movement behavior in their own context.^10^ We recommend the same for health professionals to assess both quality and quantity of movement within the patients’ context.

Last, we recommend that health professionals develop a large skill set of diverse techniques for ML and BC to change the movement behavior of their patients with CMP. Training is essential, as Hutting et al^68^ found that professionals expressed a need for knowledge and tools to support patients in their learning process. Additionally, we suggest that professionals should consciously choose a wide variety of techniques fitting the characteristics of the patients, the task to be learned, the learning circumstances, and the patient’s learning process^15^. Also, evidence suggests that interventions employing a greater number of BCTs (>8 techniques) result in a higher effect.^26^^,^^64^

## Conclusions

We found moderate evidence in favor of combined ML and BC strategies over the control groups on the quality of movement behavior, but insufficient evidence for the impact on movement quantity. For activities of daily life, the effectiveness of the interventions showed inconsistent results.

The interventions used a limited set of ML and BC techniques. Most used ML techniques were observational learning, movement imagery, instruction with an internal focus of attention, and an emphasis on performance. Frequently applied BCTs were shaping knowledge and “instruction on how to perform,” goal setting, problem solving, behavioral practice, behavioral substitution, generalization, graded tasks, body changes, and “reducing negative emotions.” We recommend that future trials and health professionals use movement behavior as an outcome measure and to apply a more diverse set of ML and BC techniques during interventions.

## Suppliers

a. EndNote, version 20; Clarivate. b. ASReview, Utrecht University. c. Risk of bias tool; Cochrane.

## Disclosure

M.T., B.V., and M.W. report serving as members of the scientific council of the Dutch Society for Exercise Therapists (VvO). The other authors have nothing to disclose.

## Appendix Supplementary materials

Image, application 1

Image, application 2

Image, application 3

## Footnotes

Supplementary material associated with this article can be found, in the online version, at doi:10.1016/j.arrct.2026.100618.
