# Supplementary material for: Evaluating the Effectiveness and Technique Composition of Combined Motor Learning and Behavior Change Strategies for Movement Behavior in Chronic Musculoskeletal Pain: A Systematic Review
Source: Arch Rehabil Res Clin Transl. 2026 Mar 24;8(3):100618. doi: 10.1016/j.arrct.2026.100618 (PMC13591100; doi:10.1016/j.arrct.2026.100618)
Supplement: Supplementary file 1 [file mmc1.docx]

Supplement 1. Search in Pubmed

P: Patiënt

(("Chronic Pain"[Mesh] OR ((chronic[tiab] OR persist*[tiab] OR recurren*[tiab] OR "long-standing"[tiab] OR ongoing[tiab] OR "ongoing"[tiab] OR continu*[tiab] OR prolong*[tiab]) AND (Pain*[tiab] OR Ache*[tiab] OR complain*[tiab]))) OR ("Musculoskeletal Diseases"[Mesh:NoExp] OR msd[tiab] OR "Musculoskeletal Pain"[Mesh] OR ((musculoskeletal[tiab] OR muskuloskeletal[tiab] OR "musculo-skeletal"[tiab] OR "muskulo-skeletal"[tiab] OR muscle*) AND (Disease*[tiab] OR Pain*[tiab] OR ache*[tiab] OR condition*[tiab] OR Disorder*[tiab] OR problem* OR soreness*[tiab] OR tenderness*[tiab]) ((chronic AND (Disease* OR condition* OR Disorder* OR problem* OR soreness* OR tenderness*)):ti,ab,kw) OR "Arthralgia"[Mesh] OR arthralgia[tiab] OR myalgia[tiab] OR "Back Pain"[Mesh:NoExp] OR "backpain*"[tiab] OR "backache*"[tiab] OR "Low Back Pain"[Mesh] OR lumbago*[tiab] OR LBP[tiab] OR "Sciatica"[Mesh] OR Sciatica[tiab] OR "Neck Pain"[Mesh] OR "neckpain*"[tiab] OR "neckache*"[tiab]) OR cervicalgia*[tiab] OR cervicodynia*[tiab] OR "Whiplash Injuries"[Mesh] OR whiplash*[tiab] OR "acceleration cranial trauma*"[tiab] OR "deceleration cranial trauma"[tiab] OR ADT[tiab] OR "Shoulder Pain"[Mesh] OR "Shoulder Impingement Syndrome"[Mesh] OR "shoulder impingement*"[tiab] OR "rotator cuff impingement*"[tiab] OR "coracohumeral impingement*"[tiab] OR "coraco-humeral impingement*"[tiab] OR "coracoid impingement*"[tiab] OR "subacromial impingement*"[tiab] OR "sub-acromial impingement*"[tiab] OR "outlet impingement*"[tiab] OR "internal impingement*"[tiab] OR "glenoid impingement*"[tiab] OR "Bursitis"[Mesh] OR bursitis[tiab] OR bursitides[tiab] OR "frozen shoulder*"[tiab] OR capsulitis[tiab] OR capsulitides[tiab] OR "shoulderpain*"[tiab] OR "shoulderache*"[tiab] OR "Elbow Tendinopathy"[Mesh] OR "elbow tendinopath*"[tiab] OR epicondylitis[tiab] OR epicondylitides[tiab] OR "tennis elbow*"[tiab] OR "golfers elbow*"[tiab] OR "golfer's elbow*"[tiab] OR "golf elbow*"[tiab] OR "Nerve Compression Syndromes"[Mesh] OR "nerve compression syndrome*"[tiab] OR "entrapment neuropath*"[tiab] OR ((plexus[tiab] OR nerve[tiab]) AND (impingement*[tiab] OR entrapment*[tiab])) OR "Carpal Tunnel Syndrome"[Mesh] OR "carpal tunnel"[tiab] OR "cubital tunnel"[tiab] OR "Ulnar Nerve Compression Syndromes"[Mesh] OR "ulnar nerve compression*"[tiab] OR "ulnar nerve external compression*"[tiab] OR "ulnar nerve entrapment*"[tiab] OR "Radial Neuropathy"[Mesh] OR "radial neuropath*"[tiab] OR "supinator syndrome*"[tiab] OR "radial nerve disease*"[tiab] OR "radial mononeuropath*"[tiab] OR "wrist-drop"[tiab] OR "radial nerve entrapment*"[tiab] OR "radial tunnel syndrome*"[tiab] OR "Thoracic Outlet Syndrome"[Mesh] OR (("thoracic outlet"[tiab] OR "thoracic aperture"[tiab] OR "thorax outlet"[tiab] OR "thorax aperture"[tiab] OR Costoclavicular[tiab] OR "scalenus anticus"[tiab] OR "cervical rib"[tiab] OR Naffziger[tiab] OR Naffzigers[tiab] OR Naffziger's[tiab]) AND syndrome*[tiab]) OR tos[tiab] OR "complaints arm neck shoulder*"[tiab] OR cans[tiab] OR "Cauda Equina Syndrome"[Mesh] OR "cauda equina syndrome"[tiab] OR "Piriformis Muscle Syndrome"[Mesh] OR "piriformis muscle syndrome*"[tiab] OR "piriformis syndrome*"[tiab] OR "Tarsal Tunnel Syndrome"[Mesh] OR "tarsal tunnel syndrome*"[tiab] OR "Intervertebral Disc Displacement"[Mesh] OR ((displacement*[tiab] OR protru*[tiab] OR hernia*[tiab] OR slipped[tiab] OR prolapse*[tiab]) AND (disk*[tiab] OR disc*[tiab])) OR "Spinal Stenosis"[Mesh] OR "spinal stenosis"[tiab] OR "spinal stenoses"[tiab] OR "vertebral canal stenos*"[tiab] OR "Fatigue Syndrome, Chronic"[Mesh] OR "chronic fatigue"[tiab] OR "Fibromyalgia"[Mesh] OR fibromyalgi*[tiab] OR "muscular rheumatism"[tiab] OR fibrositis[tiab] OR fibrositides[tiab] OR "myofascial pain"[tiab] OR "Osteoporosis"[Mesh] OR osteoporosis[tiab] OR osteoporoses[tiab] OR "osteo-porosis"[tiab] OR "osteo-poroses"[tiab] OR "bone loss*"[tiab] OR "Spondylarthritis"[Mesh] OR spondylarthritis[tiab] OR spondyloarthritis[tiab] OR spondylarthritides[tiab] OR spondyloarthritides[tiab] OR spondyloarthropath*[tiab] OR "spondylo-arthropath*"[tiab] OR spondylarthropath*[tiab] OR "**s**pondyl-arthropath*"[tiab] OR "Spondylitis, Ankylosing"[Mesh] OR "ankylosing spondylitis"[tiab] OR "Bechterew*"[tiab] OR "spondylarthritis ankylopoietica*"[tiab] OR "spondyloarthritis ankylopoietica*"[tiab] OR "ankylosing spondylarthritides*"[tiab] OR "ankylosing spondyloarthritis*"[tiab] OR "ankylosing spondyloarthritides*"[tiab] OR "spondylitis ankylopoietica*"[tiab] OR "Osteoarthritis"[Mesh] OR "osteoarthritis"[tiab] OR "osteoarthritides"[tiab] OR "osteoarthrosis"[tiab] OR "osteoarthroses"[tiab] OR "arthrosis"[tiab] OR "arthroses"[tiab] OR "arthritic spine*"[tiab] OR "osteoarthritic spine*"[tiab] OR "osteo-arthritic spine*"[tiab] OR "arthritic knee*"[tiab] OR "osteoarthritic knee*"[tiab] OR "osteo-arthritic knee*"[tiab] OR "arthritic hip"[tiab] OR "arthritic hips"[tiab] OR "osteoarthritic hip"[tiab] OR "osteoarthritic hips"[tiab] OR "osteo-arthritic hip"[tiab] OR "osteo-arthritic hips"[tiab] OR "Arthritis"[Mesh:NoExp] OR arthritis[tiab] OR arthritides[tiab] OR **monarthritis[tiab]** OR Polyarthritis[tiab] OR Polyarthritides[tiab] OR "Arthritis, Rheumatoid"[Mesh:NoExp] OR "Spondylitis"[Mesh:NoExp] OR spondylitis[tiab] OR "Spinal Curvatures"[Mesh] OR "spinal curv*"[tiab] OR kyphosis[tiab] OR kyphoses[tiab] OR hyperkyphosis[tiab] OR hyperkyphoses[tiab] OR scheuermann*[tiab] OR lordosis[tiab] OR lordoses[tiab] OR hyperlordosis[tiab] OR hyperlordoses[tiab] OR scoliosis[tiab] OR scolioses[tiab] OR "Spondylosis"[Mesh] OR spondylosis[tiab] OR spondylolysis[tiab] OR spondylolisthesis[tiab] OR Back[tiab] OR "Pelvic girdle*"[tiab] OR Neck[tiab] OR Cervical[tiab] OR Shoulder*[tiab] OR Elbow*[tiab] OR Wrist*[tiab] OR Hand[tiab] OR Hands[tiab] OR Hip[tiab] OR Hips[tiab] OR Knee[tiab] OR Knees[tiab] OR Ankle*[tiab] OR Foot[tiab] OR Feet[tiab] OR discogenic[tiab] OR Joint*[tiab] OR Metatarsophalangeal*[tiab] OR Spine*[tiab] OR Spinal*[tiab] OR Vertebra*[tiab] OR "articulationes pedis"[tiab] OR "cervical atlas"[tiab] OR "intervertebral dis*"[tiab] OR "annulus fibrosus"[tiab] OR "nucleus pulposus"[tiab] OR "Elbow Joint"[Mesh] OR "Elbow"[Mesh] OR "Wrist Joint"[Mesh] OR "Wrist"[Mesh] OR "Hand"[Mesh] OR "Hand Joints"[Mesh:NoExp] OR "Hip Joint"[Mesh] OR "Hip"[Mesh] OR "Knee Joint"[Mesh] OR "Knee"[Mesh] OR "Foot"[Mesh:NoExp] OR "Foot Joints"[Mesh:NoExp] OR "foot joint*"[tiab] OR "Ankle Joint"[Mesh] OR "Ankle"[Mesh] OR "Metatarsophalangeal Joint"[Mesh] OR "Spine"[Mesh:NoExp] OR "Cervical Vertebrae"[Mesh] OR "Intervertebral Disc"[Mesh] OR "Lumbar Vertebrae"[Mesh] OR "Upper Extremity"[Mesh:NoExp] OR "upper extremit*"[tiab] OR "upper limb*"[tiab] OR "Lower Extremity”[Mesh:NoExp] OR "lower extremit*"[tiab] OR "lower limb*"[tiab])) NOT medline[sb]

I1: Exercise therapy

("Resistance Training"[Mesh] OR "resistance training"[tiab] OR "strength training"[tiab] OR "weight lifting"[tiab] OR "Muscle Stretching Exercises"[Mesh] OR "muscle stretch*"[tiab] OR "balance train*"[tiab] OR "Exercise"[Mesh:NoExp] OR exercis*[tiab] OR "Exercise Therapy"[Mesh:NoExp] OR "Physical Therapy Specialty"[Mesh] OR "physical therapist*"[tiab] OR "Physical Therapy Modalities"[Mesh:NoExp] OR physiotherap*[tiab] OR (physical[tiab] AND (therapy[tiab] OR therapies[tiab] OR activity[tiab] OR activities[tiab])) OR "functional train*"[tiab] OR "gait train*"[tiab] OR "Exercise Movement Techniques"[Mesh:NoExp] OR "training intervention*"[tiab] OR "Breathing Exercises"[Mesh:NoExp] OR "breathing exercis*"[tiab] OR "breathing train*"[tiab] OR "breathing muscle exercis*"[tiab] OR "breathing muscle train*"[tiab] OR "respiratory exercise*"[tiab] OR "respiratory train*"[tiab] OR "respiratory muscle exercis*"[tiab] OR "respiratory muscle train*"[tiab] OR "pulmonary exercis*"[tiab] OR "pulmonary train*"[tiab] OR "pulmonary muscle exercis*"[tiab] OR "pulmonary muscle train"[tiab] OR "lung exercis*"[tiab] OR "lung train*"[tiab] OR "lung muscle exercis*"[tiab] OR "lung muscle train*"[tiab] OR "Rehabilitation"[Mesh:NoExp] OR "rehabilitation"[SH] OR rehabilitat*[tiab] OR habilitat*[tiab] OR convalescen*[tiab] OR "Recovery of Function"[Mesh] OR (recover*[tiab] AND function*[tiab]) OR refunction*[tiab] OR "re-function*"[tiab] OR "Hospitals, Rehabilitation"[Mesh] OR "Rehabilitation Centers"[Mesh:NoExp] OR "Occupational Therapy"[Mesh] OR (occupational[tiab] AND therap*[tiab]) OR "Telerehabilitation"[Mesh] OR "telerehabilitat*"[tiab] OR "tele-rehabilitat*"[tiab] OR ("daily activit*"[tiab] AND monitor*[tiab]) OR "individualized feedback*"[tiab] OR cue[tiab] OR cues[tiab] OR cueing[tiab] OR remind*[tiab] OR "Reminder Systems"[Mesh] OR texting[tiab] OR homework[tiab] OR "Telemedicine"[MeSH Terms] OR "Telenursing"[MeSH Terms] OR "User-Computer Interface"[MeSH Terms] OR "Multimedia"[MeSH Terms] OR "Cell phone"[MeSH Terms] OR "Public health informatics"[MeSH Terms] OR "Medical informatics"[MeSH Terms] OR "Nursing informatics"[MeSH Terms] OR "computers, handheld"[MeSH Terms] OR "Mobile Applications"[MeSH Terms] OR "Internet-Based Intervention"[Mesh] OR "Patient Portals"[MeSH Terms] OR "econsult*"[Title/Abstract] OR "e consult*"[Title/Abstract] OR "ediagnos*"[Title/Abstract] OR "e diagnos*"[Title/Abstract] OR "mobile health*"[Title/Abstract] OR "mhealth*"[Title/Abstract] OR "m health*"[Title/Abstract] OR "telehealth*"[Title/Abstract] OR "tele health"[Title/Abstract] OR "remote consult*"[Title/Abstract] OR "Telenursing"[Title/Abstract] OR "tele nursing"[Title/Abstract] OR "telediagnos*"[Title/Abstract] OR "tele diagnos*"[Title/Abstract] OR "telemedic*"[Title/Abstract] OR "tele medic*"[Title/Abstract] OR "telemonitor*"[Title/Abstract] OR "tele monitor*"[Title/Abstract] OR "remote monitor*"[tiab] OR "remote selfmonitor*"[tiab] OR "remote self-monitor*"[tiab] OR "ehealth*"[Title/Abstract] OR "e health*"[Title/Abstract] OR "telecare"[Title/Abstract] OR "tele care"[Title/Abstract] OR "digital health"[Title/Abstract] OR "app"[Title/Abstract] OR "apps"[Title/Abstract] OR "smartphone*"[Title/Abstract] OR "phone application*"[Title/Abstract] OR "telephone application*"[Title/Abstract] OR "mobile application*"[Title/Abstract] OR "portable software application*"[tiab] OR "portable electronic application*"[tiab] OR "mobile technolog*"[Title/Abstract] OR "health technolog*"[Title/Abstract] OR "health application*"[Title/Abstract] OR "internet*"[Title/Abstract] OR "web-based"[tiab] OR "web-oriented"[tiab] OR "world wide web*"[Title/Abstract] OR "webportal*"[Title/Abstract] OR "web portal*"[Title/Abstract] OR "patient portal*"[Title/Abstract] OR "ipad"[Title/Abstract] OR "ipads"[Title/Abstract] OR "sms"[Title/Abstract] OR "mms"[Title/Abstract] OR "text messag*"[Title/Abstract] OR "ussd"[Title/Abstract] OR "pda"[Title/Abstract] OR "laptop*"[Title/Abstract] OR "palmtop*"[Title/Abstract] OR "palm top*"[Title/Abstract] OR "personal digital assistant*"[Title/Abstract] OR "Distant counsel*"[tiab] OR "Distant consult*"[tiab] OR "Distant intervention*"[tiab] OR "Distant educat*"[tiab] OR "Distant learning*"[tiab] OR "distance counsel*"[tiab] OR "distance consult*"[tiab] OR "distance intervention*"[tiab] OR "distance educat*"[tiab] OR "distance learning*"[tiab] OR "online counsel*"[tiab] OR "online consult*"[tiab] OR "online intervention*"[tiab] OR "online educat*"[tiab] OR "online learning*"[tiab] OR "on-line counsel*"[tiab] OR "on-line consult*"[tiab] OR "on-line intervention*"[tiab] OR "on-line educat*"[tiab] OR "on-line learning*"[tiab] OR "remote counsel*"[tiab] OR "remote consult*"[tiab] OR "remote intervention*"[tiab] OR "remote educat*"[tiab] OR "remote learning*"[tiab] OR "mobile counsel*"[tiab] OR "mobile consult*"[tiab] OR "mobile intervention*"[tiab] OR "mobile educat*"[tiab] OR "mobile learning*"[tiab] OR "tele counsel*"[tiab] OR "tele consult*"[tiab] OR "tele intervention*"[tiab] OR "tele educat*"[tiab] OR "tele learning*"[tiab] OR "telecounsel*"[tiab] OR "teleconsult*"[tiab] OR "teleintervention*"[tiab] OR "teleeducat*"[tiab] OR "telelearning*"[tiab] OR "e-counsel*"[tiab] OR "e-consult*"[tiab] OR "e-intervention*"[tiab] OR "e-educat*"[tiab] OR "e-learning*"[tiab] OR ecounsel*[tiab] OR econsult*[tiab] OR eintervention*[tiab] OR eeducat*[tiab] OR elearning*[tiab] OR "patient monitoring"[Title/Abstract] OR "interactive voice response*"[Title/Abstract] OR "Multimedia"[Title/Abstract] OR "Mhapps"[Title/Abstract] OR "iphone*"[Title/Abstract] OR "android"[Title/Abstract] OR "game*"[Title/Abstract] OR "gaming"[Title/Abstract] OR "gamification"[Title/Abstract] OR "whatsapp*"[Title/Abstract] OR "e coach*"[Title/Abstract] OR "wearable*"[Title/Abstract] OR "exergam*"[Title/Abstract] OR "serious gam*"[Title/Abstract] OR "personal health record*"[Title/Abstract] OR "personal electronic health record*"[Title/Abstract] OR "health kiosk*"[Title/Abstract] OR "digital care"[tiab] OR "remote intervention*"[tiab] OR "remote consult*"[tiab] OR "Combined Modality Therapy"[Mesh:NoExp] OR "Combined Modality Therap*"[tiab] OR "Combined Modality treatment*"[tiab] OR "Combined Modality intervention*"[tiab] OR "multimodal therap*"[tiab] OR "multi-modal therap*"[tiab] OR "multimodal treatment*"[tiab] OR "multi-modal treatment*"[tiab] OR "Multimodal intervention*"[tiab] OR "Multi-modal intervention*"[tiab] OR "Education, Distance"[Mesh] OR "Distance Counseling"[Mesh]) NOT medline[sb]

I2: Motor Learning

("Motor Skills"[Mesh] OR "motor skill*"[tiab] OR "psychomotor skill*"[tiab] OR "motor control*"[tiab] OR "psychomotor control*"[tiab] OR "Psychomotor Performance"[Mesh:NoExp] OR "motor coordinat*"[tiab] OR "psychomotor coordination*"[tiab] OR "motor performance*"[tiab] OR "psychomotor performance*"[tiab] OR "Motor Activity"[Mesh] OR "motor activit*"[tiab] OR "psychomotor activit*"[tiab] OR imitat*[tiab] OR "motor imagery"[tiab] OR "movement imagery"[tiab] OR "mental practic*"[tiab] OR "Learning"[Mesh:NoExp] OR reflect*[tiab] OR "Imprinting, Psychological"[Mesh] OR "Transfer, Psychology"[Mesh] OR "learn*"[tiab] OR experien*[tiab] OR imprint*[tiab] OR "skill retention"[tiab] OR "skills retention"[tiab] OR (psychology[tiab] AND transfer[tiab]) OR Phenomenograph*[tiab] OR "memory training"[tiab] OR "Practice, Psychological"[Mesh] OR "psychological practic*"[tiab] OR "Reinforcement, Psychology"[Mesh:NoExp] OR "psychology reinforcement*"[tiab] OR "psychological reinforcement*"[tiab] OR "positive reinforcement*"[tiab] OR "Reinforcement, Verbal"[Mesh] OR "verbal reinforcement*"[tiab] OR "Metaphor"[Mesh] OR metaphor*[tiab] OR metafor*[tiab] OR "instruction of perform*"[tiab] OR "instruction of task*"[tiab] OR "attentional focus"[tiab] OR "focus of attention"[tiab] OR "Attention"[Mesh] OR "attention"[tiab] OR "internal focus"[tiab] OR "intrinsic focus"[tiab] OR "external focus"[tiab] OR "implicit instruction*"[tiab] OR "explicit instruction*"[tiab] OR "rhythmic auditory"[tiab]. OR RAS[tiab] OR "auditory rhythm*"[tiab] OR music[tiab] OR counting[tiab] OR "manual help*"[tiab] OR "manual correction*"[tiab] OR "Teaching"[Mesh:NoExp] OR teach*[tiab] OR instruct*[tiab] OR "verbal feedback*"[tiab] OR "verbal feed back*"[tiab] OR "audio feedback*"[tiab] OR "audio feed back*"[tiab] OR Reinforcement, Social[Mesh] OR "social reinforcement*"[tiab] OR "visual feedback*"[tiab] OR "visual feed back*"[tiab] OR "Mirror Movement Therapy"[Mesh] OR "mirror feedback*"[tiab] OR "mirror feed back*"[tiab] OR "mirror movement therap*"[tiab] OR "mirror therap*"[tiab] OR "auditory feedback*"[tiab] OR "auditory feed back*"[tiab] OR "tactile feedback*"[tiab] OR "tactile feed back*"[tiab] OR photo[tiab] OR smartphone*[tiab] OR video*[tiab] OR "coaches' eye*"[tiab] OR coach's eye*[tiab] OR "Biofeedback, Psychology"[Mesh:NoExp] OR biofeedback*[tiab] OR "bio-feedback*"[tiab] OR "biofeed back*"[tiab] OR "bio-feed back*"[tiab] OR "Feedback, Sensory"[Mesh] OR "sensory feedback*"[tiab] OR "sensory feed back*"[tiab] OR "proprioceptive feedback*"[tiab] OR "proprioceptive feed back*"[tiab] OR "sensorimotor feedback*"[tiab] OR "sensorimotor feed back*"[tiab] OR "sensorimotor function*"[tiab] OR "sensori-motor function*"[tiab] OR "sensorimotor integration*"[tiab] OR "sensori-motor integration*"[tiab] OR "Proprioception"[Mesh] OR proprioception[tiab] OR "vestibular sense*"[tiab] OR "sense of equilibrium"[tiab] OR "equilibrium sense*"[tiab] OR "labyrinthine sense*"[tiab] OR "position sense*"[tiab] OR "posture sense*"[tiab] OR "sense of position*"[tiab] OR kinesth*[tiab] OR ((postur*[tiab] OR musculoskeletal[tiab] OR muskuloskeletal[tiab] OR "musculo-skeletal"[tiab] OR "muskulo-skeletal"[tiab]) AND (equilibri*[tiab] OR balance*[tiab])) OR "core stabilit*"[tiab] OR "core balanc*"[tiab] OR "internal feedback*"[tiab] OR "internal feed back*"[tiab] OR "intrinsic feedback*"[tiab] OR "intrinsic feed back*"[tiab] OR "extrinsic feedback*"[tiab] OR "extrinsic feed back*"[tiab] OR "augmented feedback*"[tiab] OR "augmented feed back*"[tiab] OR "positive feedback*"[tiab] OR "positive feed back*"[tiab] OR "psychological feedback*"[tiab] OR "psychological feed back*"[tiab] OR "knowledge of perform*"[tiab] OR "knowledge of result*"[tiab] OR "Knowledge of Results, Psychological"[Mesh] OR KP [tiab] OR KR [tiab] OR "feedback during perform*"[tiab] OR "feedback after perform*"[tiab] OR ("feedback*"[tiab] AND "frequenc*"[tiab]) OR "Feedback"[Mesh] OR "feedforward*"[tiab] OR "feed forward*"[tiab] OR "Cues"[Mesh] OR (cue[tiab] OR cues[tiab] OR cueing[tiab]) OR "Environment"[Mesh] OR "unstable environment*"[tiab] OR "open environment*"[tiab] OR "protective environment*"[tiab] OR "static environment*"[tiab] OR "predictable environment*"[tiab] OR "simple environment*"[tiab] OR "complex environment*"[tiab] OR "variable environment*"[tiab] OR "busy environment*"[tiab] OR "whole task*"[tiab] OR "part task*"[tiab] OR "subdivide task*"[tiab] OR "interleaved practice*"[tiab] OR "blocked practice*"[tiab] OR "random practice*"[tiab] OR "distributed practice*"[tiab] OR "massed practice*"[tiab] OR "organisation of practice*"[tiab] OR "organization of practice*"[tiab]) NOT medline[sb]

I3: Behavioural change techniques

("behavioural chang*"[tiab] OR "behavioral chang*"[tiab] OR "behaviour chang*"[tiab] OR "behavior chang*"[tiab] OR "Goals"[Mesh] OR goal*[tiab] OR "Motivation"[Mesh:NoExp] OR motivation[tiab] OR "Action plan*"[tiab] OR "Transition plan*"[tiab] OR Commit*[tiab] OR "Problem Solving"[Mesh:NoExp] OR (problem*[tiab] AND solv*[tiab]) OR "Decision Making, Shared"[Mesh] OR "shared decision*"[tiab] OR "Patient Participation"[Mesh] OR "patient participation"[tiab] OR "patient involv*"[tiab] OR "patient engage*"[tiab] OR "patient activat*"[tiab] OR "implementation intention*"[tiab] OR (conservat*[tiab] AND ("mental resources"[tiab] OR "mental effort"[tiab])) OR "Mental Fatigue"[Mesh:NoExp] OR "mental fatigue"[tiab] OR "monitoring behavio*"[tiab] OR "self monitor*"[tiab] OR "selfmonitor*"[tiab] OR "Social Support"[Mesh] OR "social support*"[tiab] OR "social care"[tiab] OR "social approval*"[tiab] OR "social comparison*"[tiab] OR "psychological support*"[tiab] OR " psychological care"[tiab] OR " psychological approval*"[tiab] OR "psychological comparison*"[tiab] OR "psychosocial support*"[tiab] OR "psychosocial care"[tiab] OR "psychosocial approval*"[tiab] OR "psychosocial comparison*"[tiab] OR "psycho-social support*"[tiab] OR "psycho-social care"[tiab] OR "psycho-social approval*"[tiab] OR "psycho-social comparison*"[tiab] OR "community support"[tiab] OR "community care"[tiab] OR "community approval*"[tiab] OR "community comparison*"[tiab] OR "emotional support"[tiab] OR "emotional care"[tiab] OR "emotional approval*"[tiab] OR "emotional comparison*"[tiab] OR "support system*"[tiab] OR "Social Comparison"[Mesh] OR "social comparison"[tiab] OR antecedent*[tiab] OR "re-attribution*"[tiab] OR "health consequence*"[tiab] OR "social consequence*"[tiab] OR "emotional consequence*"[tiab] OR "anticipated regret*"[tiab] OR "future punishment*"[tiab] OR behavior substitution*[tiab] OR "behaviour substitution*"[tiab] OR "habit formation*"[tiab] OR "habit reversal*"[tiab] OR "Generalization, Psychological"[Mesh] OR generalisation*[tiab] OR generalization*[tiab] OR "graded task*"[tiab] OR "graded activit*"[tiab] OR "graded exercis*"[tiab] OR "graded therap*"[tiab] OR "Reward"[Mesh] OR reward*[tiab] OR "self-incentive*"[tiab] OR "restructuring environment*"[tiab] OR "environmental restructuring*"[tiab] OR "changing environment*"[tiab] OR "environmental chang*"[tiab] OR "Cognitive Restructuring"[Mesh] OR "cognitive restructur*"[tiab] OR "cognitive reapprais*"[tiab] OR refram*[tiab] OR "self ident*"[tiab] OR "changing cognition*"[tiab] OR "cognition chang*"[tiab] OR "changing emotion*"[tiab] OR "emotion chang*"[tiab] OR "emotional chang*"[tiab] OR "Emotional Regulation"[Mesh] OR (emotion*[tiab] AND regulat*[tiab]) OR "shifting perspectiv*"[tiab] OR "valued self-identit*"[tiab] OR "mental rehears*"[tiab] OR "past succes*"[tiab] OR "pain belief*"[tiab] OR "Treatment Adherence and Compliance"[Mesh:NoExp] OR Patient Compliance[Mesh:NoExp] OR adherence [tiab] OR complian*[tiab] OR "Self Efficacy"[Mesh] OR "self efficacy"[tiab] OR "Self concept*"[tiab] OR maintenance*[tiab] OR maintain*[tiab] OR relaps*[tiab] OR "Recurrence"[Mesh:NoExp] OR recurren*[tiab] OR exacerbation*[tiab] OR "Secondary Prevention"[Mesh] OR "secondary prevention*"[tiab] OR "secondary disease prevention*"[tiab] OR "secondary illness prevention*"[tiab] OR "secundary prevention*"[tiab] OR "secundary disease prevention*"[tiab] OR "secundary illness prevention*"[tiab] OR "Illness Behavior"[Mesh] OR "illness behavio*"[tiab] OR "sickness behavio*"[tiab] OR "Health Behavior"[Mesh:NoExp] OR "health behavio*"[tiab] OR "health-related behavio*"[tiab] OR "Attitude to Health"[Mesh:NoExp] OR "Risk Reduction Behavior"[Mesh:NoExp] OR "Risk reduction*"[tiab] OR "Attitude"[Mesh:NoExp] OR attitud*[tiab] ) NOT medline[sb]

I4: Selfmanagement support

("Self-Management"[Mesh] OR "self-manage*"[tiab] OR selfmanage*[tiab] OR "Self-Control"[Mesh] OR "self-control*"[tiab] OR selfcontrol*[tiab] OR selfregulation*[tiab] OR "self-regulation*"[tiab] OR "emotional regulation*"[tiab] OR "Self Care"[Mesh:NoExp] OR "self care"[tiab] OR "self caring"[tiab] OR "Self-Help Groups"[Mesh] OR "self help"[tiab] OR "Empowerment"[Mesh] OR empower*[tiab] OR "Patient Education as Topic"[Mesh] OR "patient educat*"[tiab] OR "patient train*"[tiab] OR "Health Education"[Mesh:NoExp] OR "health education"[tiab] OR "Consumer Health Information"[Mesh] OR "health information"[tiab] OR "health literacy"[tiab] OR "health illiteracy"[tiab] OR "medical literacy"[tiab] OR "health knowledge"[tiab] OR "health comprehen*"[tiab] OR "health skill*"[tiab] OR "health attitude*"[tiab] OR "Teach-Back Communication"[Mesh] OR "teach-back"[tiab] OR "Education"[Mesh:NoExp] OR diary[tiab] OR diaries[tiab] OR "Patient Education Handout"[Publication Type] OR handout*[tiab] OR "hand-out*"[tiab] OR "Teaching Materials"[Mesh:NoExp] OR "teaching material*"[tiab] OR "Books"[Mesh] OR book*[tiab] OR "Medical Illustration"[Mesh] OR "medical illustration*"[tiab] OR leaflet*[tiab] OR "Video-Audio Media"[Publication Type] OR video*[tiab] OR "audiovisual"[tiab] OR "audio-visual"[tiab] OR film[tiab] OR films[tiab] OR tutorial*[tiab] OR webcast*[tiab] OR podcast*[tiab] OR youtube*[tiab] OR inform*[tiab] OR "Knowledge"[Mesh] OR "knowledge of disease*"[tiab] OR "Attitude to Health"[Mesh:NoExp] OR "Mentoring"[Mesh] OR mentor*[tiab] OR "pros and cons"[tiab] OR "decisional balanc*"[tiab] OR "comparative imag*"[tiab] OR "Directive counseling"[Mesh] OR "motivational interview*"[tiab] OR "body awareness"[tiab] OR "active listening"[tiab] OR "relaxation techni*"[tiab] OR "pain provocation*"[tiab] OR "Adaptation, Psychological"[Mesh:NoExp] OR "psychological adaptation*"[tiab] OR "occupational adaptation*"[tiab] OR "work adaptation*"[tiab] OR "job adaptation*"[tiab] OR "psychological adjustment*"[tiab] OR "occupational adjustment*"[tiab] OR "job adjustment*"[tiab] OR "adaptive behavio*"[tiab] OR coping[tiab] OR "stress management"[tiab] OR "support seeking"[tiab] OR regulation[tiab] OR "Life Style"[Mesh:NoExp] OR "life style*"[tiab] OR "lifestyle*"[tiab] OR Cognitive Behavioral Therapy[Mesh:NoExp] OR ("cognitive behavio*"[tiab] AND (therap*[tiab] OR treat*[tiab])) OR CBT [tiab] OR "Health Belief Model"[Mesh] OR "health belief*"[tiab] OR "transtheoretical model*"[tiab] OR "trans-theoretical model*"[tiab] OR "transactional model*"[tiab] OR "trans-actional model*"[tiab] OR TTM[tiab] OR "Models, Biopsychosocial"[Mesh] OR "biopsychosocial model*"[tiab] OR "bio-psychosocial model*"[tiab] OR "behavior theor*"[tiab] OR "behaviour theor*"[tiab] OR "stages of change*"[tiab] OR "change stage*"[tiab] OR prochaska*[tiab] OR "pain belief*"[tiab] OR "Internal-External Control"[Mesh] OR "internal-external control*"[tiab] OR "external-internal control*"[tiab] OR "sense of control*"[tiab] OR "locus of control*"[tiab] OR "Health Promotion"[Mesh:NoExp] OR "health promotion"[tiab] OR "attitude towards illness*"[tiab] OR "attitude towards disease*"[tiab] OR "attitude towards disorder*"[tiab] OR "attitude to health"[tiab] OR "common sense model*"[tiab] OR Huber*[tiab] OR "positive health"[tiab] OR "Stress theory"[tiab] OR "Social Cognitive theory"[tiab] OR "Social Learning theory"[tiab] OR "Theory of Planned Behavior"[tiab] OR "Theory of Planned Behaviour"[tiab] OR "Planned behavior theory"[tiab] OR "Planned Behaviour theory"[tiab] OR "Reasoned Action Theory"[tiab]) NOT medline[sb]

O1: Movement behaviour

("Movement"[Mesh] OR "movement*"[tiab] OR coordination*[tiab] OR compensat*[tiab] OR "co-contraction"[tiab] OR "cocontraction"[tiab] OR "Muscle Tonus"[Mesh] OR "tonus muscle*"[tiab] OR "muscle tonus*"[tiab] OR "muscle tension*"[tiab] OR "muscle tightness*"[tiab] OR "muscular tension*"[tiab] OR agonist*[tiab] OR antagonist*[tiab] OR "Muscle Contraction"[Mesh] OR "muscle contraction*"[tiab] OR "muscular contraction*"[tiab] OR inotropism*[tiab] OR "Muscle Relaxation"[Mesh] OR "muscle relaxation*"[tiab] OR "muscular relaxation*"[tiab] OR "muscle function*"[tiab] OR "muscular function*"[tiab] OR "Task Performance and Analysis"[Mesh:NoExp] OR "task performanc*"[tiab] OR "Work Performance"[Mesh] OR "work performanc*"[tiab] OR "job performanc*"[tiab] OR "occupational performanc*"[tiab] OR "task analy*"[tiab] OR "workload measurement*"[tiab] OR "critical incident techn*"[tiab] OR "Postural Balance"[Mesh] OR "postural control*"[tiab] OR "postural equilibri*"[tiab] OR "core stabilit*"[tiab] OR balanc*[tiab] OR unbalanc*[tiab] OR align*[tiab] OR "Ergonomics" [Mesh:NoExp] OR ergonomic*[tiab] OR "Environment" [Mesh:NoExp] OR environment*[tiab] OR

(physical[tiab] AND time[tiab]) OR "sedentary time"[tiab] OR "Time Factors"[Mesh] OR "minutes per week"[tiab] OR "minutes per day"[tiab] OR bout*[tiab] OR "Sedentary Behavior"[Mesh] OR "sedentary behavio*"[tiab] OR "sedentary lifestyle*"[tiab] OR "sedentary life style*"[tiab] OR "physical inactivit*"[tiab] OR "lack of physical activit*"[tiab] OR "Heart Rate"[Mesh:NoExp] OR "heart rate*"[tiab] OR "cardiac rate*"[tiab] OR "pulse rate*"[tiab] OR "heart beat*"[tiab] OR HRR[tiab] OR "Metabolic Equivalent"[Mesh] OR MET[tiab] OR "metabolic equivalent"[tiab] OR "light intensit*"[tiab] OR "moderate intensit*"[tiab] OR "high intensit*"[tiab] OR "vigorous intensit*"[tiab] OR " physical activit*"[tiab] OR "functional activit*"[tiab] OR pace[tiab] OR paces[tiab] OR "Walking Speed"[Mesh] OR speed*[tiab] OR velocity[tiab] OR "Acceleration"[Mesh] OR kinematics[tiab] OR accelerat*[tiab] OR "Range of Motion, Articular"[Mesh] OR "range of motion*"[tiab] OR ROM[tiab] OR (flexibilit*[tiab] AND joint*[tiab]) OR "Physical Functional Performance"[Mesh:NoExp] OR (functional[tiab] AND performanc*[tiab]) OR (physical[tiab] AND performanc*[tiab]) ) NOT medline[sb]

O2: Activities of daily living

(("Mobility Limitation"[Mesh] OR "mobility limitation*"[tiab] OR "Dependent Ambulation"[Mesh] OR disabilit*[tiab] OR "International Classification of Functioning, Disability and Health"[Mesh] OR ICF[tiab] OR "Activities of Daily Living"[Mesh:NoExp] OR "daily activit*"[tiab] OR "daily life"[tiab] OR "daily living"[tiab] OR "daily lives"[tiab] OR ADL[tiab] OR everyday[tiab] OR "every day"[tiab] OR "Functional Status"[Mesh] OR "functional status"[tiab] OR "functional dependen*"[tiab] OR "functional independen*"[tiab] OR "Work"[Mesh:NoExp] OR "Return to work"[Mesh] OR "Work-Life Balance"[Mesh] OR work*[tiab] OR job[tiab] OR jobs[tiab] OR Commut*[tiab] OR sport*[tiab] OR leisure*[tiab] OR hobb*[tiab] OR "Home Environment"[Mesh] OR home*[tiab] OR "living alone"[tiab] OR "Schools"[Mesh] OR school*[tiab] OR "Students"[Mesh:NoExp] OR student* [tiab] OR "Child Care"[Mesh] OR "child care"[tiab] OR "day care"[tiab] OR puericulture[tiab] OR "informal care"[tiab] OR caregiv*[tiab] OR "care giv*"[tiab] OR "Volunteers"[Mesh] OR volunteer*[tiab] OR "Social Participation"[Mesh] OR "social participati*"[tiab] OR "socially participati*"[tiab] OR "social involv*"[tiab] OR "socially involv*"[tiab] OR "social engag*"[tiab] OR "socially engag*"[tiab] OR "sports participati*"[tiab] OR "sport participati*"[tiab] OR "sports involv*"[tiab] OR "sport involv*"[tiab] OR "sports engag*"[tiab] OR "sport engag*"[tiab]) OR (("Posture"[Mesh:NoExp] OR postur*[tiab] OR "Standing Position"[Mesh] OR stand[tiab] OR standing[tiab] OR "Sitting Position"[Mesh] OR sit[tiab] OR sitting[tiab] OR "seated"[tiab] OR recumben*[tiab] OR lying[tiab] OR "Prone Position"[Mesh] OR "prone position*"[tiab] OR "Supine Position"[Mesh] OR "supine position"[tiab] OR ambulat*[tiab] OR "Gait"[Mesh:NoExp] OR gait*[tiab] OR "Walking"[Mesh:NoExp] OR walk*[tiab] OR step*[tiab] OR stoop*[tiab] OR bend*[tiab] OR squat*[tiab] OR push*[tiab] OR pull*[tiab] OR "golf ball techn*"[tiab] OR "golf techn*"[tiab] OR "golfers techn*"[tiab] OR "weight lifting technique*"[tiab] OR wlt[tiab] OR "Lifting"[Mesh] OR "lift*"[tiab] OR "carry*"[tiab] OR jump*[tiab] OR "Stair Climbing"[Mesh] OR "stair climb*"[tiab] OR "stair navigat*"[tiab] OR rising[tiab] OR rise[tiab] OR "sit-to-stand"[tiab] OR "stand-to-sit"[tiab] OR "chair-to-bed"[tiab] OR "bed-to-chair"[tiab] OR reach*[tiab] OR throw*[tiab] OR "Locomotion"[Mesh:NoExp] OR locomot*[tiab] OR "Running"[Mesh] OR run[tiab] OR running[tiab] OR jog[tiab] OR jogging[tiab] OR "Head Movements"[Mesh] OR "head movement*"[tiab]) )) NOT medline[sb]

FILTERS

AND ("Controlled Clinical Trial"[PT] OR "Random Allocation"[Mesh] OR "Double-Blind Method"[Mesh] OR "Single-Blind Method"[mesh] OR rct[tiab] OR random*[tiab] OR trial*[tiab] OR "single-blind*"[tiab] OR "single-masked"[tiab] OR "double-blind*"[tiab] OR "double masked"[tiab] OR "triple-blind*"[tiab] OR "triple-masked"[tiab] OR "quasi-experimental"[tiab] OR "pre-test"[tiab] OR "pretest"[tiab] OR "post-test"[tiab] OR "posttest"[tiab])

NOT protocol[ti]
