# Supplementary material for: Evaluating the Effectiveness and Technique Composition of Combined Motor Learning and Behavior Change Strategies for Movement Behavior in Chronic Musculoskeletal Pain: A Systematic Review
Source: Arch Rehabil Res Clin Transl. 2026 Mar 24;8(3):100618. doi: 10.1016/j.arrct.2026.100618 (PMC13591100; doi:10.1016/j.arrct.2026.100618)
Supplement: Supplementary file 2 [file mmc2.docx]

Supplement 2. Search in Embase

Patiënt

(('Chronic pain'/de OR (((chronic OR persist* OR recurren* OR 'long-standing' OR ongoing OR 'on-going' OR continu* OR prolong*) NEAR/4 (Pain* OR Ache* OR complain*)):ti,ab,kw)) OR ('Musculoskeletal disease'/de OR msd:ti,ab,kw OR 'Musculoskeletal pain'/de OR

((musculoskeletal OR muskuloskeletal OR 'musculo-skeletal' OR 'muskulo-skeletal' OR muscle*) NEAR/4 (Disease* OR Pain* OR ache* OR condition* OR Disorder* OR problem* OR soreness* OR tenderness*)):ti,ab,kw OR (chronic NEAR/4 (Disease* OR condition* OR Disorder* OR problem* OR soreness* OR tenderness*)):ti,ab,kw OR 'arthralgia'/de OR arthralgia:ti,ab,kw OR 'Myalgia'/de OR myalgia:ti,ab,kw OR 'Pelvic girdle pain'/de

OR 'piriformis syndrome'/de OR 'Backache'/de OR 'Low back pain'/exp OR 'discogenic pain'/exp OR 'backpain*':ti,ab,kw OR 'backache*':ti,ab,kw OR lumbago*:ti,ab,kw OR LBP:ti,ab,kw OR 'sciatica'/de OR Sciatica:ti,ab,kw OR 'Neck pain'/exp OR 'neckpain*':ti,ab,kw OR 'neckache*':ti,ab,kw OR cervicalgia*:ti,ab,kw OR cervicodynia*:ti,ab,kw OR 'Whiplash injury'/de OR whiplash*:ti,ab,kw OR 'acceleration cranial trauma*':ti,ab,kw OR 'deceleration cranial trauma':ti,ab,kw OR ADT:ti,ab,kw OR 'Shoulder impingement syndrome'/de OR ((Shoulder OR Rotator OR Cuff OR Coracohumeral OR 'Coraco-humeral' OR Coracoid OR Subacromial OR 'Sub-acromial' OR Outlet OR Internal OR Glenoid*) NEAR/4 Impingement*):ti,ab,kw OR 'Bursitis'/de OR bursitis:ti,ab,kw OR bursitides:ti,ab,kw OR 'frozen shoulder*':ti,ab,kw OR capsulitis:ti,ab,kw OR capsulitides:ti,ab,kw OR 'shoulderpain*':ti,ab,kw OR 'shoulderache*':ti,ab,kw OR 'epicondylitis'/exp OR epicondylitis:ti,ab,kw OR epicondylitides:ti,ab,kw OR 'elbow tendinopath*':ti,ab,kw OR ((Tennis OR Golf*) NEAR/4 Elbow*):ti,ab,kw OR 'nerve compression'/de OR ((Nerve OR Neuropath*) NEAR/4 (Compression OR Entrapment*)):ti,ab,kw OR ((plexus OR nerve) NEAR/4 (impingement* OR entrapment*)):ti,ab,kw OR 'Carpal tunnel syndrome'/de OR 'carpal tunnel':ti,ab,kw OR 'Cubital tunnel syndrome'/de OR 'cubital tunnel':ti,ab,kw OR 'Radial neuropathy'/de OR (Radial NEAR/4 Neuropath*):ti,ab,kw OR ((Supinator OR 'Radial tunnel' OR 'Radial nerve') NEAR/4 (Syndrome* OR Disease*)):ti,ab,kw OR (Radial NEAR/4 Mononeuropath*):ti,ab,kw OR 'wrist-drop':ti,ab,kw OR 'Thorax outlet syndrome'/de OR (('thoracic outlet' OR 'thoracic aperture' OR 'thorax outlet' OR 'thorax aperture' OR Costoclavicular OR 'scalenus anticus' OR 'cervical rib' OR Naffziger*) NEAR/4 syndrome*):ti,ab,kw OR tos:ti,ab,kw OR 'complaints arm neck shoulder*':ti,ab,kw OR cans:ti,ab,kw OR 'Cauda equina syndrome'/de OR (('Cauda equina' OR Piriformis OR 'Tarsal tunnel') NEAR/4 Syndrome*):ti,ab,kw OR 'tarsal tunnel syndrome'/de OR 'intervertebral disk hernia'/exp OR 'tarsal tunnel syndrome'/de OR 'intervertebral disk hernia'/exp OR ((displacement* OR protru* OR hernia* OR slipped OR prolapse*) NEAR/4 (disk* OR disc*)):ti,ab,kw OR 'vertebral canal stenosis'/exp OR 'spinal stenosis':ti,ab,kw OR 'spinal stenoses':ti,ab,kw OR 'vertebral canal stenos*':ti,ab,kw OR 'chronic fatigue syndrome'/exp OR 'chronic fatigue':ti,ab,kw OR 'Fibromyalgia'/de OR fibromyalgi*:ti,ab,kw OR (Muscular NEAR/4 Rheuma*):ti,ab,kw OR fibrositis:ti,ab,kw OR fibrositides:ti,ab,kw OR 'myofascial pain':ti,ab,kw OR 'Osteoporosis'/de OR osteoporosis:ti,ab,kw OR osteoporoses:ti,ab,kw OR 'osteo-porosis':ti,ab,kw OR 'osteo-poroses':ti,ab,kw OR 'bone loss*':ti,ab,kw OR 'Spondylarthritis'/exp OR spondylarthritis:ti,ab,kw OR spondyloarthritis:ti,ab,kw OR spondylarthritides:ti,ab,kw OR spondyloarthritides:ti,ab,kw OR spondyloarthropath*:ti,ab,kw OR 'spondylo-arthropath*':ti,ab,kw OR spondylarthropath*:ti,ab,kw OR 'spondyl-arthropath*':ti,ab,kw OR 'Ankylosing spondylitis'/de OR Bechterew*:ti,ab,kw OR (Ankylo* NEAR/4 Spondyl*):ti,ab,kw OR 'Osteoarthritis'/de OR 'osteoarthritis':ti,ab,kw OR 'osteoarthritides':ti,ab,kw OR 'osteoarthrosis':ti,ab,kw OR 'osteoarthroses':ti,ab,kw OR 'arthrosis':ti,ab,kw OR 'arthroses':ti,ab,kw OR 'hand osteoarthritis'/de OR 'hip osteoarthritis'/de OR 'knee osteoarthritis'/de OR 'Spondylosis'/exp OR ((arthritic OR osteoarthritic OR 'osteo-arthritic') AND (Spine* OR Knee* OR Hip OR Hips)):ti,ab,kw OR 'Arthritis'/de OR arthritis:ti,ab,kw OR arthritides:ti,ab,kw OR 'chronic arthritis'/de OR 'Monarthritis'/exp OR monarthritis:ti,ab,kw OR 'Polyarthritis'/de OR Polyarthritis:ti,ab,kw OR Polyarthritides:ti,ab,kw OR 'Rheumatoid arthritis'/de OR 'Spondylitis'/de OR spondylitis:ti,ab,kw OR 'Spine disease'/de OR (Spinal NEAR/4 Curv*):ti,ab,kw OR 'kyphosis'/de OR kyphosis:ti,ab,kw OR kyphoses:ti,ab,kw OR hyperkyphosis:ti,ab,kw OR hyperkyphoses:ti,ab,kw OR 'Scheuermann disease'/de OR scheuermann*:ti,ab,kw OR 'lordosis'/de OR lordosis:ti,ab,kw OR lordoses:ti,ab,kw OR hyperlordosis:ti,ab,kw OR hyperlordoses:ti,ab,kw OR 'scoliosis'/de OR scoliosis:ti,ab,kw OR scolioses:ti,ab,kw OR 'spinal pain'/de OR 'spondylolisthesis'/de OR spondylolisthesis:ti,ab,kw OR 'spondylolysis'/de OR spondylolysis:ti,ab,kw OR 'spondylosis'/exp OR spondylosis:ti,ab,kw OR (Back OR 'Pelvic girdle' OR Neck OR Cervical OR Shoulder* OR Elbow* OR Wrist* OR Hand OR Hands OR Hip OR Hips OR Knee OR Knees OR Ankle* OR Foot OR Feet OR discogenic OR Joint* OR Metatarsophalangeal* OR Spine* OR Spinal* OR Vertebra*):ti,ab,kw OR (Articulationes NEAR/4 pedis):ti,ab,kw OR 'cervical atlas':ti,ab,kw OR (Intervertebral NEAR/4 dis*):ti,ab,kw OR 'annulus fibrosus':ti,ab,kw OR 'nucleus pulposus':ti,ab,kw OR 'Elbow'/exp OR 'Wrist'/exp OR 'Hand joint'/de OR 'Hand'/exp OR 'Foot'/de OR 'Hip'/exp OR 'Knee'/exp OR 'Ankle'/exp OR 'Spine'/de OR 'Metatarsophalangeal joint'/exp OR 'Cervical vertebra'/exp OR 'Intervertebral disk'/exp OR 'Lumbar vertebra'/exp OR 'Upper limb'/de OR 'Lower limb'/de OR ((Lower OR Upper) NEAR/4 (Extremit* OR Limb*)):ti,ab,kw))

I1: Exercise therapy

('Resistance training'/exp OR ((Resistance OR Strength*) NEAR/4 training*):ti,ab,kw OR 'weight lifting':ti,ab,kw OR 'Stretching exercise'/de OR (Muscle NEAR/4 Stretch*):ti,ab,kw OR 'balance training'/de OR (balance NEAR/4 train*):ti,ab,kw OR 'Exercise'/de OR exercis*:ti,ab,kw OR 'kinesiotherapy'/de OR 'physiotherapy'/de OR physiotherap*:ti,ab,kw OR (Physical NEAR/4 (Therap* OR Activit*)):ti,ab,kw OR ((funtional OR gait) N4 train*):ti,ab,kw OR 'training intervention*':ti,ab OR 'Breathing exercise'/de OR ((Breathing* OR Respirator* OR Pulmonary* OR Lung*) NEAR/4 (Train*)):ti,ab,kw OR 'Rehabilitation'/de OR rehabilitat*:ti,ab,kw OR habilitat*:ti,ab,kw OR 'Convalescence'/de OR Convalescence:ti,ab,kw OR (recover* NEAR/4 function*):ti,ab,kw OR refunction*:ti,ab,kw OR 're-function*':ti,ab,kw OR 'Rehabilitation center'/de OR 'Occupational therapy'/de OR (occupational NEAR/4 therap*):ti,ab,kw OR 'Telerehabilitation'/de OR 'telerehabilitat*':ti,ab,kw OR 'tele-rehabilitat*':ti,ab,kw OR (('Daily activities' OR 'Daily activity') NEAR Monitor*):ti,ab,kw OR (Individualized NEAR/4 Feedback*):ti,ab,kw OR cue:ti,ab,kw OR cues:ti,ab,kw OR cueing:ti,ab,kw OR remind*:ti,ab,kw OR 'Reminder system'/de OR texting:ti,ab,kw OR homework:ti,ab,kw OR 'Telemedicine'/de OR 'Telenursing'/de OR 'Computer interface'/de OR 'Multimedia'/de OR 'Mobile phone'/exp OR 'Medical informatics'/de OR 'Nursing informatics'/de OR 'Personal digital assistant'/de OR 'Mobile application'/exp OR 'Web-based intervention'/exp OR 'Patient portal'/de OR 'ediagnos*':ti,ab,kw OR 'e diagnos*':ti,ab,kw OR 'mobile health*':ti,ab,kw OR 'mhealth*':ti,ab,kw OR 'm health*':ti,ab,kw OR 'telehealth*':ti,ab,kw OR 'tele health':ti,ab,kw OR 'teleconsult*':ti,ab,kw OR 'Telenursing':ti,ab,kw OR 'tele nursing':ti,ab,kw OR 'telediagnos*':ti,ab,kw OR 'tele diagnos*':ti,ab,kw OR 'telemedic*':ti,ab,kw OR 'tele medic*':ti,ab,kw OR 'telemonitoring'/de OR 'telemonitor*':ti,ab,kw OR 'remote selfmonitor*':ti,ab,kw OR 'ehealth*':ti,ab,kw OR 'e health*':ti,ab,kw OR 'telecare':ti,ab,kw OR 'tele care':ti,ab,kw OR (Digital NEAR/4 Health):ti,ab,kw OR 'app':ti,ab,kw OR 'apps':ti,ab,kw OR 'smartphone*':ti,ab,kw OR ((Phone* OR Telephone* OR Mobile* OR Health OR portable) NEAR/4 (Application* OR Technolog*)):ti,ab,kw OR 'internet*':ti,ab,kw OR 'web-based':ti,ab,kw OR 'web-oriented':ti,ab,kw OR 'world wide web*':ti,ab,kw OR 'webportal*':ti,ab,kw OR 'web portal*':ti,ab,kw OR 'patient portal*':ti,ab,kw OR 'ipad':ti,ab,kw OR 'ipads':ti,ab,kw OR 'sms':ti,ab,kw OR 'mms':ti,ab,kw OR 'text messag*':ti,ab,kw OR 'ussd':ti,ab,kw OR 'pda':ti,ab,kw OR 'laptop*':ti,ab,kw OR 'palmtop*':ti,ab,kw OR 'palm top*':ti,ab,kw OR 'personal digital assistant*':ti,ab,kw OR 'telecounsel*':ti,ab,kw OR ((Distan* OR Online OR 'On-line' OR Remote OR mobile OR tele OR "e-") NEAR/4 (Counsel* OR Consult* OR Intervention* OR Educat* OR Learning* OR monitor*)):ti,ab,kw OR ecounsel*:ti,ab,kw OR econsult*:ti,ab,kw OR eintervention*:ti,ab,kw OR eeducat*:ti,ab,kw OR elearning*:ti,ab,kw OR 'patient monitoring':ti,ab,kw OR 'interactive voice response*':ti,ab,kw OR 'Multimedia':ti,ab,kw OR 'Mhapps':ti,ab,kw OR 'iphone*':ti,ab,kw OR 'android':ti,ab,kw OR 'game*':ti,ab,kw OR 'gaming':ti,ab,kw OR 'gamification':ti,ab,kw OR 'whatsapp*':ti,ab,kw OR 'e coach*':ti,ab,kw OR 'wearable*':ti,ab,kw OR 'exergam*':ti,ab,kw OR 'serious gam*':ti,ab,kw OR (Personal NEAR/4 Health NEAR/4 Record*):ti,ab,kw OR 'health kiosk*':ti,ab,kw OR 'digital care':ti,ab,kw OR 'Multimodal intervention'/exp OR ((Multimodal OR 'Multi-modal' OR 'Combined modality') NEAR/4 (Therap* OR Treatment* OR Intervention*)):ti,ab,kw OR 'Distance learning'/de OR 'mobile learning'/de OR 'e-counseling'/de )

I2: Motor Learning

('motor learning'/de OR 'motor control'/de OR 'motor coordination'/de OR 'Motor performance'/de OR ((psychomotor OR motor) NEAR/4 (control* OR coordinat* OR perform* OR skill* OR activit*)):ti,ab,kw OR 'Motor activity'/de OR 'motor activit*':ti,ab,kw OR imitat*:ti,ab,kw OR ((Motor OR Movement*) NEAR/4 Imagery):ti,ab,kw OR (Mental NEAR/4 Practic*):ti,ab,kw OR 'Learning'/de OR 'experience'/de OR 'reflection'/de OR reflect*:ti,ab,kw OR 'experiential learning'/de OR 'imitation'/de OR 'imprinting (psychology)'/de OR 'skill retention'/de OR 'transfer of learning'/exp OR 'errorless learning'/exp OR 'mental practice'/exp OR 'learn*':ti,ab,kw OR experien*:ti,ab,kw OR imprint*:ti,ab,kw OR (skill* NEAR/4 retention):ti,ab,kw OR (psychology NEAR/4 transfer*):ti,ab,kw OR Phenomenograph*:ti,ab,kw OR (Memory NEAR/4 Training):ti,ab,kw OR 'Psychological practice'/de OR (Psychological NEAR/4 Practic*):ti,ab,kw OR 'Reinforcement (psychology)'/de OR ((Psycholog* OR Positive OR Verbal* OR Social) NEAR/4 Reinforcement*):ti,ab,kw OR 'Verbal reinforcement'/de OR 'positive reinforcement'/de OR metaphor*:ti,ab,kw OR metafor*:ti,ab,kw OR (Instruction NEAR/4 (Perform* OR Task*)):ti,ab,kw OR (Attentional NEAR/4 Focus):ti,ab,kw OR 'Attention'/de OR 'attention':ti,ab,kw OR ((Internal* OR Intrinsic* OR External*) NEAR/4 Focus):ti,ab,kw OR (Implicit OR Explicit NEAR/4 Instruction*):ti,ab,kw OR 'rhythmic auditory stimulation'/exp OR (Rhythmic NEAR/4 Auditor*):ti,ab,kw OR RAS:ti,ab,kw OR music:ti,ab,kw OR counting:ti,ab,kw OR (Manual NEAR/4 (Help* OR Correction*)):ti,ab,kw OR 'Teaching'/de OR teach*:ti,ab,kw OR instruct*:ti,ab,kw OR ((Verbal OR Audio OR Auditory OR Tactile OR Visual* OR Mirror* OR Sensory OR Proprioceptive OR Sensorimotor OR Augmented OR Intrinsic* OR Extrinsic* OR Internal* OR External* OR Perform* OR Frequenc* OR positive OR psychological) NEAR/4 (Feedback* OR 'Feed back*')):ti,ab,kw OR 'Sensorimotor function'/de OR 'sensorimotor integration'/de OR ((Sensorimotor OR Sensori-motor) NEAR/4 (Function* OR integration*)):ti,ab,kw OR 'Social reinforcement'/de OR 'mirror therapy'/de OR (mirror* NEAR/4 therap*):ti,ab,kw OR photo:ti,ab,kw OR smartphone*:ti,ab,kw OR video*:ti,ab,kw OR (Coach* NEAR/4 Eye*):ti,ab,kw OR 'Biofeedback'/exp OR biofeedback*:ti,ab,kw OR 'bio-feedback*':ti,ab,kw OR 'biofeed back*':ti,ab,kw OR 'bio-feed back*':ti,ab,kw OR 'Sensory feedback'/exp OR 'Proprioception'/de OR proprioception:ti,ab,kw OR ((Vestibular OR Equilibrium OR Labyrinthine OR Position OR Posture) NEAR/4 Sense*):ti,ab,kw OR kinesth*:ti,ab,kw OR ((postur* OR musculoskeletal OR muskuloskeletal OR 'musculo-skeletal' OR 'muskulo-skeletal') NEAR/4 (equilibri* OR balance*)) OR (Core NEAR/4 (Stabilit* OR Balanc*)):ti,ab,kw OR (Knowledge NEAR/4 (Perform* OR Result*)):ti,ab,kw OR KP:ti,ab,kw OR KR:ti,ab,kw OR 'Feedback system'/de OR 'positive feedback'/de OR 'psychological feedback'/de OR 'feedforward*':ti,ab,kw OR (Feed NEAR/4 Forward*):ti,ab,kw OR cue:ti,ab,kw OR cues:ti,ab,kw OR cueing:ti,ab,kw OR 'environment'/de OR ((Unstable OR Open OR Protective OR Static OR Predictable OR Simple OR Complex OR Variable OR Busy) NEAR/4 Environment*):ti,ab,kw OR 'whole task*':ti,ab,kw OR 'part task*':ti,ab,kw OR 'subdivide task*':ti,ab,kw OR ((Interleaved OR Blocked OR Random OR Distributed OR Massed OR Organisation* OR Organization*) NEAR/4 Practice*):ti,ab,kw)

I3: Behavioural change techniques

( 'Behavior change'/de OR ((Behavior* OR Behaviour*) NEAR/4 (Change*)):ti,ab,kw OR 'goal setting'/exp OR Goal*:ti,ab,kw OR 'motivation'/de OR 'extrinsic motivation'/de OR 'intrinsic motivation'/de OR motivation:ti,ab,kw OR 'Action planning'/de OR ((Action OR transition) NEAR/4 Plan*):ti,ab,kw OR 'Commitment'/de OR 'Commit*':ti,ab,kw OR 'Problem solving'/de OR (Problem* NEAR/4 solv*):ti,ab,kw OR 'Shared decision making'/de OR (Shared NEAR/4 Decision*):ti,ab,kw OR 'Patient participation'/de OR (Patient NEAR/4 (Participati* OR Involv* OR Engag* OR Activiti*)):ti,ab,kw OR (Implementation NEAR/4 Intention*):ti,ab,kw OR (Conservat* NEAR/4 ('Mental Resourc*' OR 'Mental effort')):ti,ab,kw OR 'Mental fatigue'/de OR (Mental NEAR/4 Fatigue):ti,ab,kw OR (Monitor* NEAR/4 (Behavio* OR Self)):ti,ab,kw OR 'Self monitoring'/de OR 'selfmonitoring':ti,ab,kw OR 'Social support'/exp OR 'social comparison'/de OR ((Social OR psychological* OR psychosocial OR 'psycho-social' OR emotional* OR community) NEAR/4 (Support* OR Care OR Approv* OR Comparison*)):ti,ab,kw OR 'support system*':ti,ab,kw OR antecedent*:ti,ab,kw OR 're-attribution*':ti,ab,kw OR 'reattribution*':ti,ab,kw OR 'Consequences'/de OR ((Health OR Social OR Emotional) NEAR/4 Consequence*):ti,ab,kw OR (Anticipated NEAR/4 Regret*):ti,ab,kw OR (Future NEAR/4 Punishment*):ti,ab,kw OR ((Behavior OR Behaviour) NEAR/4 Substitution*):ti,ab,kw OR 'Habit reversal'/de OR (Habit NEAR/4 (Formation* OR Reversal)):ti,ab,kw OR 'Generalization (psychology)'/exp OR generalisation*:ti,ab,kw OR generalization*:ti,ab,kw OR 'Graded exercise therapy'/de OR (Graded NEAR/4 (Task* OR Activit* OR exercise* OR therap*)):ti,ab,kw OR 'Reward'/de OR reward*:ti,ab,kw OR 'self-incentive*':ti,ab,kw OR ((Restructur* OR Chang*) NEAR/4 Environment*):ti,ab,kw OR 'cognitive reappraisal'/de OR 'Cognitive restructuring'/de OR (Cognitive NEAR/4 (Restructur* OR reapprais*)):ti,ab,kw OR refram*:ti,ab,kw OR 'Self identity'/de OR 'Self identit*':ti,ab,kw OR (Chang* NEAR/4 (Cognition* OR Emotion*)):ti,ab,kw OR 'Emotion regulation'/de OR (Emotion* NEAR/4 Regulat*):ti,ab,kw OR (Shifting NEAR/4 Perspectiv*):ti,ab,kw OR (Valued NEAR/4 'self-identit*'):ti,ab,kw OR (Mental NEAR/4 Rehears*):ti,ab,kw OR (Past NEAR/4 Succes*):ti,ab,kw OR (Pain NEAR/4 Belief*):ti,ab,kw OR 'Patient compliance'/de OR adherence:ti,ab,kw OR complian*:ti,ab,kw OR 'Self concept'/de OR 'Self concept*':ti,ab,kw OR 'self efficacy':ti,ab,kw OR maintenance*:ti,ab,kw OR maintain*:ti,ab,kw OR relaps*:ti,ab,kw OR 'Recurrence risk'/de OR recurren*:ti,ab,kw OR exacerbation*:ti,ab,kw OR 'Secondary prevention'/de OR ((Secundary OR Secondary) NEAR/4 Prevention*):ti,ab,kw OR 'Illness behavior'/de OR 'Health behavior'/de OR 'Attitude'/de OR 'Attitude to health'/de OR 'attitude to change'/de OR 'attitude to disability'/de OR 'attitude to illness'/de OR 'Attitud*':ti,ab,kw OR 'Health belief'/de OR ((Illness OR Disease* OR Sickness* OR Health) NEAR/4 (Behavior* OR Behaviour* OR Belief*)):ti,ab,kw OR 'Risk reduction'/de OR 'Risk reduction*':ti,ab,kw)

I4: Selfmanagment support 5,613,289 treffers 8-12-2022

('Self control'/exp OR 'self-manage*':ti,ab,kw OR selfmanage*:ti,ab,kw OR 'self-control*':ti,ab,kw OR selfcontrol*:ti,ab,kw OR selfregulation*:ti,ab,kw OR 'self-regulation*':ti,ab,kw OR 'emotional regulation*':ti,ab,kw OR 'self care'/de OR 'self care':ti,ab,kw OR 'self caring':ti,ab,kw OR 'self help'/de OR 'self help':ti,ab,kw OR 'Empowerment'/de OR 'patient empowerment'/de OR empower*:ti,ab,kw OR 'Patient education'/de OR (Patient* NEAR/4 (Educat* OR Train*)):ti,ab,kw OR 'Health education'/de OR 'Consumer health information'/de OR 'Health literacy'/exp OR ((Health OR Medical) NEAR/4 (Education OR Literac* OR Illiterac* OR Information* OR Knowledg* OR Skill* OR Comprehen* OR Attitude*)):ti,ab,kw OR 'Interpersonal communication'/de OR 'teach-back':ti,ab,kw OR 'teach back':ti,ab,kw OR 'Knowledge'/de OR 'attitude to health'/de OR 'Education'/de OR diary:ti,ab,kw OR diaries:ti,ab,kw OR handout*:ti,ab,kw OR 'hand-out*':ti,ab,kw OR 'Audiovisual aid'/de OR (Teaching NEAR/4 Material*):ti,ab,kw OR 'Book'/de OR book*:ti,ab,kw OR 'medical illustration'/de OR 'medical illustration*':ti,ab,kw OR leaflet*:ti,ab,kw OR 'Videorecording'/de OR video*:ti,ab,kw OR 'audiovisual recording'/de OR 'audiovisual':ti,ab,kw OR 'audio-visual':ti,ab,kw OR film:ti,ab,kw OR films:ti,ab,kw OR tutorial*:ti,ab,kw OR webcast*:ti,ab,kw OR podcast*:ti,ab,kw OR youtube*:ti,ab,kw OR inform*:ti,ab,kw OR (Knowledge NEAR/4 (Disease* OR Ilness* OR Disorder*)):ti,ab,kw OR 'Mentoring'/de OR mentor*:ti,ab,kw OR 'pros and cons':ti,ab,kw OR (Decisional NEAR/4 Balanc*):ti,ab,kw OR (Comparative NEAR/4 Imag*):ti,ab,kw OR 'Directive counseling'/de OR (Directive NEAR/4 Counseling):ti,ab,kw OR 'motivational interviewing'/de OR (Motivational NEAR/4 Interview*):ti,ab,kw OR (Body NEAR/4 Awareness):ti,ab,kw OR 'active listening'/de OR (Active NEAR/4 Listening):ti,ab,kw OR (Relaxation NEAR/4 Techni*):ti,ab,kw OR (Pain NEAR/4 Provocation*):ti,ab,kw OR 'Psychological adjustment'/de OR 'job adaptation'/de OR ((Psychological OR occupational OR work OR job) NEAR/4 (Adaptation* OR Adjustment*)):ti,ab,kw OR 'adaptive behavio*':ti,ab,kw OR 'coping behavior'/de OR 'support-seeking'/de OR 'stress management'/de OR 'problem-focused coping'/de OR 'emotion-focused coping'/de OR coping:ti,ab,kw OR 'stress management':ti,ab,kw OR (support NEAR/4 seeking):ti,ab,kw OR regulation:ti,ab,kw OR 'Lifestyle'/exp OR (Life NEAR/4 Style*):ti,ab,kw OR 'lifestyle*':ti,ab,kw OR 'Cognitive behavioral therapy'/de OR (Cognitive NEAR/4 Behavio* NEAR/4 (Therap* OR Treat*)):ti,ab,kw OR CBT:ti,ab,kw OR 'Health Belief Model'/de OR (Health NEAR/4 Belief*):ti,ab,kw OR 'Transtheoretical model'/de OR ((Transtheoretical OR trans-theoretical OR transactional OR 'trans-actional' OR biopsychosocial OR 'bio-psychosocial') NEAR/4 Model*):ti,ab,kw OR TTM:ti,ab,kw OR 'biopsychosocial model'/de OR (Stages NEAR/4 Change*):ti,ab,kw OR prochaska*:ti,ab,kw OR (Pain NEAR/4 Belief*):ti,ab,kw OR (internal NEAR/4 external NEAR/4 control*):ti,ab,kw OR ((Sense OR Locus) NEAR/4 Control*):ti,ab,kw OR 'Health promotion'/de OR (Health NEAR/4 Promotion*):ti,ab,kw OR (Attitude NEAR/4 (lllness* OR Disease* OR Disorder* OR health)):ti,ab,kw OR (Common NEAR/4 Sense NEAR/4 Model*):ti,ab,kw OR Huber*:ti,ab,kw OR (Positive NEAR/4 Health):ti,ab,kw OR 'behavior theory'/de OR 'behavior theor*':ti,ab,kw OR 'behaviour theor*':ti,ab,kw OR 'Self regulation model'/de OR 'Lazarus theory of stress and coping'/de OR 'Stress theory'/de OR 'Social Cognitive Theory'/de OR 'Social Learning Theory'/de OR 'Theory of Planned Behavior'/de OR 'Theory of Reasoned Action'/de OR ((Stress OR 'Social Cognitive' OR 'Social Learning' OR 'Planned Behavio*' OR 'Reasoned Action') NEAR/4 Theory):ti,ab,kw)

O1: Movement behaviour (5,619,586 treffers 8-12-22)

('Movement (physiology)'/de OR "movement*":ti,ab,kw OR 'Coordination'/de OR coordination*:ti,ab,kw OR (Movement* NEAR/4 (Fluenc* OR symmetr* OR Asymmetr*)):ti,ab,kw OR compensat*:ti,ab,kw OR "co-contraction":ti,ab,kw OR "cocontraction":ti,ab,kw OR 'Muscle tone'/de OR ((Muscle OR Muscular) NEAR/4 (Tonus OR Tone OR Tones OR Tension OR Tightness* OR Contraction* OR Relaxation* OR Function*)):ti,ab,kw OR agonist*:ti,ab,kw OR antagonist*:ti,ab,kw OR 'Muscle contraction'/de OR Inotropism*:ti,ab,kw OR 'Muscle relaxation'/de OR 'job performance'/de OR ((work OR job OR occupational OR Task OR Tasks) NEAR/4 Performanc*):ti,ab,kw OR 'Task performance'/de OR (Task NEAR/4 Analy*):ti,ab,kw OR (workload NEAR/4 measurement*):ti,ab,kw OR (Critical NEAR/4 Incident NEAR/4 Techn*):ti,ab,kw OR 'Body equilibrium'/exp OR (Postural NEAR/4 (Control* OR Equilibri*)):ti,ab,kw OR (Core NEAR/4 Stabilit*):ti,ab,kw OR balanc*:ti,ab,kw OR unbalanc*:ti,ab,kw OR align*:ti,ab,kw OR 'Ergonomics'/exp OR ergonomic*:ti,ab,kw OR 'Environment'/de OR environment*:ti,ab,kw OR ((Physical OR Sedentary) NEAR/4 Time):ti,ab,kw OR 'Time factor'/de OR (Minutes NEAR/4 (Week OR Day)):ti,ab,kw OR bout*:ti,ab,kw OR 'Sedentary lifestyle'/de OR (Sedentary NEAR/4 (Behavio* OR Lifestyle* OR "life style*")):ti,ab,kw OR (Physical NEAR/4 Inactivit*):ti,ab,kw OR (lack* NEAR/4 physical NEAR/4 activit*):ti,ab,kw OR 'Heart rate'/de OR ((Heart OR Cardiac OR Pulse*) NEAR/4 (Rate* OR Beat*)):ti,ab,kw OR HRR:ti,ab,kw OR 'Metabolic equivalent'/de OR MET:ti,ab,kw OR (Metabolic NEAR/4 Equivalent):ti,ab,kw OR ((Light OR High OR Moderate OR Vigorous) NEAR/4 Intensit*):ti,ab,kw OR ((Physical OR functional) NEAR/4 Activit*):ti,ab,kw OR pace:ti,ab,kw OR paces:ti,ab,kw OR 'Walking speed'/de OR speed*:ti,ab,kw OR 'velocity'/de OR velocity:ti,ab,kw OR 'Acceleration'/de OR accelerat*:ti,ab,kw OR 'kinematics'/de OR kinematics:ti,ab,kw OR 'Range of motion'/de OR (Range NEAR/4 Motion*):ti,ab,kw OR ROM:ti,ab,kw OR (flexibilit* NEAR/4 joint*):ti,ab,kw OR 'Physical performance'/de OR ((functional OR physical) NEAR/4 performanc*):ti,ab,kw)

O2: Activities of daily living ( 7,852,104 treffers op 8-12-2022)

('Disability'/de OR 'ADL disability'/de OR 'Physical disability'/de OR disabilit*:ti,ab,kw OR 'International Classification of Functioning, Disability and Health'/de OR ICF:ti,ab,kw OR 'Walking difficulty'/de OR (mobility NEAR/4 limit*):ti,ab,kw OR 'Daily life activity'/de OR (Daily NEAR/4 (Activit* OR Life OR Living OR Lives)):ti,ab,kw OR ADL:ti,ab,kw OR everyday:ti,ab,kw OR 'every day':ti,ab,kw OR 'Functional status'/de OR (Functional NEAR/4 (Status OR Dependen* OR Indepen*)):ti,ab,kw OR 'Work'/de OR 'Return to work'/de OR 'Work-life balance'/de OR work*:ti,ab,kw OR 'work environment'/de OR job:ti,ab,kw OR jobs:ti,ab,kw OR Commut*:ti,ab,kw OR sport*:ti,ab,kw OR leisure*:ti,ab,kw OR hobb*:ti,ab,kw OR 'Home environment'/de OR home*:ti,ab,kw OR (Living NEAR/4 Alone):ti,ab,kw OR 'School'/de OR school*:ti,ab,kw OR 'Student'/de OR student*:ti,ab,kw OR 'Child care'/de OR ((Child OR Day OR Informal)NEAR/4 Care):ti,ab,kw OR puericulture:ti,ab,kw OR caregiv*:ti,ab,kw OR (Care NEAR/4 Giv*):ti,ab,kw OR 'Volunteer'/de OR volunteer*:ti,ab,kw OR 'Social participation'/de OR ((Social* OR sports) NEAR/4 (Participati* OR involv* OR engag*)):ti,ab,kw OR (('Body position'/de OR postur*:ti,ab,kw OR 'Standing'/exp OR stand:ti,ab,kw OR standing:ti,ab,kw OR 'Sitting'/de OR sit:ti,ab,kw OR sitting:ti,ab,kw OR 'seated':ti,ab,kw OR 'recumbency'/de OR recumben*:ti,ab,kw OR lying:ti,ab,kw OR 'Prone position'/de OR (Prone NEAR/4 Position*):ti,ab,kw OR 'Supine position'/de OR (Supine NEAR/4 Position*):ti,ab,kw OR ambulat*:ti,ab,kw OR gait*:ti,ab,kw OR 'Walking'/exp OR walk*:ti,ab,kw OR stoop*:ti,ab,kw OR step*:ti,ab,kw OR bend*:ti,ab,kw OR squat*:ti,ab,kw OR push*:ti,ab,kw OR pull*:ti,ab,kw OR ((Golf OR Golfer) NEAR/4 Techn*):ti,ab,kw OR 'Weight lifting'/de OR wlt:ti,ab,kw OR 'lift*':ti,ab,kw OR 'carry*':ti,ab,kw OR jump*:ti,ab,kw OR 'Stair climbing'/de OR (Stair NEAR/4 (Climb* OR Navigat*)):ti,ab,kw OR rising:ti,ab,kw OR rise:ti,ab,kw OR (sit NEAR/4 stand):ti,ab,kw OR (Chair NEAR/4 Bed):ti,ab,kw OR reach*:ti,ab,kw OR throw*:ti,ab,kw OR 'Locomotion'/de OR locomot*:ti,ab,kw OR 'Running'/de OR run:ti,ab,kw OR running:ti,ab,kw OR jog:ti,ab,kw OR jogging:ti,ab,kw OR 'Head movement'/de OR (Head NEAR/4 (Movement* OR Moving*)):ti,ab,kw)))

FILTERS

RCT-filter

AND ('controlled clinical trial'/exp OR 'randomization'/de OR 'random sample'/de OR 'single blind procedure'/de OR 'double blind procedure'/de OR 'triple blind procedure'/de OR 'quasi experimental study'/de OR 'pretest posttest control group design'/de OR (rct OR random* OR trial* OR 'single-blind*' OR 'single-masked' OR 'double-blind*' OR 'double masked' OR 'triple-blind*' OR 'triple-masked' OR 'quasi-experimental' OR 'pre-test' OR 'pretest' OR 'post-test' OR 'posttest'):ti,ab,kw)

NOT protocol:ti
