# Supplementary material for: Evaluating the Effectiveness and Technique Composition of Combined Motor Learning and Behavior Change Strategies for Movement Behavior in Chronic Musculoskeletal Pain: A Systematic Review
Source: Arch Rehabil Res Clin Transl. 2026 Mar 24;8(3):100618. doi: 10.1016/j.arrct.2026.100618 (PMC13591100; doi:10.1016/j.arrct.2026.100618)
Supplement: Supplementary file 3 [file mmc3.docx]

Supplement 3. Search in CINAHL

Patiënt

((MH "Chronic Pain" OR TI (((chronic OR persist* OR recurren* OR "long-standing" OR ongoing OR "on-going" OR continu* OR prolong*) N3 (Pain* OR Ache* OR complain*)))) OR (MH "Musculoskeletal Diseases" OR TI msd OR MH "Musculoskeletal Pain" OR TI ((musculoskeletal OR muskuloskeletal OR "musculo-skeletal" OR "muskulo-skeletal" OR muscle*) N3 (Disease* OR Pain* OR ache* OR condition* OR Disorder* OR problem* OR soreness* OR tenderness*)) OR (chronic N3 (Disease* OR condition* OR Disorder* OR problem* OR soreness* OR tenderness*)) OR MH "Muscle Pain" OR TI myalgia OR MH "Arthralgia+" OR TI arthralgia OR MH "Pelvic Pain" OR MH "Back Pain" OR MH "Low Back Pain" OR TI "backpain*" OR TI "backache*" OR TI lumbago* OR TI LBP OR MH "Sciatica" OR TI Sciatica OR MH "Neck Pain" OR TI "neckpain*" OR TI "neckache*" OR TI cervicalgia* OR TI cervicodynia* OR MH "Whiplash Injuries" OR TI whiplash* OR TI "acceleration cranial trauma*" OR TI "deceleration cranial trauma" OR TI ADT OR MH "shoulder impingement syndrome" OR TI ((Shoulder OR Rotator OR Cuff OR Coracohumeral OR "Coraco-humeral" OR Coracoid OR Subacromial OR "Sub-acromial" OR Outlet OR Internal OR Glenoid*) N3 Impingement*) OR MH "Bursitis" OR TI bursitis OR TI bursitides OR TI "frozen shoulder*" OR TI capsulitis OR TI capsulitides OR MH "Shoulder Pain" OR TI "shoulderpain*" OR TI "shoulderache*" OR MH "Tennis Elbow" OR MH "Golf Elbow" OR TI epicondylitis OR TI epicondylitides OR TI "elbow tendinopath*" OR TI ((Tennis OR Golf*) N3 Elbow*) OR MH "Nerve Compression Syndromes" OR TI ((Nerve OR Neuropath*) N3 (Compression OR Entrapment*)) OR TI ((plexus OR nerve) N3 (impingement* OR entrapment*)) OR MH "Carpal Tunnel Syndrome" OR TI "carpal tunnel" OR MH "Cubital Tunnel Syndrome" OR TI "cubital tunnel" OR TI (Radial N3 Neuropath*) OR TI ((Supinator OR "Radial tunnel" OR "Radial nerve") N3 (Syndrome* OR Disease*)) OR TI (Radial N3 Mononeuropath*) OR TI "wrist-drop" OR MH "Thoracic Outlet Syndrome" OR TI (("thoracic outlet" OR "thoracic aperture" OR "thorax outlet" OR "thorax aperture" OR Costoclavicular OR "scalenus anticus" OR "cervical rib" OR Naffziger*) N3 syndrome*) OR TI tos OR TI "complaints arm neck shoulder*" OR TI cans OR MH "Cauda Equina Syndrome" OR TI (("Cauda equina" OR Piriformis OR "Tarsal tunnel") N3 Syndrome*) OR MH "Piriformis Syndrome" OR MH "Tarsal Tunnel Syndrome" OR MH "Intervertebral Disk Displacement" OR TI ((displacement* OR protru* OR hernia* OR slipped OR prolapse*) N3 (disk* OR disc*)) OR MH "Spinal Stenosis" OR TI "spinal stenosis" OR TI "spinal stenoses" OR TI "vertebral canal stenos*" OR MH "Fatigue Syndrome, Chronic" OR TI "chronic fatigue" OR MH "fibromyalgia" OR TI fibromyalgi* OR TI (Muscular N3 Rheuma*) ORTI fibrositis OR TI fibrositides OR TI "myofascial pain" OR MH "Osteoporosis" OR TI osteoporosis OR TI osteoporoses OR TI "osteo-porosis" OR TI "osteo-poroses" OR TI "bone loss*" OR MH "Spondylarthritis" OR MH "Spondylarthropathies" OR MH "Axial Spondyloarthritis" OR TI spondylarthritis OR TI spondyloarthritis OR TI spondylarthritides OR TI spondyloarthritides OR TI spondyloarthropath* OR TI "spondylo-arthropath*" OR TI spondylarthropath* OR TI "spondyl-arthropath*" OR MH "Spondylitis, Ankylosing" OR MH "Ankylosis" OR TI Bechterew* OR TI (Ankylo* N3 Spondyl*) OR MH "Osteoarthritis" OR MH "Osteoarthritis, Shoulder" OR MH "Osteoarthritis, Wrist" OR MH "Osteoarthritis, Spine+" OR TI "osteoarthritis" OR TI "osteoarthritides" OR TI "osteoarthrosis" OR TI "osteoarthroses" OR TI "arthrosis" OR TI "arthroses" OR MH "Osteoarthritis, Hand" OR MH "Osteoarthritis, Hip" OR MH "Osteoarthritis, Knee" OR MH "Spondylosis+" OR TI ((arthritic OR osteoarthritic OR "osteo-arthritic") AND (Spine* OR Knee* OR Hip OR Hips)) OR MH "Arthritis" OR arthritis OR TI arthritides OR TI monarthritis OR TI Polyarthritis OR TI Polyarthritides OR MH "Arthritis, Rheumatoid" OR TI s**pondylitis** OR MH "Spinal Curvatures+" OR TI (Spinal N3 Curv*) OR TI kyphosis OR TI kyphoses OR TI hyperkyphosis OR TI hyperkyphoses OR MH "Scheuermann"s Disease" OR TI scheuermann* OR TI lordosis OR TI lordoses OR TI hyperlordosis OR TI hyperlordoses OR TI scoliosis OR TI scolioses OR TI spondylolisthesis OR TI spondylolysis OR OR TI spondylosis OR TI (Back OR "Pelvic girdle*" OR Neck OR Cervical OR Shoulder* OR Elbow* OR Wrist* OR Hand OR Hands OR Hip OR Hips OR Knee OR Knees OR Ankle* OR Foot OR Feet OR discogenic OR Joint* OR Metatarsophalangeal* OR Spine* OR Spinal* OR Vertebra*) OR TI (Articulationes N3 pedis) OR TI "cervical atlas" OR TI (Intervertebral N3 dis*) OR TI "annulus fibrosus" OR TI "nucleus pulposus" OR MH "Elbow" OR MH "Wrist" OR MH "Hand Joints" OR MH "Hand" OR MH "Foot" OR MH "Hip" OR MH "Knee" OR MH "Ankle" OR MH "Spine" OR MH "Metatarsophalangeal Joint" OR MH "Cervical Vertebrae+" OR MH "Intervertebral Disk+" OR MH "Lumbar Vertebrae" OR MH "Upper Extremity" OR MH "Lower Extremity" OR TI ((Lower OR Upper) N3 (Extremit* OR Limb*)))) OR ((MH "Chronic Pain" OR AB (((chronic OR persist* OR recurren* OR "long-standing" OR ongoing OR "on-going" OR continu* OR prolong*) N3 (Pain* OR Ache* OR complain*)))) OR (MH "Musculoskeletal Diseases" OR AB msd OR MH "Musculoskeletal Pain" OR AB ((musculoskeletal OR muskuloskeletal OR "musculo-skeletal" OR "muskulo-skeletal" OR muscle*) N3 (Disease* OR Pain* OR ache* OR condition* OR Disorder* OR problem* OR soreness* OR tenderness*)) OR (chronic N3 (Disease* OR condition* OR Disorder* OR problem* OR soreness* OR tenderness*)) OR MH "Muscle Pain" OR AB myalgia OR MH "Arthralgia+" OR AB arthralgia OR MH "Pelvic Pain" OR MH "Back Pain" OR MH "Low Back Pain" OR AB "backpain*" OR AB "backache*" OR AB lumbago* OR AB LBP OR MH "Sciatica" OR AB Sciatica OR MH "Neck Pain" OR AB "neckpain*" OR AB "neckache*" OR AB cervicalgia* OR AB cervicodynia* OR MH "Whiplash Injuries" OR AB whiplash* OR AB "acceleration cranial trauma*" OR AB "deceleration cranial trauma" OR AB ADT OR MH "shoulder impingement syndrome" OR AB ((Shoulder OR Rotator OR Cuff OR Coracohumeral OR "Coraco-humeral" OR Coracoid OR Subacromial OR "Sub-acromial" OR Outlet OR Internal OR Glenoid*) N3 Impingement*) OR MH "Bursitis" OR AB bursitis OR AB bursitides OR AB "frozen shoulder*" OR AB capsulitis OR AB capsulitides OR MH "Shoulder Pain" OR AB "shoulderpain*" OR AB "shoulderache*" OR MH "Tennis Elbow" OR MH "Golf Elbow" OR AB epicondylitis OR AB epicondylitides OR AB "elbow tendinopath*" OR AB ((Tennis OR Golf*) N3 Elbow*) OR MH "Nerve Compression Syndromes" OR AB ((Nerve OR Neuropath*) N3 (Compression OR Entrapment*)) OR AB ((plexus OR nerve) N3 (impingement* OR entrapment*)) OR MH "Carpal Tunnel Syndrome" OR AB "carpal tunnel" OR MH "Cubital Tunnel Syndrome" OR AB "cubital tunnel" OR AB (Radial N3 Neuropath*) OR AB ((Supinator OR "Radial tunnel" OR "Radial nerve") N3 (Syndrome* OR Disease*)) OR AB (Radial N3 Mononeuropath*) OR AB "wrist-drop" OR MH "Thoracic Outlet Syndrome" OR AB (("thoracic outlet" OR "thoracic aperture" OR "thorax outlet" OR "thorax aperture" OR Costoclavicular OR "scalenus anticus" OR "cervical rib" OR Naffziger*) N3 syndrome*) OR AB tos OR AB "complaints arm neck shoulder*" OR AB cans OR MH "Cauda Equina Syndrome" OR AB (("Cauda equina" OR Piriformis OR "Tarsal tunnel") N3 Syndrome*) OR MH "Piriformis Syndrome" OR MH "Tarsal Tunnel Syndrome" OR MH "Intervertebral Disk Displacement" OR AB ((displacement* OR protru* OR hernia* OR slipped OR prolapse*) N3 (disk* OR disc*)) OR MH "Spinal Stenosis" OR AB "spinal stenosis" OR AB "spinal stenoses" OR AB "vertebral canal stenos*" OR MH "Fatigue Syndrome, Chronic" OR AB "chronic fatigue" OR MH "fibromyalgia" OR AB fibromyalgi* OR AB (Muscular N3 Rheuma*) ORAB fibrositis OR AB fibrositides OR AB "myofascial pain" OR MH "Osteoporosis" OR AB osteoporosis OR AB osteoporoses OR AB "osteo-porosis" OR AB "osteo-poroses" OR AB "bone loss*" OR MH "Spondylarthritis" OR MH "Spondylarthropathies" OR MH "Axial Spondyloarthritis" OR AB spondylarthritis OR AB spondyloarthritis OR AB spondylarthritides OR AB spondyloarthritides OR AB spondyloarthropath* OR AB "spondylo-arthropath*" OR AB spondylarthropath* OR AB "spondyl-arthropath*" OR MH "Spondylitis, Ankylosing" OR MH "Ankylosis" OR AB Bechterew* OR AB (Ankylo* N3 Spondyl*) OR MH "Osteoarthritis" OR MH "Osteoarthritis, Shoulder" OR MH "Osteoarthritis, Wrist" OR MH "Osteoarthritis, Spine+" OR AB "osteoarthritis" OR AB "osteoarthritides" OR AB "osteoarthrosis" OR AB "osteoarthroses" OR AB "arthrosis" OR AB "arthroses" OR MH "Osteoarthritis, Hand" OR MH "Osteoarthritis, Hip" OR MH "Osteoarthritis, Knee" OR MH "Spondylosis+" OR AB ((arthritic OR osteoarthritic OR "osteo-arthritic") AND (Spine* OR Knee* OR Hip OR Hips)) OR MH "Arthritis" OR arthritis OR AB arthritides OR AB monarthritis OR AB Polyarthritis OR AB Polyarthritides OR MH "Arthritis, Rheumatoid" OR AB s**pondylitis** OR MH "Spinal Curvatures+" OR AB (Spinal N3 Curv*) OR AB kyphosis OR AB kyphoses OR AB hyperkyphosis OR AB hyperkyphoses OR MH "Scheuermann"s Disease" OR AB scheuermann* OR AB lordosis OR AB lordoses OR AB hyperlordosis OR AB hyperlordoses OR AB scoliosis OR AB scolioses OR AB spondylolisthesis OR AB spondylolysis OR OR AB spondylosis OR AB (Back OR "Pelvic girdle*" OR Neck OR Cervical OR Shoulder* OR Elbow* OR Wrist* OR Hand OR Hands OR Hip OR Hips OR Knee OR Knees OR Ankle* OR Foot OR Feet OR discogenic OR Joint* OR Metatarsophalangeal* OR Spine* OR Spinal* OR Vertebra*) OR AB (Articulationes N3 pedis) OR AB "cervical atlas" OR AB (Intervertebral N3 dis*) OR AB "annulus fibrosus" OR AB "nucleus pulposus" OR MH "Elbow" OR MH "Wrist" OR MH "Hand Joints" OR MH "Hand" OR MH "Foot" OR MH "Hip" OR MH "Knee" OR MH "Ankle" OR MH "Spine" OR MH "Metatarsophalangeal Joint" OR MH "Cervical Vertebrae+" OR MH "Intervertebral Disk+" OR MH "Lumbar Vertebrae" OR MH "Upper Extremity" OR MH "Lower Extremity" OR AB ((Lower OR Upper) N3 (Extremit* OR Limb*))))

I1: Exercise therapy

(MH "Resistance Training" OR TI ((Resistance OR Strength*) N3 training*) OR TI "weight lifting" OR MH "Stretching" OR TI (Muscle N3 Stretch*) OR MH "Balance Training, Physical" OR TI (balance N3 train*) OR MH "Exercise" OR TI exercis* OR MH "Therapeutic Exercise" OR MH "Physical Therapy" OR TI physiotherap* OR TI (Physical N3 (Therap* OR Activit*)) OR MH "Functional Training" OR MH "Gait Training" OR TI ((funtional OR gait) N3 train*) OR MH "Home Physical Therapy" OR TI "training intervention*" OR MH "Breathing Exercises" OR TI ((Breathing* OR Respirator* OR Pulmonary* OR Lung*) N3 (Train*)) OR MH "Rehabilitation" OR MH "Home Rehabilitation+" OR MH "Rehabilitation, Psychosocial+" OR TI rehabilitat* OR TI habilitat* OR MH "Recovery" OR TI Convalescence OR TI (recover* N3 function*) OR TI refunction* OR TI "re-function*" OR MH "Rehabilitation Centers" OR MH "Occupational Therapy" OR TI (occupational N3 therap*) OR MH "Telerehabilitation" OR TI "telerehabilitat*" OR TI "tele-rehabilitat*" OR TI (("Daily activities" OR "Daily activity") NEAR Monitor*) OR TI (Individualized N3 Feedback*) OR TI cue OR TI cues OR TI cueing OR TI remind* OR MH "Reminder Systems" OR TI texting OR TI homework OR MH "Telemedicine" OR MH "Remote Consultation" OR MH "Telenursing" OR MH "User-Computer Interface" OR MH "Multimedia" OR MH "Cellular Phone" OR MH "Medical Informatics" OR MH "Nursing Informatics" OR MH "Computers, Hand-Held+" OR MH "Mobile Applications" OR MH "Internet-Based Intervention" OR MH "Patient Portals" OR TI "ediagnos*" OR TI "e diagnos*" OR TI "mobile health*" OR TI "mhealth*" OR TI "m health*" OR MH "Telehealth" OR TI "telehealth*" OR TI "tele health" OR TI "teleconsult*" OR TI "Telenursing" OR TI "tele nursing" OR TI "telediagnos*" OR TI "tele diagnos*" OR TI "telemedic*" OR TI "tele medic*" OR TI "telemonitor*" OR TI "remote selfmonitor*" OR TI "ehealth*" OR TI "e health*" OR TI "telecare" OR TI "tele care" OR TI (Digital N3 Health) OR TI "app" OR TI "apps" OR TI "smartphone*" OR TI ((Phone* OR Telephone* OR Mobile* OR Health OR portable) N3 (Application* OR Technolog*)) OR TI "internet*" OR TI "web-based" OR TI "web-oriented" OR TI "world wide web*" OR TI "webportal*" OR TI "web portal*" OR TI "patient portal*" OR TI "ipad" OR TI "ipads" OR TI "sms" OR TI "mms" OR TI "text messag*" OR TI "ussd" OR TI "pda" OR TI "laptop*" OR TI "palmtop*" OR TI "palm top*" OR TI "personal digital assistant*" OR TI "telecounsel*" OR TI ((Distan* OR Online OR "On-line" OR Remote OR mobile OR tele OR "e-") N3 (Counsel* OR Consult* OR Intervention* OR Educat* OR Learning* OR monitor*)) OR TI ecounsel* OR TI econsult* OR TI eintervention* OR TI eeducat* OR TI elearning* OR TI "patient monitoring" OR TI "interactive voice response*" OR TI "Multimedia" OR TI "Mhapps" OR TI "iphone*" OR TI "android" OR TI "game*" OR TI "gaming" OR TI "gamification" OR TI "whatsapp*" OR TI "e coach*" OR TI "wearable*" OR TI "exergam*" OR TI "serious gam*" OR TI (Personal N3 Health N3 Record*) OR TI "health kiosk*" OR TI "digital care" OR MH "Combined Modality Therapy") OR TI ((Multimodal OR "Multi-modal" OR "Combined modality") N3 (Therap* OR Treatment* OR Intervention*)) ) OR (MH "Resistance Training" OR AB ((Resistance OR Strength*) N3 training*) OR AB "weight lifting" OR MH "Stretching" OR AB (Muscle N3 Stretch*) OR MH "Balance Training, Physical" OR AB (balance N3 train*) OR MH "Exercise" OR AB exercis* OR MH "Therapeutic Exercise" OR MH "Physical Therapy" OR AB physiotherap* OR AB (Physical N3 (Therap* OR Activit*)) OR MH "Functional Training" OR MH "Gait Training" OR AB ((funtional OR gait) N3 train*) OR MH "Home Physical Therapy" OR AB "training intervention*" OR MH "Breathing Exercises" OR AB ((Breathing* OR Respirator* OR Pulmonary* OR Lung*) N3 (Train*)) OR MH "Rehabilitation" OR MH "Home Rehabilitation+" OR MH "Rehabilitation, Psychosocial+" OR AB rehabilitat* OR AB habilitat* OR MH "Recovery" OR AB Convalescence OR AB (recover* N3 function*) OR AB refunction* OR AB "re-function*" OR MH "Rehabilitation Centers" OR MH "Occupational Therapy" OR AB (occupational N3 therap*) OR MH "Telerehabilitation" OR AB "telerehabilitat*" OR AB "tele-rehabilitat*" OR AB (("Daily activities" OR "Daily activity") NEAR Monitor*) OR AB (Individualized N3 Feedback*) OR AB cue OR AB cues OR AB cueing OR AB remind* OR MH "Reminder Systems" OR AB texting OR AB homework OR MH "Telemedicine" OR MH "Remote Consultation" OR MH "Telenursing" OR MH "User-Computer Interface" OR MH "Multimedia" OR MH "Cellular Phone" OR MH "Medical Informatics" OR MH "Nursing Informatics" OR MH "Computers, Hand-Held+" OR MH "Mobile Applications" OR MH "Internet-Based Intervention" OR MH "Patient Portals" OR AB "ediagnos*" OR AB "e diagnos*" OR AB "mobile health*" OR AB "mhealth*" OR AB "m health*" OR MH "Telehealth" OR AB "telehealth*" OR AB "tele health" OR AB "teleconsult*" OR AB "Telenursing" OR AB "tele nursing" OR AB "telediagnos*" OR AB "tele diagnos*" OR AB "telemedic*" OR AB "tele medic*" OR AB "telemonitor*" OR AB "remote selfmonitor*" OR AB "ehealth*" OR AB "e health*" OR AB "telecare" OR AB "tele care" OR AB (Digital N3 Health) OR AB "app" OR AB "apps" OR AB "smartphone*" OR AB ((Phone* OR Telephone* OR Mobile* OR Health OR portable) N3 (Application* OR Technolog*)) OR AB "internet*" OR AB "web-based" OR AB "web-oriented" OR AB "world wide web*" OR AB "webportal*" OR AB "web portal*" OR AB "patient portal*" OR AB "ipad" OR AB "ipads" OR AB "sms" OR AB "mms" OR AB "text messag*" OR AB "ussd" OR AB "pda" OR AB "laptop*" OR AB "palmtop*" OR AB "palm top*" OR AB "personal digital assistant*" OR AB "telecounsel*" OR AB ((Distan* OR Online OR "On-line" OR Remote OR mobile OR tele OR "e-") N3 (Counsel* OR Consult* OR Intervention* OR Educat* OR Learning* OR monitor*)) OR AB ecounsel* OR AB econsult* OR AB eintervention* OR AB eeducat* OR AB elearning* OR AB "patient monitoring" OR AB "interactive voice response*" OR AB "Multimedia" OR AB "Mhapps" OR AB "iphone*" OR AB "android" OR AB "game*" OR AB "gaming" OR AB "gamification" OR AB "whatsapp*" OR AB "e coach*" OR AB "wearable*" OR AB "exergam*" OR AB "serious gam*" OR AB (Personal N3 Health N3 Record*) OR AB "health kiosk*" OR AB "digital care" OR MH "Combined Modality Therapy") OR AB ((Multimodal OR "Multi-modal" OR "Combined modality") N3 (Therap* OR Treatment* OR Intervention*)) )

I2: Motor Learning

(MH "Psychomotor Performance+" OR TI ((psychomotor OR motor) N3 (control* OR coordinat* OR perform* OR skill* OR activit*)) OR MH "Motor Activity" OR TI "motor activit*" OR TI imitat* OR TI ((Motor OR Movement*) N3 Imagery) OR TI (Mental N3 Practic*) OR MH "Reflection" OR TI reflect* OR MH "Learning" OR MH "Imitative Behavior" OR MH "Experiential Learning" OR MH "Skill Retention" OR MH "Transfer (Psychology)" OR TI "learn*" OR TI experien* OR TI imprint* OR TI (skill* N3 retention) OR TI (psychology N3 transfer*) OR TI Phenomenograph* OR TI (Memory N3 Training) OR TI (Psychological N3 Practic*) OR MH "Reinforcement (Psychology)" OR TI ((Psycholog* OR Positive OR Verbal* OR Social) N3 Reinforcement*) OR TI metaphor* OR TI metafor* OR TI (Instruction N3 (Perform* OR Task*)) OR TI (Attentional N3 Focus) OR MH "Attention" OR TI "attention" OR TI ((Internal* OR Intrinsic* OR External*) N3 Focus) OR TI (Implicit OR Explicit N3 Instruction*) OR TI (Rhythmic N3 Auditor*) OR TI RAS OR TI music OR TI counting OR TI (Manual N3 (Help* OR Correction*)) OR MH "Teaching" OR TI teach* OR TI instruct* OR TI ((Verbal OR Audio OR Auditory OR Tactile OR Visual* OR Mirror* OR Sensory OR Proprioceptive OR Sensorimotor OR Augmented OR Intrinsic* OR Extrinsic* OR Internal* OR External* OR Perform* OR Frequenc* OR positive OR psychological) N3 (Feedback* OR "Feed back*")) OR MH "Sensory Motor Integration" OR TI ((Sensorimotor OR Sensori-motor) N3 (Function* OR integration*)) OR MH "Mirror Therapy" OR TI (mirror* N3 therap*) OR TI photo OR TI smartphone* OR TI video* OR TI (Coach* N3 Eye*) OR MH "Biofeedback" OR TI biofeedback* OR TI "bio-feedback*" OR TI "biofeed back*" OR TI "bio-feed back*" OR MH "Feedback" OR MH "Proprioception+" OR TI proprioception OR TI ((Vestibular OR Equilibrium OR Labyrinthine OR Position OR Posture) N3 Sense*) OR TI kinesth* OR TI ((postur* OR musculoskeletal OR muskuloskeletal OR "musculo-skeletal" OR "muskulo-skeletal") N3 (equilibri* OR balance*)) OR TI (Core N3 (Stabilit* OR Balanc*)) OR TI (Knowledge N3 (Perform* OR Result*)) OR TI KP OR TI KR OR TI "feedforward*" OR TI (Feed N3 Forward*) OR MH "Cues" OR TI cue OR TI cues OR TI cueing OR TI ((Unstable OR Open OR Protective OR Static OR Predictable OR Simple OR Complex OR Variable OR Busy) N3 Environment*) OR TI "whole task*" OR TI "part task*" OR TI "subdivide task*" OR TI ((Interleaved OR Blocked OR Random OR Distributed OR Massed OR Organisation* OR Organization*) N3 Practice*)) OR (MH "Psychomotor Performance+" OR AB ((psychomotor OR motor) N3 (control* OR coordinat* OR perform* OR skill* OR activit*)) OR MH "Motor Activity" OR AB "motor activit*" OR AB imitat* OR AB ((Motor OR Movement*) N3 Imagery) OR AB (Mental N3 Practic*) OR MH "Reflection" OR AB reflect* OR MH "Learning" OR MH "Imitative Behavior" OR MH "Experiential Learning" OR MH "Skill Retention" OR MH "Transfer (Psychology)" OR AB "learn*" OR AB experien* OR AB imprint* OR AB (skill* N3 retention) OR AB (psychology N3 transfer*) OR AB Phenomenograph* OR AB (Memory N3 Training) OR AB (Psychological N3 Practic*) OR MH "Reinforcement (Psychology)" OR AB ((Psycholog* OR Positive OR Verbal* OR Social) N3 Reinforcement*) OR AB metaphor* OR AB metafor* OR AB (Instruction N3 (Perform* OR Task*)) OR AB (Attentional N3 Focus) OR MH "Attention" OR AB "attention" OR AB ((Internal* OR Intrinsic* OR External*) N3 Focus) OR AB (Implicit OR Explicit N3 Instruction*) OR AB (Rhythmic N3 Auditor*) OR AB RAS OR AB music OR AB counting OR AB (Manual N3 (Help* OR Correction*)) OR MH "Teaching" OR AB teach* OR AB instruct* OR AB ((Verbal OR Audio OR Auditory OR Tactile OR Visual* OR Mirror* OR Sensory OR Proprioceptive OR Sensorimotor OR Augmented OR Intrinsic* OR Extrinsic* OR Internal* OR External* OR Perform* OR Frequenc* OR positive OR psychological) N3 (Feedback* OR "Feed back*")) OR MH "Sensory Motor Integration" OR AB ((Sensorimotor OR Sensori-motor) N3 (Function* OR integration*)) OR MH "Mirror Therapy" OR AB (mirror* N3 therap*) OR AB photo OR AB smartphone* OR AB video* OR AB (Coach* N3 Eye*) OR MH "Biofeedback" OR AB biofeedback* OR AB "bio-feedback*" OR AB "biofeed back*" OR AB "bio-feed back*" OR MH "Feedback" OR MH "Proprioception+" OR AB proprioception OR AB ((Vestibular OR Equilibrium OR Labyrinthine OR Position OR Posture) N3 Sense*) OR AB kinesth* OR AB ((postur* OR musculoskeletal OR muskuloskeletal OR "musculo-skeletal" OR "muskulo-skeletal") N3 (equilibri* OR balance*)) OR AB (Core N3 (Stabilit* OR Balanc*)) OR AB (Knowledge N3 (Perform* OR Result*)) OR AB KP OR AB KR OR AB "feedforward*" OR AB (Feed N3 Forward*) OR MH "Cues" OR AB cue OR AB cues OR AB cueing OR AB ((Unstable OR Open OR Protective OR Static OR Predictable OR Simple OR Complex OR Variable OR Busy) N3 Environment*) OR AB "whole task*" OR AB "part task*" OR AB "subdivide task*" OR AB ((Interleaved OR Blocked OR Random OR Distributed OR Massed OR Organisation* OR Organization*) N3 Practice*))

I3: Behavioural change techniques

( MH "Behavioral Changes" OR TI ((Behavior* OR Behaviour*) N3 (Change*)) OR MH "Goals and Objectives" OR MH "Behavioral Objectives" OR MH "Goal Attainment" OR MH "Goal-Setting" OR TI Goal* OR MH "Motivation" OR TI motivation OR TI ((Action OR transition) N3 Plan*) OR MH "Commitment" OR TI "Commit*" OR MH "Problem Solving" OR TI (Problem* N3 solv*) OR MH "Decision Making, Shared" OR MH "Decision Making, Patient" OR TI (Shared N3 Decision*) OR TI (Patient N3 (Participati* OR Involv* OR Engag* OR Activiti*)) OR TI (Implementation N3 Intention*) OR TI (Conservat* N3 ("Mental Resourc*" OR "Mental effort")) OR MH "Mental Fatigue" OR TI (Mental N3 Fatigue) OR TI (Monitor* N3 (Behavio* OR Self)) OR TI "selfmonitoring" OR MH "Support, Social+" OR MH "Social Comparison" OR MH "Social Attitudes" OR TI ((Social OR psychological* OR psychosocial OR "psycho-social" OR emotional* OR community) N3 (Support* OR Care OR Approv* OR Comparison*)) OR TI "support system*" OR TI antecedent* OR TI "re-attribution*" OR TI "reattribution*" OR TI ((Health OR Social OR Emotional) N3 Consequence*) OR TI (Anticipated N3 Regret*) OR (Future N3 Punishment*) OR TI ((Behavior OR Behaviour) N3 Substitution*) OR MH "Habits" OR TI (Habit N3 (Formation* OR Reversal)) OR TI generalisation* OR generalization* OR TI (Graded N3 (Task* OR Activit* OR exercise* OR therap*)) OR MH "Reward" OR TI reward* OR TI "self-incentive*" OR TI ((Restructur* OR Chang*) N3 Environment*) OR MH "Cognitive Restructuring" OR TI (Cognitive N3 (Restructur* OR reapprais*)) OR TI refram* OR MH "Self Concept" OR TI "Self identit*" OR TI (Chang* N3 (Cognition* OR Emotion*)) OR MH "Self Regulation+" OR TI (Emotion* N3 Regulat*) OR TI (Shifting N3 Perspectiv*) OR TI (Valued N3 "self-identit*") OR TI (Mental N3 Rehears*) OR TI (Past N3 Succes*) OR TI (Pain N3 Belief*) OR MH "Patient Compliance" OR TI adherence OR TI complian* OR MH "Self-Efficacy" OR TI "Self concept*" OR TI "self efficacy" OR TI maintenance* OR TI maintain* OR TI relaps* OR TI recurren* OR TI exacerbation* OR TI ((Secundary OR Secondary) N3 Prevention*) OR MH "Health Behavior" OR MH "Attitude" OR MH "Attitude to Change" OR MH "Attitude to Health" OR MH "Attitude to Illness" OR MH "Attitude to Disability" OR TI "Attitud*" OR MH "Health Beliefs" OR TI ((Illness OR Disease* OR Sickness* OR Health) N3 (Behavior* OR Behaviour* OR Belief*)) OR TI "Risk reduction*") OR ( MH "Behavioral Changes" OR AB ((Behavior* OR Behaviour*) N3 (Change*)) OR MH "Goals and Objectives" OR MH "Behavioral Objectives" OR MH "Goal Attainment" OR MH "Goal-Setting" OR AB Goal* OR MH "Motivation" OR AB motivation OR AB ((Action OR transition) N3 Plan*) OR MH "Commitment" OR AB "Commit*" OR MH "Problem Solving" OR AB (Problem* N3 solv*) OR MH "Decision Making, Shared" OR MH "Decision Making, Patient" OR AB (Shared N3 Decision*) OR AB (Patient N3 (Participati* OR Involv* OR Engag* OR Activiti*)) OR AB (Implementation N3 Intention*) OR AB (Conservat* N3 ("Mental Resourc*" OR "Mental effort")) OR MH "Mental Fatigue" OR AB (Mental N3 Fatigue) OR AB (Monitor* N3 (Behavio* OR Self)) OR AB "selfmonitoring" OR MH "Support, Social+" OR MH "Social Comparison" OR MH "Social Attitudes" OR AB ((Social OR psychological* OR psychosocial OR "psycho-social" OR emotional* OR community) N3 (Support* OR Care OR Approv* OR Comparison*)) OR AB "support system*" OR AB antecedent* OR AB "re-attribution*" OR AB "reattribution*" OR AB ((Health OR Social OR Emotional) N3 Consequence*) OR AB (Anticipated N3 Regret*) OR (Future N3 Punishment*) OR AB ((Behavior OR Behaviour) N3 Substitution*) OR MH "Habits" OR AB (Habit N3 (Formation* OR Reversal)) OR AB generalisation* OR generalization* OR AB (Graded N3 (Task* OR Activit* OR exercise* OR therap*)) OR MH "Reward" OR AB reward* OR AB "self-incentive*" OR AB ((Restructur* OR Chang*) N3 Environment*) OR MH "Cognitive Restructuring" OR AB (Cognitive N3 (Restructur* OR reapprais*)) OR AB refram* OR MH "Self Concept" OR AB "Self identit*" OR AB (Chang* N3 (Cognition* OR Emotion*)) OR MH "Self Regulation+" OR AB (Emotion* N3 Regulat*) OR AB (Shifting N3 Perspectiv*) OR AB (Valued N3 "self-identit*") OR AB (Mental N3 Rehears*) OR AB (Past N3 Succes*) OR AB (Pain N3 Belief*) OR MH "Patient Compliance" OR AB adherence OR AB complian* OR MH "Self-Efficacy" OR AB "Self concept*" OR AB "self efficacy" OR AB maintenance* OR AB maintain* OR AB relaps* OR AB recurren* OR AB exacerbation* OR AB ((Secundary OR Secondary) N3 Prevention*) OR MH "Health Behavior" OR MH "Attitude" OR MH "Attitude to Change" OR MH "Attitude to Health" OR MH "Attitude to Illness" OR MH "Attitude to Disability" OR AB "Attitud*" OR MH "Health Beliefs" OR AB ((Illness OR Disease* OR Sickness* OR Health) N3 (Behavior* OR Behaviour* OR Belief*)) OR AB "Risk reduction*")

I4: Selfmanagement support

(MH "Self-Management"OR TI "self-manage*" OR TI selfmanage* OR TI "self-control*" OR MH "Self Regulation+" TI selfcontrol* OR TI selfregulation* OR TI "self-regulation*" OR TI "emotional regulation*" OR MH "Self Care" OR TI "self care" OR TI "self caring" OR TI "self help" OR MH "Empowerment" OR TI empower* OR MH "Patient Education" OR TI (Patient* N3 (Educat* OR Train*)) OR MH "Health Education" OR MH "Consumer Health Information+" OR TI ((Health OR Medical) N3 (Education OR Literac* OR Illiterac* OR Information* OR Knowledg* OR Skill* OR Comprehen* OR Attitude*)) OR TI "teach-back" OR TI "teach back" OR MH "Knowledge" OR MH "Health Knowledge" OR MH "Attitude to Health" OR MH "Education" OR MH "Education, Nonprofessional" OR TI diary OR TI diaries OR TI handout* OR TI "hand-out*" OR MH "Teaching Materials" OR TI (Teaching N3 Material*) OR MH "Books" OR TI book* OR MH "Medical Illustration" OR TI "medical illustration*" OR TI leaflet* OR MH "Audiovisuals" OR TI video* OR TI "audiovisual" OR TI "audio-visual" OR TI film OR TI films OR TI tutorial* OR TI webcast* OR TI podcast* OR TI youtube* OR TI inform* OR TI (Knowledge N3 (Disease* OR Ilness* OR Disorder*)) OR TI mentor* OR TI "pros and cons" OR TI (Decisional N3 Balanc*) OR TI (Comparative N3 Imag*) OR TI (Directive N3 Counseling) OR MH "Motivational Interviewing" OR TI (Motivational N3 Interview*) OR TI (Body N3 Awareness) OR TI (Active N3 Listening) OR TI (Relaxation N3 Techni*) OR TI (Pain N3 Provocation*) OR MH "Adaptation, Psychological" OR MH "Adaptation, Occupational" OR TI ((Psychological OR occupational OR work OR job) N3 (Adaptation* OR Adjustment*)) OR TI "adaptive behavio*" OR MH "Coping" OR MH "Help Seeking Behavior" OR MH "Stress Management" OR TI coping OR TI "stress management" OR TI (support N3 seeking) OR TI regulation OR MH "Life Style" OR TI (Life N3 Style*) OR TI "lifestyle*" OR MH "Cognitive Therapy" OR TI (Cognitive N3 Behavio* N3 (Therap* OR Treat*)) OR TI CBT OR MH "Health Belief Model" OR TI (Health N3 Belief*) OR MH "Transtheoretical Stages of Change Model" OR TI ((Transtheoretical OR trans-theoretical OR transactional OR "trans-actional") N3 Model*) OR TI TTM OR TI (Stages N3 Change*) OR MH "Models, Biopsychosocial" OR TI "biopsychosocial model*" OR TI "bio-psychosocial model*" OR TI prochaska* OR TI (Pain N3 Belief*) OR TI (internal N3 external N3 control*) OR TI ((Sense OR Locus) N3 Control*) OR MH "Health Promotion" OR TI (Health N3 Promotion*) OR TI (Attitude N3 (lllness* OR Disease* OR Disorder* OR health)) OR TI (Common N3 Sense N3 Model*) OR TI Huber* OR TI (Positive N3 Health) OR TI "behavior theor*" OR TI "behaviour theor*" OR MH "Leventhal and Johnson's Theory of Self-Regulation" OR MH "Lazarus Theory of Stress and Coping" OR MH "Ajzen-Fishbein Theory of Reasoned Action" OR MH "Ajzen's Theory of Planned Behavior" OR MH "Social Learning Theory+" OR TI ((Stress OR "Social Cognitive" OR "Social Learning" OR "Planned Behavio*" OR "Reasoned Action") N3 Theory)) OR (MH "Self-Management"OR AB "self-manage*" OR AB selfmanage* OR AB "self-control*" OR MH "Self Regulation+" AB selfcontrol* OR AB selfregulation* OR AB "self-regulation*" OR AB "emotional regulation*" OR MH "Self Care" OR AB "self care" OR AB "self caring" OR AB "self help" OR MH "Empowerment" OR AB empower* OR MH "Patient Education" OR AB (Patient* N3 (Educat* OR Train*)) OR MH "Health Education" OR MH "Consumer Health Information+" OR AB ((Health OR Medical) N3 (Education OR Literac* OR Illiterac* OR Information* OR Knowledg* OR Skill* OR Comprehen* OR Attitude*)) OR AB "teach-back" OR AB "teach back" OR MH "Knowledge" OR MH "Health Knowledge" OR MH "Attitude to Health" OR MH "Education" OR MH "Education, Nonprofessional" OR AB diary OR AB diaries OR AB handout* OR AB "hand-out*" OR MH "Teaching Materials" OR AB (Teaching N3 Material*) OR MH "Books" OR AB book* OR MH "Medical Illustration" OR AB "medical illustration*" OR AB leaflet* OR MH "Audiovisuals" OR AB video* OR AB "audiovisual" OR AB "audio-visual" OR AB film OR AB films OR AB tutorial* OR AB webcast* OR AB podcast* OR AB youtube* OR AB inform* OR AB (Knowledge N3 (Disease* OR Ilness* OR Disorder*)) OR AB mentor* OR AB "pros and cons" OR AB (Decisional N3 Balanc*) OR AB (Comparative N3 Imag*) OR AB (Directive N3 Counseling) OR MH "Motivational Interviewing" OR AB (Motivational N3 Interview*) OR AB (Body N3 Awareness) OR AB (Active N3 Listening) OR AB (Relaxation N3 Techni*) OR AB (Pain N3 Provocation*) OR MH "Adaptation, Psychological" OR MH "Adaptation, Occupational" OR AB ((Psychological OR occupational OR work OR job) N3 (Adaptation* OR Adjustment*)) OR AB "adaptive behavio*" OR MH "Coping" OR MH "Help Seeking Behavior" OR MH "Stress Management" OR AB coping OR AB "stress management" OR AB (support N3 seeking) OR AB regulation OR MH "Life Style" OR AB (Life N3 Style*) OR AB "lifestyle*" OR MH "Cognitive Therapy" OR AB (Cognitive N3 Behavio* N3 (Therap* OR Treat*)) OR AB CBT OR MH "Health Belief Model" OR AB (Health N3 Belief*) OR MH "Transtheoretical Stages of Change Model" OR AB ((Transtheoretical OR trans-theoretical OR transactional OR "trans-actional") N3 Model*) OR AB TTM OR AB (Stages N3 Change*) OR MH "Models, Biopsychosocial" OR AB "biopsychosocial model*" OR AB "bio-psychosocial model*" OR AB prochaska* OR AB (Pain N3 Belief*) OR AB (internal N3 external N3 control*) OR AB ((Sense OR Locus) N3 Control*) OR MH "Health Promotion" OR AB (Health N3 Promotion*) OR AB (Attitude N3 (lllness* OR Disease* OR Disorder* OR health)) OR AB (Common N3 Sense N3 Model*) OR AB Huber* OR AB (Positive N3 Health) OR AB "behavior theor*" OR AB "behaviour theor*" OR MH "Leventhal and Johnson's Theory of Self-Regulation" OR MH "Lazarus Theory of Stress and Coping" OR MH "Ajzen-Fishbein Theory of Reasoned Action" OR MH "Ajzen's Theory of Planned Behavior" OR MH "Social Learning Theory+" OR AB ((Stress OR "Social Cognitive" OR "Social Learning" OR "Planned Behavio*" OR "Reasoned Action") N3 Theory))

O1: Movement behaviour

( MH "Movement" OR "movement*" OR TI coordination* OR TI (Movement* N3 (Fluenc* OR symmetr* OR Asymmetr*)) OR TI compensat* OR TI "co-contraction" OR TI "cocontraction" OR MH "Muscle Tonus" OR TI ((Muscle OR Muscular) N3 (Tonus OR Tone OR Tones OR Tension OR Tightness* OR Contraction* OR Relaxation* OR Function*)) OR TI agonist* OR TI antagonist* OR MH "Muscle Contraction" OR TI Inotropism* OR MH "Muscle Relaxation" OR MH "Job Performance" OR TI ((job OR work OR occupational OR Task OR Tasks) N3 Performanc*) OR MH "Task Performance and Analysis" OR MH "Workload Measurement" OR TI (Task N3 Analy*) OR TI (workload N3 measurement*) OR TI (Critical N3 Incident N3 Techn*) OR MH "Balance, Postural+" OR TI (Postural N3 (Control* OR Equilibri*)) OR TI (Core N3 Stabilit*) OR TI balanc* OR TI unbalanc* OR TI align* OR MH "Ergonomics" OR TI ergonomic* OR MH "Environment" OR TI environment* OR TI ((Physical OR Sedentary) N3 Time) OR MH "Time Factors" OR TI (Minutes N3 (Week OR Day)) OR TI bout* OR MH "Life Style, Sedentary+" OR TI (Sedentary N3 (Behavio* OR Lifestyle* OR "life style*")) OR TI (Physical N3 Inactivit*) OR MH "Heart Rate" OR TI ((Heart OR Cardiac OR Pulse*) N3 (Rate* OR Beat*)) OR TI HRR OR TI MET OR TI (Metabolic N3 Equivalent) OR TI ((Light OR High OR Moderate OR Vigorous) N3 Intensit*) OR TI ((Physical OR functional) N3 Activit*) OR TI pace OR TI paces OR TI speed* OR TI velocity OR MH "Acceleration (Mechanics)" OR MH "Acceleration (Physiology)" OR TI accelerat* OR MH "Kinematics" OR TI kinematics OR MH "Range of Motion" OR TI (Range N3 Motion*) OR TI ROM OR TI (flexibilit* N3 joint*) OR MH "Physical Performance" OR TI ((functional OR physical) N3 performanc*)) OR ( MH "Movement" OR "movement*" OR AB coordination* OR AB (Movement* N3 (Fluenc* OR symmetr* OR Asymmetr*)) OR AB compensat* OR AB "co-contraction" OR AB "cocontraction" OR MH "Muscle Tonus" OR AB ((Muscle OR Muscular) N3 (Tonus OR Tone OR Tones OR Tension OR Tightness* OR Contraction* OR Relaxation* OR Function*)) OR AB agonist* OR AB antagonist* OR MH "Muscle Contraction" OR AB Inotropism* OR MH "Muscle Relaxation" OR MH "Job Performance" OR AB ((job OR work OR occupational OR Task OR Tasks) N3 Performanc*) OR MH "Task Performance and Analysis" OR MH "Workload Measurement" OR AB (Task N3 Analy*) OR AB (workload N3 measurement*) OR AB (Critical N3 Incident N3 Techn*) OR MH "Balance, Postural+" OR AB (Postural N3 (Control* OR Equilibri*)) OR AB (Core N3 Stabilit*) OR AB balanc* OR AB unbalanc* OR AB align* OR MH "Ergonomics" OR AB ergonomic* OR MH "Environment" OR AB environment* OR AB ((Physical OR Sedentary) N3 Time) OR MH "Time Factors" OR AB (Minutes N3 (Week OR Day)) OR AB bout* OR MH "Life Style, Sedentary+" OR AB (Sedentary N3 (Behavio* OR Lifestyle* OR "life style*")) OR AB (Physical N3 Inactivit*) OR MH "Heart Rate" OR AB ((Heart OR Cardiac OR Pulse*) N3 (Rate* OR Beat*)) OR AB HRR OR AB MET OR AB (Metabolic N3 Equivalent) OR AB ((Light OR High OR Moderate OR Vigorous) N3 Intensit*) OR AB ((Physical OR functional) N3 Activit*) OR AB pace OR AB paces OR AB speed* OR AB velocity OR MH "Acceleration (Mechanics)" OR MH "Acceleration (Physiology)" OR AB accelerat* OR MH "Kinematics" OR AB kinematics OR MH "Range of Motion" OR AB (Range N3 Motion*) OR AB ROM OR AB (flexibilit* N3 joint*) OR MH "Physical Performance" OR AB ((functional OR physical) N3 performanc*))

O2: Activities of daily living

(MH "Severity of Disability" OR MH "Persons with Disabilities" OR TI disabilit* OR MH "International Classification of Functioning, Disability, and Health" OR TI ICF OR TI (mobility N3 limit*) OR MH "Activities of Daily Living+" OR TI (Daily N3 (Activit* OR Life OR Living OR Lives)) OR TI ADL OR TI everyday OR TI "every day" OR MH "Functional Status" OR TI (Functional N3 (Status OR Dependen* OR Indepen*)) OR MH "Work" OR MH "Job Re-Entry" OR MH "Work-Life Balance" OR MH "Work Environment" OR TI work* OR TI job OR TIjobs OR TI Commut* OR TI sport* OR TI leisure* OR TI hobb* OR MH "Home Environment" OR TI home* OR TI (Living N3 Alone) OR MH "Schools" OR TI school* OR MH "Students" OR TI student* OR MH "Child Care" OR TI ((Child OR Day OR Informal)N3 Care) OR TI puericulture OR TI caregiv* OR TI (Care N3 Giv*) OR MH "Volunteer Workers" OR TI volunteer* OR MH "Social Participation" OR MH "Sports Participation" OR TI ((Social* OR sport*) N3 (Participati* OR involv* OR engag*)) OR ((MH "Posture" OR TI postur* OR MH "Body Positions" OR MH "Standing" OR TI stand OR TI standing OR MH "Sitting" OR TI sit OR TI sitting OR TI "seated" OR TI recumben* OR TI lying OR MH "Prone Position" OR TI (Prone N3 Position*) OR MH "Supine Position" OR TI (Supine N3 Position*) OR TI ambulat* OR MH "Gait+" OR TI gait* OR MH "Walking" OR TI walk* OR TI step* OR TI stoop* OR TI bend* OR MH "Squatting" OR TI squat* OR TI push* OR TI pull* OR TI ((Golf OR Golfer) N3 Techn*) OR MH "Lifting" OR TI wlt OR TI "lift*" OR TI "carry*" OR TI jump* OR MH "Stair Climbing" OR TI (Stair N3 (Climb* OR Navigat*)) OR MH "Rising" OR TI rising OR TI rise OR TI (sit N3 stand) OR TI (Chair N3 Bed) OR TI reach* OR MH "Throwing" OR TI throw* OR MH "Locomotion" OR TI locomot* OR MH "Running" OR TI run OR TI running OR MH "Jogging" OR TI jog OR TI jogging OR TI (Head N3 (Movement* OR Moving*))) AND TI (difficult*:ti,ab,kw OR impairment*:ti,ab,kw OR limit*:ti,ab,kw OR problem*:ti,ab,kw))) OR (MH "Severity of Disability" OR MH "Persons with Disabilities" OR AB disabilit* OR MH "International Classification of Functioning, Disability, and Health" OR AB ICF OR AB (mobility N3 limit*) OR MH "Activities of Daily Living+" OR AB (Daily N3 (Activit* OR Life OR Living OR Lives)) OR AB ADL OR AB everyday OR AB "every day" OR MH "Functional Status" OR AB (Functional N3 (Status OR Dependen* OR Indepen*)) OR MH "Work" OR MH "Job Re-Entry" OR MH "Work-Life Balance" OR MH "Work Environment" OR AB work* OR AB job OR ABjobs OR AB Commut* OR AB sport* OR AB leisure* OR AB hobb* OR MH "Home Environment" OR AB home* OR AB (Living N3 Alone) OR MH "Schools" OR AB school* OR MH "Students" OR AB student* OR MH "Child Care" OR AB ((Child OR Day OR Informal)N3 Care) OR AB puericulture OR AB caregiv* OR AB (Care N3 Giv*) OR MH "Volunteer Workers" OR AB volunteer* OR MH "Social Participation" OR MH "Sports Participation" OR AB ((Social* OR sport*) N3 (Participati* OR involv* OR engag*)) OR ((MH "Posture" OR AB postur* OR MH "Body Positions" OR MH "Standing" OR AB stand OR AB standing OR MH "Sitting" OR AB sit OR AB sitting OR AB "seated" OR AB recumben* OR AB lying OR MH "Prone Position" OR AB (Prone N3 Position*) OR MH "Supine Position" OR AB (Supine N3 Position*) OR AB ambulat* OR MH "Gait+" OR AB gait* OR MH "Walking" OR AB walk* OR AB step* OR AB stoop* OR AB bend* OR MH "Squatting" OR AB squat* OR AB push* OR AB pull* OR AB ((Golf OR Golfer) N3 Techn*) OR MH "Lifting" OR AB wlt OR AB "lift*" OR AB "carry*" OR AB jump* OR MH "Stair Climbing" OR AB (Stair N3 (Climb* OR Navigat*)) OR MH "Rising" OR AB rising OR AB rise OR AB (sit N3 stand) OR AB (Chair N3 Bed) OR AB reach* OR MH "Throwing" OR AB throw* OR MH "Locomotion" OR AB locomot* OR MH "Running" OR AB run OR AB running OR MH "Jogging" OR AB jog OR AB jogging OR AB (Head N3 (Movement* OR Moving*))) AND AB (difficult*:ti,ab,kw OR impairment*:ti,ab,kw OR limit*:ti,ab,kw OR problem*:ti,ab,kw)))

FILTERS

RCT blok: 530,628 results 19-12-2022

AND (MH "Clinical Trials" OR MH "Randomized Controlled Trials+" OR MH "Nonrandomized Trials" OR MH "Random Sample+" OR MH "Single-Blind Studies" OR MH "Double-Blind Studies" OR MH "Triple-Blind Studies" OR MH "Quasi-Experimental Studies" OR MH "Controlled Before-After Studies" OR TI (rct OR random* OR trial* OR "single-blind*" OR "single-masked" OR "double-blind*" OR "double masked" OR "triple-blind*" OR "triple-masked" OR "quasi-experimental") OR MH "Pretest-Posttest Design+" OR TI ("pre-test" OR "pretest" OR "post-test" OR "posttest"))

NOT TI protocol
